# Treatment needs of dementia with Lewy bodies according to patients, caregivers, and physicians: a cross-sectional, observational, questionnaire-based study in Japan

**DOI:** 10.1186/s13195-022-01130-4

**Published:** 2022-12-15

**Authors:** Mamoru Hashimoto, Yuta Manabe, Takuhiro Yamaguchi, Shunji Toya, Manabu Ikeda

**Affiliations:** 1 Department of Psychiatry, Osaka University Graduate School of Medicine, Osaka, Japan; 2 Department of Neuropsychiatry, Kindai University Faculty of Medicine, Osakasayama, Japan; 3 Department of Dementia and Geriatric Medicine, Division of Clinical Science, Kanagawa Dental University School of Dentistry, Yokosuka, Japan; 4 Division of Biostatistics, Tohoku University Graduate School of Medicine, Sendai, Japan; 5 Medical Affairs, Sumitomo Pharma Co., Ltd., Tokyo, Japan

**Keywords:** Dementia with Lewy bodies, Treatment needs of patients, Treatment needs of caregivers, Understanding of treatment needs of patients and caregivers, Questionnaire survey

## Abstract

**Background:**

Understanding the treatment needs of patients with dementia with Lewy bodies (DLB) is essential to develop treatment strategies. We examined the treatment needs of patients with DLB and their caregivers and the extent to which the attending physicians understand these treatment needs.

**Methods:**

This was a cross-sectional, observational study conducted using questionnaires for patients, caregivers, and physicians. The study participants included patients, their caregivers, and their attending physicians who were experts in DLB. Fifty-two symptoms that are frequent and clinically important in DLB were pre-selected and classified into seven symptom domains. Treatment needs of patients and caregivers were defined as “symptom that causes them most distress,” and the frequency of each answer was tabulated. To assess the physician’s understanding of the treatment needs of patients and caregivers, patient–physician and caregiver–physician concordance rates for each answer regarding treatment needs were calculated according to symptom domains.

**Results:**

In total, 263 pairs of patients–caregivers and 38 physicians were surveyed. The mean age of patients was 79.3 years, and their mean total score on the Mini-Mental State Examination was 20.9. Thirty-five and 38 symptoms were selected as symptoms causing patients and caregivers most distress, respectively. Memory impairment was most frequently selected for the treatment needs of patients, followed by constipation and bradykinesia. Memory impairment was also most frequently selected by caregivers, followed by visual hallucinations. For the symptom domain that causes patients or caregivers most distress, only about half of the patient–physician pairs (46.9%) and caregiver–physician pairs (50.8%) were matched. Logistic regression analysis identified that concordance rates for treatment needs between patient–physician and caregiver–physician were lower when autonomic dysfunction and sleep-related disorders were selected as the symptom domains that cause most distress.

**Conclusion:**

There was considerable variability in the treatment needs of patients with DLB and their caregivers. Attending physicians had difficulty understanding the top treatment needs of their patients and caregivers, despite their expertise in DLB, because of various clinical manifestations. Attending physicians should pay more attention to autonomic dysfunction and sleep-related disorders in the treatment of DLB.

**Trial registration:**

UMIN Clinical Trials Registry, UMIN000041844. Registered on 23 September 2020

**Supplementary Information:**

The online version contains supplementary material available at 10.1186/s13195-022-01130-4.

## Background

Lewy body dementia consists of dementia with Lewy bodies (DLB) and Parkinson's disease with dementia, and is the second most common degenerative dementia in old age after Alzheimer’s disease (AD) [1–3]. In addition to progressive cognitive impairment, DLB is characterized by various clinical manifestations such as behavioral and psychological symptoms of dementia (BPSD) including visual hallucinations and depression, rapid eye movement sleep behavior disorder, parkinsonism, and autonomic dysfunction [4]. The frequency and time of onset of these clinical symptoms vary from patient to patient, and one patient can have multiple symptoms at the same time [5]. Therefore, the physician needs to focus on a wide variety of symptoms when treating patients with DLB.

When treating the various symptoms of patients with DLB, the treatment of one symptom of DLB may exacerbate other symptoms [6, 7]. For example, some antiparkinsonian drugs may exacerbate psychotic symptoms [8], and antipsychotic drugs may exacerbate parkinsonism [9]. These limitations make it difficult to respond to all symptoms in patients with DLB, resulting in a need to prioritize the symptoms of greatest relevance to each patient’s quality of life. However, the published information on DLB management, including pharmacological and non-pharmacological treatment, does not fully take into account the complex treatment limitations [4, 10, 11].

In developing treatment strategies for patients with DLB, one of the most important issues that attending physicians should focus on is the treatment needs of patients and their caregivers. The importance of patient-reported outcomes and caregiver-reported outcomes has been recognized in recent years and both have been used in clinical trials. Furthermore, combining both patient- and caregiver-reported outcomes with conventionally used assessments may help physicians improve their understanding of the severity of AD and the efficacy of treatment [12]. In amyotrophic lateral sclerosis, evaluating both patient- and caregiver-reported outcomes can help attending physicians determine when to start drug intervention [13]. These reports suggest that understanding patients’ and caregivers’ treatment needs is crucial in determining a treatment plan for a chronic, refractory disease such as DLB. Therefore, attending physicians need to know which symptoms most patients with DLB and their caregivers have trouble with and which symptoms they would like to see treated as a priority.

Previous studies have mainly focused on caregiver burden/stress among those caring for DLB and AD patients [14–18]; the caregiver burden/stress among those caring for patients with DLB was found to be higher than that of those caring for AD patients, and this burden/stress was found to be associated with BPSD, cognitive fluctuations, and the decline of activities of daily living (ADL) [14–16]. Based on these previous studies, BPSD and ADL impairments, which are associated with high caregiver burden, may be considered as caregivers’ treatment needs, but the treatment needs of the patients themselves cannot be known from these studies. Few studies have investigated the unmet needs of both patients with DLB and their caregivers [19, 20]. In one study [19], BPSD and ADL impairments were considered as treatment needs of caregivers due to the high caregiver burden. In another study [20], the sample sizes were small, and only three patients with DLB and 122 caregivers were interviewed. Therefore, the treatment needs of patients with DLB from the patient perspective are unclear from these previous studies. Furthermore, it is not clear to what extent attending physicians understand the treatment needs of individual patients with DLB and their caregivers in clinical practice.

The present study aimed to clarify (1) the treatment needs of patients with DLB and their caregivers, (2) the extent to which the attending physicians understand the treatment needs of their patients with DLB and their caregivers, and (3) what factors contribute to the lack of understanding of the attending physicians regarding the treatment needs of their patients with DLB and their caregivers.

## Methods

### Study design and participants

The present study was a cross-sectional, observational study conducted using a questionnaire. This study was conducted from September 2020 to July 2021 at 35 study sites with expert physicians in DLB treatment in Japan. The study participants included patients with DLB, their caregivers, and their attending physicians. Major inclusion criteria for patients with DLB were patients aged ≥50 years who met the diagnostic criteria for probable DLB [4], attending outpatient visits, under the care of their attending physician for at least 3 months, and with a reliable caregiver. The following patients were excluded from the study: patients with Parkinson’s disease with dementia (if parkinsonism had been present for more than 1 year prior to the onset of dementia) [21], patients whose attending physician had not seen them for more than 3 months prior to obtaining consent, and patients whose attending physician had deemed them inappropriate for participation in this study.

The main selection criteria for caregivers were aged ≥20 years and the primary caregiver (defined as the main person responsible for the care of the patient with DLB). Attending physicians were required to be experts in DLB treatment in Japan, defined as those who met at least one of the following criteria: (1) a representative organizer or an organizer of Dementia with Lewy Bodies Society Japan [22] (established by Dr. Kenji Kosaka in 2007 to promote clinical and basic research for DLB and provide better care to the many patients and their caregivers suffering from DLB); (2) an advisor or cooperating physician in the DLB Support Network in Japan (an organization for patients with DLB, caregivers, family members, and medical staff that provides information about DLB disease and care) [23]; (3) an author or co-author of a review article or research paper on DLB; and (4) any physician affiliated with the same department at the facility where the physician mentioned in (1), (2), or (3) works.

### Symptoms and symptom domains of DLB

In this study, a questionnaire survey was conducted to investigate the symptom that causes the patient/caregiver the most distress and the symptom the patient/caregiver would most likely prioritize for receiving treatment. For this purpose, we pre-selected 52 symptoms that are frequent and clinically important in DLB, and further classified these symptoms into seven symptom domains (Table 1). Note that eating behavior-related problems and sleep-related disorders were classified independently from psychiatric symptoms. An explanatory table was prepared for each of the 52 clinical symptoms and included in the questionnaire (see Supplementary Table 1, Additional file 1). In addition, a 15-min movie explaining the symptoms of DLB was created. This focused on the explanations of the seven symptom domains and symptoms that were difficult for patients and caregivers to understand (e.g., disorientation, attention dysfunction, hallucinations other than visual hallucinations, apathy, orthostatic hypotension, and syncope) and those that were difficult to distinguish (e.g., action tremor and rest tremor).

**Table 1:** Seven symptom domains and 52 symptoms

Symptom domain	Number of symptoms	Symptoms
Cognitive impairment	7	Memory impairment, disorientation, executive dysfunction, attention dysfunction, fluctuating cognition, visuospatial dysfunction, other cognitive impairments
Parkinsonism	11	Bradykinesia/akinesia, rigidity, action tremor, rest tremor, postural instability, gait disturbance (short-stepped gait), freezing of gait, abnormal posture, salivation, fall, dysphagia
Psychiatric symptoms except for sleep disturbance and abnormal eating behavior	12	Delusions, visual hallucinations, hallucinations other than visual hallucinations, agitation/aggression, depression, anxiety, apathy, disinhibition, aberrant motor behavior, negativism, delirium, other psychiatric symptoms
Eating behavior-related problems	7	Loss of appetite, increase in appetite, weight loss, weight gain, food refusal, eating non-edible things, unbalanced diet
Sleep-related disorders	7	Rapid eye movement sleep behavior disorder, daytime somnolence, day–night reversal, nighttime sleep disorder, sudden sleep, restless legs syndrome, periodic limb movement disorder
Autonomic dysfunction	7	Orthostatic hypotension, disturbance of sweating, constipation, nighttime dysuria, daytime dysuria, syncope, dizziness
Sensory disorders	1	Dysosmia

### Contents of the questionnaires

The questionnaire for patients had a maximum of 40 questions (see Supplementary Methods 1, Additional file 2), including questions such as “Please select only one symptom that currently causes you the most distress” and “As you continue with treatment, which symptom would you most likely prioritize for receiving treatment? Please select only one.”

The questionnaire for caregivers had a maximum of 78 questions (see Supplementary Methods 2, Additional file 3), including questions such as “Please select only one symptom of the patient that currently causes you the most distress,” “As the patient continues with treatment, which patient’s symptom would you most likely prioritize for receiving treatment? Please select only one,” “Please select only one symptom that currently causes the patient the most distress,” and “As the patient continues with treatment, which patient’s symptom do you think the patient would most likely prioritize for receiving treatment? Please select only one.”

The questionnaire for physicians had two parts, which were answered by participating physicians via the web. The first part of the questionnaire consisted of 11 questions, including age, acquisition status of medical specialty, the number of patients with DLB who received treatment, and approach to general medical care and treatment for DLB (see Supplementary Methods 3, Additional file 4). The second part of the questionnaire was designed to examine whether the attending physician understands the treatment needs of patients with DLB and their caregivers and consisted of up to 114 questions, including questions such as “Please select only one symptom domain you think currently causes your patient the most distress,” “Please select only one symptom domain of your patient you think currently causes your patient’s caregiver the most distress,” “As your patient continues with treatment, which symptom domain do you think they would most likely prioritize for receiving treatment? Please select only one applicable symptom domain,” and “As your patient continues with treatment, which patient’s symptom do you think your patient’s caregiver would most likely prioritize for receiving treatment? Please select only one applicable symptom domain” (see Supplementary Methods 4, Additional file 5). The physician selected one applicable symptom domain, followed by the selection of symptoms from the selected domain.

### Assessments

Patients and caregivers who participated in this study underwent several screening tests. The degree of patients’ cognitive impairment, ADL, and parkinsonism were assessed with the Japanese version of the Mini-Mental State Examination (MMSE-J) [24] and the Japanese version of the Movement Disorder Society-Unified Parkinson’s Disease Rating Scale (MDS-UPDRS) [25] Part II and III, respectively. To assess BPSD and cognitive fluctuations of patients with DLB, the Japanese version of the Neuropsychiatric Inventory-12 (NPI-12) [26] and the Cognitive Fluctuation Inventory (CFI) [27], respectively, were administered to caregivers. In addition, a shortened Japanese version of the Zarit Caregiver Burden Interview (J-ZBI_8) [28] consisting of eight questions was used to assess caregiver burden. Of these screening tests, the MDS-UPDRS Part III was administered by physicians who had experience using the UPDRS or MDS-UPDRS or had received training provided by the Movement Disorder Society for the use of the MDS-UPDRS Part III. Patients and caregivers also answered the Short Form-8 (SF-8) [29] to assess their quality of life. Patient answers for the SF-8 could be filled out by their caregivers.

All these screening tests were performed after consent was obtained. Next, patients and their caregivers watched the movie explaining the symptoms of DLB. Finally, they filled out the respective questionnaires for patients and caregivers, which were sent to the study office within 3 weeks. To avoid the possibility of inaccurate patient answers due to cognitive impairment or tremor, the caregivers were allowed to assist their patients in filling out the questionnaire. However, in such cases, caregivers had to fill out their questionnaire first, followed by the patient’s questionnaire, and they had to report that they had assisted the patient. Physicians filled out the first part of the questionnaire consisting of 11 questions at the time of participation in this study. Thereafter, each time a patient and caregiver were enrolled, they answered the second part of the questionnaire, consisting of up to 114 questions, via the web.

### Outcomes

In this study, treatment needs of patients and caregivers were defined as “symptom that causes patients or caregivers most distress” and “symptom that patients or caregivers would most likely prioritize for receiving treatment,” and the frequency of each answer was tabulated. Next, to assess the attending physician’s understanding of their wishes, we calculated the concordance rate with the following two indicators:

1. The patient–physician concordance rate for “symptom domain that causes the patient most distress according to the patient”
2. The caregiver–physician concordance rate for “symptom domain that causes the caregiver most distress according to the caregiver”

In addition, to assess the difference in whether patients answered themselves or with the assistance of their caregiver, we compared the patient–caregiver concordance rate for “symptom domain that causes the patient most distress” between with or without caregiver’s assistance.

The concordance rate was defined as “the number of pairs whose answers matched/the number of all analyzed pairs × 100” for each question. In the calculation of the concordance rate, the number of all analyzed pairs was defined as the number of pairs in which both parties (pairs of patient and physician, or caregiver and physician) had valid answers to the question in the questionnaire (excluding unanswered questions or multiple answers to single answers).

### Statistical analysis

Descriptive statistics were used to describe the background characteristics of the study participants (patients, caregivers, and physicians), the patient’s or caregiver’s most inconvenient symptom domain and symptom, and the patient’s or caregiver’s main symptom or symptom domain for which treatment is desired. Summary statistics were calculated as mean ± standard deviation for continuous scales and frequency and percentage for nominal scales.

Point estimates and 95% confidence intervals (CIs) were calculated for the concordance rates between patients and physicians and between caregivers and physicians for each question, and kappa (*k*) coefficients were calculated as an index of the degree of concordance. A kappa score less than 0.4 was classified as poor; 0.4 to less than 0.6, moderate; 0.6 to less than 0.8, good; and 0.8 or greater, excellent [30]. In addition, the symmetry assessment using the McNemar–Bowker test was performed for agreement status in the questions for symptom domains.

To determine if there was a difference in the concordance rate for the “patient’s most inconvenient symptom domain” depending on which domain the patient chose, the patient–physician concordance rate was analyzed using Fisher’s exact test across the seven symptom domains. The caregiver–physician concordance rate for the “caregiver’s most inconvenient symptom domain” was also analyzed across the seven symptom domains. Shaffer’s method was used to correct for multiple comparisons among the seven groups.

In addition, to identify factors that would decrease the concordance rate (i.e., increase the discordance rate), a logistic regression analysis was performed with the discordance of answers as the dependent variable (discordance = 1, concordance = 0) and each background factor as an independent variable. Because there was a wide range of independent variables to be explored, extracting factors with *p* < 0.05 in the univariate analysis, the multivariate model was applied by the stepwise method with likelihood ratios. Details of the methods used for the univariate analysis are described in Supplementary Methods 5 (Additional file 6). The statistical significance level in this study was set at a two-sided significance level of 0.05, and all analyses were performed using SAS version 9.4 (SAS Institute Inc., Cary, NC, USA).

## Results

### Participants’ characteristics

**Fig. 1:**
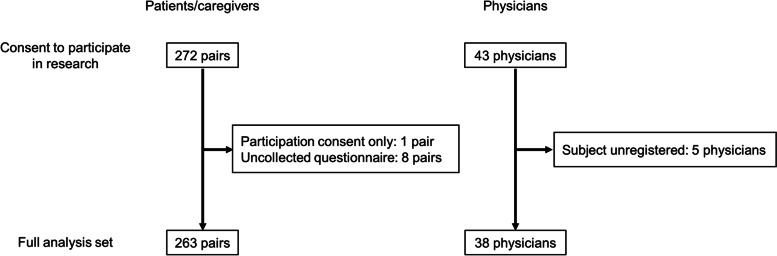
Subject disposition

Figure 1 shows the subject disposition. Of 272 pairs of patients and caregivers who consented to participate in this study, eight pairs who did not submit the questionnaires and one pair that only provided consent were excluded, resulting in 263 pairs analyzed. Of 43 attending physicians who consented to participate in this study, 5 who had no enrolled patients and caregivers were excluded, resulting in 38 physicians analyzed.

The background characteristics of patients, caregivers, and physicians in this study are summarized in Table 2. The mean age of patients with DLB was 79.3 ± 6.7 years, and the mean duration of DLB was 30.4 ± 29.7 months. The mean total scores of MMSE-J, NPI-12, and MDS-UPDRS Part III were 20.9 ± 5.9, 16.0 ± 16.6, and 23.8 ± 20.6, respectively. Among the 263 patients evaluated, the number of patients with MMSE-J score ≤19 (moderate cognitive impairment) was 95 (36.1%) and that of patients with MMSE-J score ≥20 (mild cognitive impairment) was 168 (63.9%). Psychiatry was the most common clinical department visited by patients (51.0%), followed by geriatric medicine (24.7%) and neurology (18.6%). Most patients were residing at home, and only 11 patients (4.2%) were residing in nursing homes. The mean age of the caregivers was 64.8 ± 12.8 years, most were female (72.6%), and the most common relationships with the patients were spouse (52.9%), followed by a son or daughter (39.5%). Most caregivers (80.2%) were living with the patient, and the mean time per day spent with the patient by caregivers who lived with the patient was 14.5 ± 8.9 h. The mean age of the physicians was 51.1 ± 7.8 years. The most common affiliated clinical department was psychiatry (69.4%), and more than half of the physicians had treated more than 100 patients with DLB.

**Table 2:** Background characteristics of patients, caregivers, and physicians

Patients (*N* = 263)		Caregivers (*N* = 263)		Physicians (*N* = 38)	
Age (y)	79.3 ± 6.7	Age (y)	64.8 ± 12.8	Age (y)	51.1 ± 7.8
Sex (M/F)	128/135	Sex (M/F)	72/191	Sex (M/F)	32/6
DLB duration (m)	30.4 ± 29.7 (*n* = 260)	Relationship with patient		Affiliated clinical department	
				Psychiatry	26 (69.4)
Education history (y)	11.8 ± 2.8 (*n* = 242)	Spouse	139 (52.9)	Neurology	6 (15.8)
Institute		Father or mother	0 (0.0)	Geriatric medicine	4 (10.5)
University hospital	123 (46.8)	Sibling	4 (1.5)	Neurosurgery	1 (2.6)
Non-university hospital	51 (19.4)	Son or daughter	104 (39.5)	Others	1 (2.6)
Clinic	89 (33.8)	Son- or daughter-in-law	12 (4.6)	Number of patients with DLB treated by the physician	
Clinical department		Grandchild	2 (0.8)	Less than 10	0 (0.0)
Psychiatry	134 (51.0)	Care provider	2 (0.8)	Between 10 and 30	5 (13.2)
Neurology	49 (18.6)	Living with the patient, yes	211 (80.2)	Between 30 and 100	12 (31.6)
Geriatric medicine	65 (24.7)	Time spent with the patient (h/d)	14.5 ± 8.9 (*n* = 258)	More than 100	21 (55.3)
Neurosurgery	1 (0.4)	Presence of assistant caregiver, yes	91 (34.6)	Inclusion criteria	
Others	14 (5.3)	Working, yes	111 (42.2)	1) A representative organizer or an organizer of Dementia with Lewy Bodies Society Japan	8 (15.8)
Living alone, yes	25 (10.2) (*n* = 245)	J-ZBI_8 total score	8.3 ± 6.3		
MMSE-J score	20.9 ± 5.9	J-ZBI_8 personal strain	6.0 ± 4.5	2) An advisor or cooperating physician in the DLB Support Network in Japan	12 (31.6)
≤19 (moderate cognitive impairment)	95 (36.1)				
≥20 (mild cognitive impairment)	168 (63.9)				
NPI-12 total score	16.0 ± 16.6 (*n* = 261)	J-ZBI_8 role strain	2.3 ± 2.7	3) An author or co-author of a review article or research paper on DLB	30 (78.9)
MDS-UPDRS Part III total score	23.8 ± 20.6 (*n* = 261)	SF-8 PCS	48.7 ± 7.3 (*n* = 254)	4) Any physician affiliated with the same department at the facility where the physician mentioned in (1), (2), or (3) works	0 (0.0)
MDS-UPDRS Part II total score	11.3 ± 10.8 (*n* = 262)	SF-8 MCS	47.9 ± 6.8 (*n* = 254)		
CFI	2.3 ± 3.0				
SF-8 PCS	45.4 ± 8.2 (*n* = 257)				
SF-8 MCS	48.7 ± 7.0 (*n* = 257)				

Data are expressed as mean ± SD, *n/n*, or *n* (%)

*Abbreviations*: *CFI* Cognitive Fluctuation Inventory, *DLB* dementia with Lewy bodies, *F* female, *J-ZBI_8* shortened Japanese version of the Zarit Caregiver Burden Interview, *M* male, *MCS* mental component summary, *MDS-UPDRS* Movement Disorder Society-Unified Parkinson’s Disease Rating Scale, *MMSE-J* Japanese version of the Mini-Mental State Examination, *NPI-12* Japanese version of the Neuropsychiatric Inventory-12, *PCS* physical component summary, *SD* standard deviation, *SF-8* Short Form-8

### Symptoms that cause the patient most distress and symptoms that the patient would most likely prioritize for receiving treatment

Table 3 shows the survey results of symptoms that cause the patient most distress and symptoms that the patient would most likely prioritize for receiving treatment. The number of valid answers for “symptom that causes the patient most distress” and “as the patient continues with treatment, symptom the patient would most likely prioritize for receiving treatment” were 160 (60.3%) and 148 (56.3%), respectively. Multiple choice answers were the most common reason for invalid answers (22.8%, 22.4%), followed by do not know (11.8%, 15.6%) and unanswered (4.6%, 5.7%). Symptoms that cause the patient most distress, in descending order of frequency, were memory impairment (8.7%), constipation (7.6%), bradykinesia/akinesia (6.5%), visual hallucinations (3.0%), and nighttime sleep disorder (2.7%). The symptoms that the patient would most likely prioritize for receiving treatment, in descending order, were memory impairment (11.4%), bradykinesia/akinesia (7.6%), abnormal posture (3.0%), constipation (2.7%), nighttime dysuria (2.7%), nighttime sleep disorder (2.7%), and visual hallucinations (2.7%).

**Table 3:** Symptoms causing patients most distress and symptoms that patients would most likely prioritize for treatment

Symptoms that cause the patient most distress (*N =* 263)		Symptoms that the patient would most likely prioritize for receiving treatment (*N =* 263)	
**Symptom domain**	***n*** **(%)**	**Symptom domain**	***n*** **(%)**
Parkinsonism	46 (17.5)	Parkinsonism	49 (18.6)
Cognitive impairment	40 (15.2)	Cognitive impairment	47 (17.9)
Autonomic dysfunction	35 (13.3)	Autonomic dysfunction	21 (8.0)
Psychiatric symptoms	19 (7.2)	Sleep-related disorders	15 (5.7)
Sleep-related disorders	15 (5.7)	Psychiatric symptoms	12 (4.6)
Eating behavior-related problems	5 (1.9)	Eating behavior-related problems	4 (1.5)
Sensory disorders	0 (0.0)	Sensory disorders	0 (0.0)
**Symptom**	***n*** **(%)**	**Symptom**	***n*** **(%)**
Memory impairment	23 (8.7)	Memory impairment	30 (11.4)
Constipation	20 (7.6)	Bradykinesia/akinesia	20 (7.6)
Bradykinesia/akinesia	17 (6.5)	Abnormal posture	8 (3.0)
Visual hallucinations	8 (3.0)	Constipation	7 (2.7)
Nighttime sleep disorder	7 (2.7)	Nighttime dysuria	7 (2.7)
Abnormal posture	6 (2.3)	Nighttime sleep disorder	7 (2.7)
Gait disturbance	6 (2.3)	Visual hallucinations	7 (2.7)
Nighttime dysuria	6 (2.3)	Attention dysfunction	6 (2.3)
Rapid eye movement sleep behavior disorder	6 (2.3)	Gait disturbance	6 (2.3)
Daytime dysuria	5 (1.9)	Rapid eye movement sleep behavior disorder	6 (2.3)
Anxiety	4 (1.5)	Action tremor	5 (1.9)
Attention dysfunction	4 (1.5)	Disorientation	4 (1.5)
Other cognitive impairment	4 (1.5)	Fall	3 (1.1)
Postural instability	4 (1.5)	Other cognitive impairment	3 (1.1)
Action tremor	3 (1.1)	Daytime dysuria	2 (0.8)
Disorientation	3 (1.1)	Daytime somnolence	2 (0.8)
Disturbance of sweating	3 (1.1)	Disturbance of sweating	2 (0.8)
Executive dysfunction	3 (1.1)	Fluctuating cognition	2 (0.8)
Freezing of gait	3 (1.1)	Freezing of gait	2 (0.8)
Hallucinations other than visual hallucinations	3 (1.1)	Hallucinations other than visual hallucinations	2 (0.8)
Salivation	3 (1.1)	Orthostatic hypotension	2 (0.8)
Depression	2 (0.8)	Postural instability	2 (0.8)
Fall	2 (0.8)	Weight loss	2 (0.8)
Fluctuating cognition	2 (0.8)	Agitation/aggression	1 (0.4)
Weight gain	2 (0.8)	Anxiety	1 (0.4)
Weight loss	2 (0.8)	Delusions	1 (0.4)
Day–night reversal	1 (0.4)	Dizziness	1 (0.4)
Daytime somnolence	1 (0.4)	Dysphagia	1 (0.4)
Delusions	1 (0.4)	Executive dysfunction	1 (0.4)
Loss of appetite	1 (0.4)	Loss of appetite	1 (0.4)
Orthostatic hypotension	1 (0.4)	Rigidity	1 (0.4)
Other psychiatric symptoms	1 (0.4)	Salivation	1 (0.4)
Rest tremor	1 (0.4)	Visuospatial dysfunction	1 (0.4)
Rigidity	1 (0.4)	Weight gain	1 (0.4)
Visuospatial dysfunction	1 (0.4)	Aberrant motor behavior	0 (0.0)
Aberrant motor behavior	0 (0.0)	Apathy	0 (0.0)
Agitation/aggression	0 (0.0)	Day–night reversal	0 (0.0)
Apathy	0 (0.0)	Delirium	0 (0.0)
Delirium	0 (0.0)	Depression	0 (0.0)
Disinhibition	0 (0.0)	Disinhibition	0 (0.0)
Dizziness	0 (0.0)	Dysosmia	0 (0.0)
Dysosmia	0 (0.0)	Eating non-edible things	0 (0.0)
Dysphagia	0 (0.0)	Food refusal	0 (0.0)
Eating non-edible things	0 (0.0)	Increase in appetite	0 (0.0)
Food refusal	0 (0.0)	Negativism	0 (0.0)
Increase in appetite	0 (0.0)	Other psychiatric symptoms	0 (0.0)
Negativism	0 (0.0)	Periodic limb movement disorder	0 (0.0)
Periodic limb movement disorder	0 (0.0)	Rest tremor	0 (0.0)
Restless legs syndrome	0 (0.0)	Restless legs syndrome	0 (0.0)
Sudden sleep	0 (0.0)	Sudden sleep	0 (0.0)
Syncope	0 (0.0)	Syncope	0 (0.0)
Unbalanced diet	0 (0.0)	Unbalanced diet	0 (0.0)
Invalid answer	103 (39.2)	Invalid answer	115 (43.7)
Multiple answers	60 (22.8)	Multiple answers	59 (22.4)
Do not know	31 (11.8)	Do not know	41 (15.6)
Unanswered	12 (4.6)	Unanswered	15 (5.7)

Similar trends in frequency were observed for symptoms that cause the patient most distress and the symptoms that the patient would most likely prioritize for receiving treatment. Of the 52 symptoms, 35 (67.3%) were selected as symptoms that cause the patient most distress and 34 (65.4%) were selected as symptoms that the patient would most likely prioritize for receiving treatment.

### Symptoms that cause the caregiver most distress and symptoms that the caregiver would most likely prioritize for receiving treatment

Table 4 shows the survey results of symptoms that cause the caregiver most distress and symptoms that the caregiver would most likely prioritize for receiving treatment. The number of valid answers for “symptom that causes the caregiver most distress” and “as the patient continues with treatment, symptoms that the caregiver would most likely prioritize for receiving treatment” were 183 (69.6%) and 177 (67.3%), respectively. Multiple choice answers were the most common reason for invalid answers. Symptoms that cause the caregiver most distress, in descending order of frequency, were memory impairment (9.5%), visual hallucinations (7.6%), agitation/aggression (5.3%), bradykinesia/akinesia (4.2%), and executive dysfunction (4.2%). The symptoms that the caregiver would most likely prioritize for receiving treatment, in descending order, were memory impairment (9.1%), visual hallucinations (6.1%), bradykinesia/akinesia (5.7%), agitation/aggression (4.2%), and delusions (4.2%).

**Table 4:** Symptoms causing caregivers most distress and symptoms that caregivers would most likely prioritize for treatment

Symptoms that cause the patient most distress (*N =* 263)		Symptoms that the patient would most likely prioritize for receiving treatment (*N =* 263)	
**Symptom domain**	***n*** **(%)**	**Symptom domain**	***n*** **(%)**
Psychiatric symptoms	57 (21.7)	Psychiatric symptoms	54 (20.5)
Cognitive impairment	55 (20.9)	Parkinsonism	46 (17.5)
Parkinsonism	34 (12.9)	Cognitive impairment	42 (16.0)
Autonomic dysfunction	18 (6.8)	Autonomic dysfunction	17 (6.5)
Sleep-related disorders	14 (5.3)	Sleep-related disorders	14 (5.3)
Eating behavior-related problems	5 (1.9)	Eating behavior-related problems	3 (1.1)
Sensory disorders	0 (0.0)	Sensory disorders	1 (0.4)
**Symptom**	***n*** **(%)**	**Symptom**	***n*** **(%)**
Memory impairment	25 (9.5)	Memory impairment	24 (9.1)
Visual hallucinations	20 (7.6)	Visual hallucinations	16 (6.1)
Agitation/aggression	14 (5.3)	Bradykinesia/akinesia	15 (5.7)
Bradykinesia/akinesia	11 (4.2)	Agitation/aggression	11 (4.2)
Executive dysfunction	11 (4.2)	Delusions	11 (4.2)
Delusions	10 (3.8)	Constipation	10 (3.8)
Constipation	8 (3.0)	Postural instability	7 (2.7)
Fluctuating cognition	7 (2.7)	Rapid eye movement sleep behavior disorder	7 (2.7)
Nighttime dysuria	7 (2.7)	Executive dysfunction	6 (2.3)
Other cognitive impairment	6 (2.3)	Gait disturbance	6 (2.3)
Postural instability	6 (2.3)	Other cognitive impairment	5 (1.9)
Rapid eye movement sleep behavior disorder	6 (2.3)	Aberrant motor behavior	4 (1.5)
Gait disturbance	4 (1.5)	Abnormal posture	4 (1.5)
Hallucinations other than visual hallucinations	4 (1.5)	Action tremor	4 (1.5)
Aberrant motor behavior	3 (1.1)	Depression	4 (1.5)
Attention dysfunction	3 (1.1)	Fall	4 (1.5)
Nighttime sleep disorder	3 (1.1)	Rigidity	4 (1.5)
Other psychiatric symptoms	3 (1.1)	Daytime somnolence	3 (1.1)
Rigidity	3 (1.1)	Fluctuating cognition	3 (1.1)
Abnormal posture	2 (0.8)	Hallucinations other than visual hallucinations	3 (1.1)
Action tremor	2 (0.8)	Nighttime dysuria	3 (1.1)
Daytime somnolence	2 (0.8)	Attention dysfunction	2 (0.8)
Depression	2 (0.8)	Nighttime sleep disorder	2 (0.8)
Fall	2 (0.8)	Other psychiatric symptoms	2 (0.8)
Loss of appetite	2 (0.8)	Orthostatic hypotension	2 (0.8)
Restless legs syndrome	2 (0.8)	Restless legs syndrome	2 (0.8)
Salivation	2 (0.8)	Anxiety	1 (0.4)
Visuospatial dysfunction	2 (0.8)	Apathy	1 (0.4)
Weight gain	2 (0.8)	Daytime dysuria	1 (0.4)
Anxiety	1 (0.4)	Delirium	1 (0.4)
Disorientation	1 (0.4)	Disorientation	1 (0.4)
Dizziness	1 (0.4)	Dysosmia	1 (0.4)
Freezing of gait	1 (0.4)	Food refusal	1 (0.4)
Increase in appetite	1 (0.4)	Freezing of gait	1 (0.4)
Orthostatic hypotension	1 (0.4)	Increase in appetite	1 (0.4)
Rest tremor	1 (0.4)	Rest tremor	1 (0.4)
Sudden sleep	1 (0.4)	Syncope	1 (0.4)
Syncope	1 (0.4)	Visuospatial dysfunction	1 (0.4)
Apathy	0 (0.0)	Weight gain	1 (0.4)
Day–night reversal	0 (0.0)	Day–night reversal	0 (0.0)
Daytime dysuria	0 (0.0)	Disinhibition	0 (0.0)
Delirium	0 (0.0)	Disturbance of sweating	0 (0.0)
Disinhibition	0 (0.0)	Dizziness	0 (0.0)
Disturbance of sweating	0 (0.0)	Dysphagia	0 (0.0)
Dysosmia	0 (0.0)	Eating non-edible things	0 (0.0)
Dysphagia	0 (0.0)	Loss of appetite	0 (0.0)
Eating non-edible things	0 (0.0)	Negativism	0 (0.0)
Food refusal	0 (0.0)	Periodic limb movement disorder	0 (0.0)
Negativism	0 (0.0)	Salivation	0 (0.0)
Periodic limb movement disorder	0 (0.0)	Sudden sleep	0 (0.0)
Unbalanced diet	0 (0.0)	Unbalanced diet	0 (0.0)
Weight loss	0 (0.0)	Weight loss	0 (0.0)
Invalid answer	80 (30.4)	Invalid answer	86 (32.7)
Multiple answers	65 (24.7)	Multiple answers	81 (30.8)
Do not know	10 (3.8)	Do not know	4 (1.5)
Unanswered	5 (1.9)	Unanswered	1 (0.4)

Similar trends in frequency were observed for symptoms that cause the caregiver most distress and symptoms that the caregiver would most likely prioritize for receiving treatment. Of the 52 symptoms, 38 (73.0%) were selected as symptoms that cause the caregiver most distress and 39 (75.0%) were selected as the symptoms that the caregiver would most likely prioritize for receiving treatment.

Symptoms (multiple answers allowed) that cause the patients (questionnaire for patients Q19) and caregivers (questionnaire for caregivers Q40) distress are shown in Supplementary Table 2 (Additional file 7).

### Attending physicians’ understanding of the symptom domain that causes patients and caregivers most distress

In terms of pre-defined domains of symptoms that cause the patients most distress and symptoms they would most likely prioritize for receiving treatment, parkinsonism was the most frequent, followed by cognitive impairment and autonomic dysfunction for both questions (Table 3). However, psychiatric symptoms were the most frequent domain for both symptoms that cause the caregivers most distress and symptoms they would most likely prioritize for receiving treatment. For symptom domains that cause the caregivers most distress, psychiatric symptoms were followed by cognitive impairment and parkinsonism. Regarding the symptom domains that the caregivers would most likely prioritize for receiving treatment, psychiatric symptoms were followed by parkinsonism and cognitive impairment (Table 4).

**Fig. 2:**
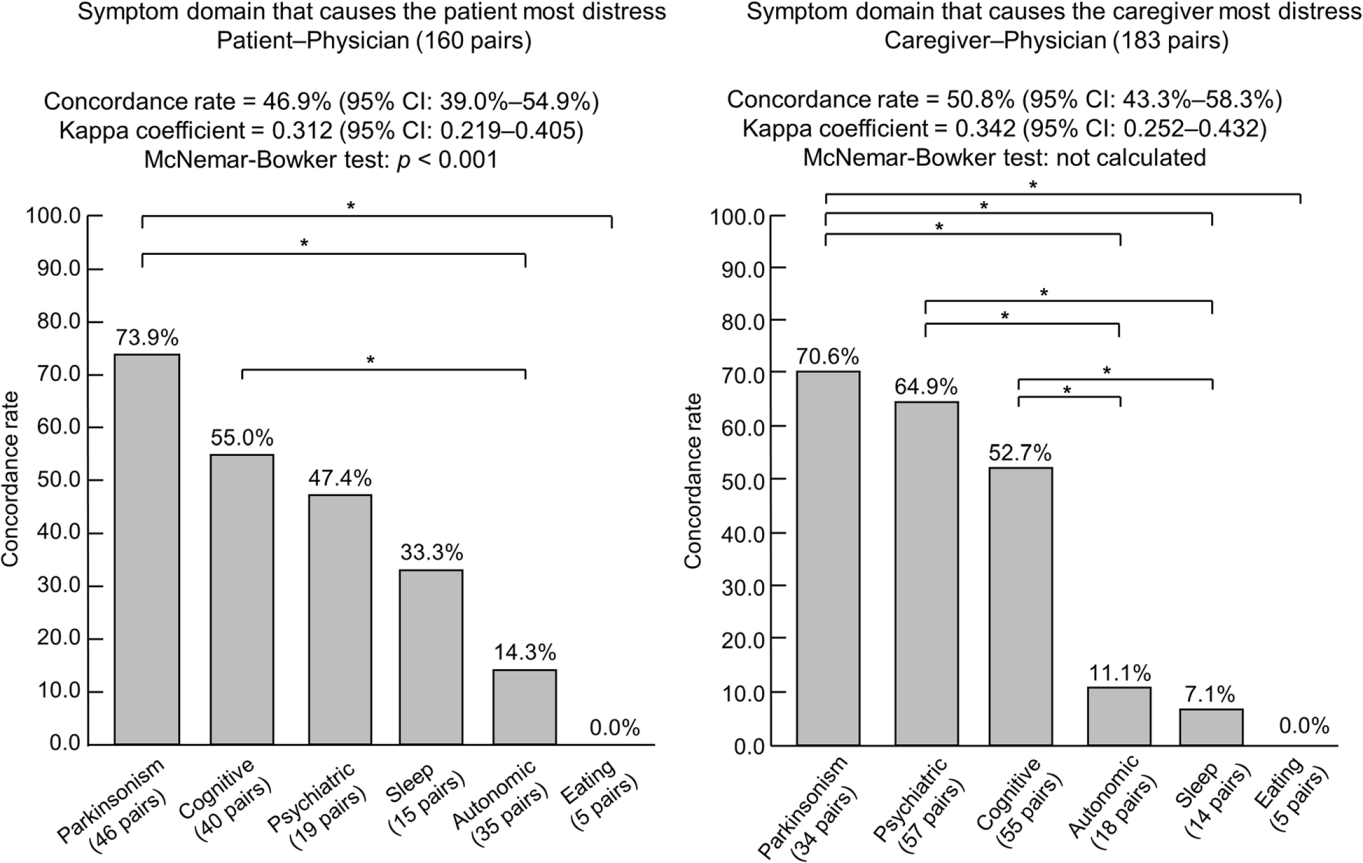
Comparison of patient–physician and caregiver–physician concordance rates of each domain. *p < 0.05 Fisher’s exact test. *Abbreviations: Autonomic* autonomic dysfunction, *CI* confidence interval, *Cognitive* cognitive impairment, *Eating* eating behavior-related problems, *Psychiatric* psychiatric symptoms, *Sleep* sleep-related disorders

The patient–physician concordance rates of all symptom domains for “domain that causes the patient most distress,” and caregiver–physician concordance rates for the symptom domain that causes the caregiver most distress” were 46.9% (95% CI: 39.0–54.9%, kappa coefficient = 0.312 [95% CI: 0.219–0.405], McNemar–Bowker test: *p* < 0.001) and 50.8% (95% CI: 43.3–58.3%, kappa coefficient = 0.342 [95% CI: 0.252–0.432], McNemar–Bowker test: not calculated), respectively (Fig. 2).

The concordance rate for symptom causing the patient most distress was similar regardless of the severity of cognitive impairment based on the MMSE (MMSE-J ≥20, 47.4%; MMSE ≤19, 45.5%; *p* = 0.861).

Suplementary Fig. 1 (Additional file 8) shows 7 × 7 tables with the number of answers selected by patients and physicians and by caregivers and physicians for the symptom domain that causes the patient/caregiver most distress.

### Assessment of difference in whether patients answered themselves or with assistance by their caregiver

The patient–caregiver concordance rate of all symptom domains for “domain that causes the patient most distress according to the patient” was 65.7% (95% CI: 57.0–73.7%, kappa coefficient = 0.558 [95% CI: 0.456–0.660], McNemar–Bowker test: *p* = 0.775). There was no significant difference in the patient–caregiver concordance rates irrespective of who filled out the questionnaire: 70.8% when the patients themselves filled out the questionnaire and 59.7% when the caregivers listened to the answer and filled out the questionnaire.

### Comparison of concordance rates by symptom domain

Figure 2 shows the results of the comparison of the concordance rates for each symptom domain that patients or caregivers selected that causes them the most distress, except for sensory disorders, for which there were no answers. Regarding the symptom domain that causes the patient most distress, the domain with the highest concordance rate was parkinsonism, followed by cognitive impairment and psychiatric symptoms. The patient–physician concordance rate for parkinsonism was significantly higher than for autonomic dysfunction and eating behavior-related problems. The patient–physician concordance rate for cognitive impairment was significantly higher than for autonomic dysfunction. Regarding the symptom domain that causes the caregiver most distress, the domain with the highest concordance rate was parkinsonism, followed by psychiatric symptoms and cognitive impairment. The caregiver–physician concordance rates for parkinsonism, psychiatric symptoms, and cognitive impairment were significantly higher than for autonomic dysfunction and sleep-related disorders.

### Search for factors using the stepwise method

The results of the multivariate analysis using the stepwise method also explored the factors contributing to the rise in discordance between patients and physicians for the symptom domain that causes the patients most distress. Using “parkinsonism” as a reference, “cognitive impairment” (odds ratio [OR] 3.72; *p* = 0.018), “psychiatric symptoms” (OR 5.10; *p* = 0.018), “sleep-related disorders” (OR 10.88; *p* = 0.003), and “autonomic dysfunction” (OR 28.53; *p* < 0.001) were more likely to contribute to the rise in discordance between patients and physicians for the symptom domain that causes the patient most distress. Regarding the amount of time the caregiver spent with the patient, <16 h/day had an OR of 3.679 (*p* = 0.003) compared with ≥16 h/day, a factor that increased the discordance rate (Table 5 and Supplementary Table 3, Additional file 9).

**Table 5:** Symptom domain causing patients most distress: matching status and regression analysis for patient–physician discordance

Between patient and physician	Symptom domain that causes the patient most distress		Univariate LR analysis			Multivariate LR: input significant variables with variable increasing method (likelihood ratio)		
	Concordance (*n* = 75)	Discordance (*n* = 85)	OR	95% CI	*p* value	OR	95% CI	*p* value
**Patient-side factors**								
Patient’s age (y)						n.e.		
<80.0	43 (57.3)	35 (41.2)	1.000	ref				
≥80.0	32 (42.7)	50 (58.8)	1.920	1.023–3.602	0.042			
Number of persons living with the patient								
Alone	4 (5.3)	14 (16.5)	1.000	ref		n.e.		
Two	35 (46.7)	36 (42.4)	0.294	0.088–0.980	0.046			
Three or more	36 (48.0)	35 (41.2)	0.278	0.083–0.927	0.037			
Patient’s initial symptom domain						n.e.		
Cognitive impairment	19 (25.7)	35 (41.2)	5.066	1.895–13.541	0.001			
Parkinsonism	22 (29.7)	8 (9.4)	1.000	ref				
Psychiatric symptoms	15 (20.3)	24 (28.2)	4.400	1.563–12.385	0.005			
Eating behavior-related problems	0 (0.0)	1 (1.2)	n.c.					
Sleep-related disorders	16 (21.6)	10 (11.8)	1.719	0.555–5.326	0.348			
Autonomic dysfunctions	2 (2.7)	3 (3.5)	4.125	0.579–29.392	0.157			
Sensory disorders	0 (0.0)	4 (4.7)	n.c.					
MDS-UPDRS Part III total score						n.e.		
<18.0	27 (36.0)	46 (54.8)	1.000	ref				
≥18.0	48 (64.0)	38 (45.2)	0.465	0.246–0.879	0.019			
MDS-UPDRS Part II total score						n.e.		
<9.0	30 (40.0)	48 (57.1)	1.000	ref				
≥9.0	45 (60.0)	36 (42.9)	0.500	0.266–0.941	0.032			
Pharmacotherapy for cognitive impairment						n.e.		
None	23 (30.7)	13 (15.3)	1.000	ref				
Yes	52 (69.3)	72 (84.7)	2.450	1.137–5.280	0.022			
Pharmacotherapy for parkinsonism						n.e.		
None	35 (46.7)	55 (64.7)	1.000	ref				
Yes	40 (53.3)	30 (35.3)	0.477	0.253–0.901	0.023			
Most inconvenient domain that patients selected								
Cognitive impairment	22 (29.3)	18 (21.2)	2.318	0.937–5.737	0.069	3.720	1.256–11.019	0.018
Parkinsonism	34 (45.3)	12 (14.1)	1.000	ref		1.000	ref	
Psychiatric symptoms	9 (12.0)	10 (11.8)	3.148	1.032–9.604	0.044	5.101	1.328–19.601	0.018
Eating behavior-related problems	0 (0.0)	5 (5.9)	n.c.			n.c.		
Sleep-related disorders	5 (6.7)	10 (11.8)	5.667	1.609–19.961	0.007	10.884	2.286–51.814	0.003
Autonomic dysfunction	5 (6.7)	30 (35.3)	17.000	5.367–53.851	<0.001	28.533	6.419–126.830	<0.001
Sensory disorders	0 (0.0)	0 (0.0)	-			-		
Whether the patient told their caregiver or family members about patient’s most inconvenient symptom						n.e.		
Yes	63 (91.3)	64 (78.0)	1.000	ref				
No	4 (5.8)	16 (19.5)	3.937	1.247–12.430	0.019			
Unknown	2 (2.9)	2 (2.4)	0.984	0.984–0.134	0.988			
**Caregiver-side factors**								
Relationship with the patient from patient’s perspective						n.e.		
Spouse	43 (57.3)	33 (38.8)	1.000	ref				
Non-spouse	32 (42.7)	52 (61.2)	2.117	1.125–3.984	0.020			
Time spent with the patient (h/day)								
<16.0	27 (36.5)	49 (59.0)	2.509	1.317–4.779	0.005	3.679	1.538–8.799	0.003
≥16.0	47 (63.5)	34 (41.0)	1.000	ref		1.000	ref	
Patient’s most inconvenient symptom domain that the caregiver selected						n.e.		
Cognitive impairment	19 (31.1)	18 (24.7)	1.759	0.704–4.394	0.226			
Parkinsonism	26 (42.6)	14 (19.2)	1.000	ref				
Psychiatric symptoms	8 (13.1)	11 (15.1)	2.554	0.834–7.816	0.100			
Eating behavior-related problems	0 (0.0)	3 (4.1)	n.c.					
Sleep-related disorders	1 (1.6)	9 (12.3)	16.714	1.917–145.766	0.011			
Autonomic dysfunctions	7 (11.5)	18 24.7()	4.776	1.608–14.179	0.005			
Sensory disorders	0 (0.0)	0 (0.0)	-					

*Abbreviations*: *CI* confidence interval, *LR* logistic regression, *MDS-UPDRS* Movement Disorder Society-Unified Parkinson’s Disease Rating Scale, *n.c.* not calculated, *n.e.* not evaluable, *OR* odds ratio, *ref* reference

Regarding the factors contributing to the rise in discordance between caregivers and physicians for the symptom domain that causes the caregiver most distress, using “parkinsonism” as the reference, “sleep-related disorders” and “autonomic dysfunction” were 31.20 times (*p* = 0.002) and 19.20 times (*p* < 0.001) more likely, respectively, to contribute to the rise in discordance between patients and physicians for the symptom domain that causes the caregiver most distress (Table 6 and Supplementary Table 4, Additional file 10).

**Table 6:** Symptom domain causing caregivers most distress: matching status and regression analysis for caregiver–physician discordance

Between caregiver and physician	Symptom domain that causes the caregiver most distress		Univariate LR analysis			Multivariate LR: input significant variables with variable increasing method (likelihood ratio)		
	Concordance (*n* = 93)	Discordance (*n* = 90)	OR	95% CI	*p* value	OR	95% CI	*p* value
**Patient-side factor**								
Autonomic dysfunction						n.e.		
None	63 (67.7)	45 (50.0)	1.000	ref				
Yes	30 (32.3)	45 (50.0)	2.100	1.153–3.826	0.015			
**Caregiver-side factors**								
Patient’s most inconvenient symptom domain that the caregiver selected								
Cognitive impairment	29 (31.2)	26 (28.9)	2.152	0.868–5.335	0.098	2.152	0.868–5.335	0.098
Parkinsonism	24 (25.8)	10 (11.1)	1.000	ref		1.000	ref	
Psychiatric symptoms	37 (39.8)	20 (22.2)	1.297	0.519–3.244	0.578	1.297	0.519–3.244	0.578
Eating behavior-related problems	0 (0.0)	5 (5.6)	n.c.			n.c.		
Sleep-related disorders	1 (1.1)	13 (14.4)	31.200	3.585–271.515	0.002	31.200	3.585–271.515	0.002
Autonomic dysfunction	2 (2.2)	16 (17.8)	19.200	3.707–99.445	<0.001	19.200	3.707–99.445	<0.001
Sensory disorders	0 (0.0)	0 (0.0)	—					
**Physician-side factors**								
Institute						n.e.		
University hospital	48 (51.6)	32 (35.6)	1.000	ref				
Non-university hospital	14 (15.1)	21 (23.3)	2.250	1.000–5.062	0.050			
Clinic	31 (33.3)	37 (41.1)	1.790	0.931–3.443	0.081			

*Abbreviations*: *CI* confidence interval, *LR* logistic regression, *n.c.* not calculated, *n.e.* not evaluable, *OR* odds ratio, *ref* reference

## Discussion

In this study, two questions “which symptoms cause the patient most distress?” and “which symptoms would the patient most likely prioritize for receiving treatment?” were used to determine the treatment needs of patients with DLB and their caregivers. The results of the present study showed that the symptoms that cause the patient most distress and the symptoms that the patient would most likely prioritize for receiving treatment were very similar; these included memory impairment, constipation, bradykinesia/akinesia, visual hallucinations, and nighttime sleep disorder. The symptoms that cause the caregiver most distress and the symptoms that the caregiver would most likely prioritize for receiving treatment were also very similar; these included memory impairment, visual hallucinations, agitation/aggression, and bradykinesia/akinesia. In this study, we surveyed the symptom causing participants most distress, and for this question, the patients with DLB and their caregivers, respectively, selected 35 (67.3%) symptoms and 38 (73.0%) symptoms of the 52 types of symptoms pre-selected. These results indicate that the clinical symptoms of DLB are varied and the treatment needs of patients/caregivers are likewise very diverse.

A distinctive feature of this study is that the treatment needs of patients with DLB from their own perspectives were investigated. In recent years, dementia care that emphasizes patients’ wishes has been promoted mainly in Europe and the USA [31]. However, to date, few studies have examined the needs of patients with DLB from their own perspectives. A reason for this may be the assumption that patients with DLB would not be able to give correct answers because of their impaired judgment, poor communication skills, and lower level of consciousness of their disease caused by dementia. The present study allowed caregivers to listen to patients’ answers and fill out a questionnaire for the patient. It is possible that these answers from patients reflect the caregivers’ wishes and not the real needs of the patient. However, we found no difference in whether patients answered themselves or with assistance by their caregiver, suggesting that the assistance provided by the caregivers when filling out the questionnaire for the patient had little impact on the patients’ answers. Of note, in the present study, there was an issue regarding the reliability of the answers because a considerable proportion of patients selected more than one symptom when requested to choose only one symptom that currently causes them most distress. However, approximately 60% of the patients were able to answer the questionnaire correctly, and there was no significant difference in the clinical evaluation of the patients, including cognitive function, between those who answered correctly and those who answered incorrectly (data not shown). Therefore, our findings do reflect the treatment needs of patients with DLB and are very valuable for physicians who treat patients with DLB.

Regarding the treatment needs of patients themselves for both symptom that causes them most distress and symptom they would most likely prioritize for receiving treatment, memory impairment was the most frequently selected. Some studies have described attention dysfunction, executive dysfunction, or visuospatial dysfunction as the main characteristics of cognitive impairment in DLB and that memory impairment is relatively mild [32–34]. Despite efforts made to address this point (the movie provided and table with symptom definitions), it is possible that patients and caregivers may have conflated memory impairment with cognitive impairment such as attention dysfunction or executive function, which cannot be ruled out. However, memory impairment has been reported to be a frequent symptom in the early stages of DLB [35]. Although memory impairment may have been overestimated, the results of this study reiterate the importance of physicians paying attention to patients’ memory impairment in clinical settings.

Constipation was the second most common symptom selected by 20 patients as that causing them most distress. It has been reported that constipation is frequent and often precedes cognitive impairment in patients with DLB [36], and it decreases ADL and quality of life in patients with Parkinson’s disease [37]. Bradykinesia/akinesia was the third or second most common symptom selected by patients as that causing them most distress or as that they would most likely prioritize for receiving treatment, respectively. It has been reported that in DLB, bradykinesia and gait disturbance are more frequent than tremor at rest [38]. McKeith et al. reported that parkinsonism, including bradykinesia/akinesia, was associated with ADL impairment [39]. We speculate that patients were more distressed by constipation and bradykinesia/akinesia because these symptoms more frequently occurred and are likely to affect ADL and quality of life. However, these results could be interpreted as indicating that physical symptoms such as parkinsonism and autonomic dysfunction were more easily perceived than psychiatric symptoms, such as visual hallucinations and delusions, and thus more likely to cause distress. In fact, Ballard et al. reported that 63% of patients with DLB lacked consciousness of their disease for visual hallucinations [40], and it is necessary to understand that the treatment needs of patients themselves may reflect the patient’s awareness of his or her illness.

For the caregivers, memory impairment was the most frequently selected symptom that causes them most distress and symptom they would most likely prioritize for receiving treatment, which was the same as in the patients. The second most common symptom that causes the caregiver most distress was visual hallucinations. However, psychiatric symptoms were the most frequently selected symptom domain for the symptom that causes the caregiver most distress. This result supports the results of a previous study that found that BPSD is the most burdensome DLB symptom for caregivers [19].

This study determined to what extent attending physicians understand the various treatment needs of patients with DLB and their caregivers. In the treatment of patients with dementia, the attending physician should usually make treatment decisions based on the patient’s or caregiver’s wishes. Therefore, the attending physician needs to understand the symptoms that cause the patient or caregiver most distress. From this perspective, it is desirable that the patient–physician and caregiver–physician concordance rates be as close to 100% as possible. However, the observed concordance rate was approximately 50% for symptom domains that cause the patient or caregiver most distress, and the calculated kappa coefficients showed poor concordance. The physicians in this study could be considered specialists in DLB, and indeed, more than half of the physicians had experience treating more than 100 patients with DLB. Nevertheless, the present results, in which only half of the pairs of attending physicians were able to adequately identify the most troubling symptom categories of their patients and caregivers, indicate that it is extremely difficult to identify the treatment needs of patients with DLB and their caregivers. The symmetry assessment showed statistically significant differences in the patient–physician concordance rate for the symptom domain that causes the patient most distress. This result suggests that the physicians selected cognitive impairment or parkinsonism in cases in which their patients selected autonomic dysfunction.

To search for factors that make it difficult for attending physicians to identify the treatment needs of patients and caregivers, we performed a logistic regression analysis. Using all possible factors, such as lack of communication between patient and physician (whether the patient’s physician listens to what the patient says), less time for consultation (frequency of visits), and patient’s or caregiver’s level of understanding as explanatory variables, only “symptom domain that causes the patient most distress” and “time the caregiver spends with the patient each day” were statistically significant. Patient–physician concordance rates were significantly lower in cases in which the patients selected autonomic dysfunction, or sleep-related disorders compared with cases in which the patient selected parkinsonism or cognitive impairment as the symptom that causes the patient most distress. Similar results were shown in the caregiver–physician concordance rates. These results suggest that physicians tend to focus on cognitive impairment, parkinsonism, and psychiatric symptoms and tend to focus less on autonomic dysfunction, sleep-related disorders, and eating behavior-related problems, among the various symptoms of DLB. Although autonomic dysfunction was the third most common symptom domain selected by the patients as that which causes them most distress, it was not a symptom of concern for the physicians. This may be because it is positioned as a supportive clinical feature rather than a core clinical feature in the DLB clinical diagnostic criteria.

## Limitations

The present study has some limitations, such as those inherent to questionnaire surveys. First, prior to commencing the study, a literature search was conducted but an article that discussed all the clinical manifestations of DLB could not be found; therefore, 52 symptoms that were considered to be frequent and clinically important for patients with DLB were selected based on our internal discussion. It is possible that some important clinical symptoms may have been inadvertently omitted from this study. Second, although most patients had multiple symptoms in the present study, the present study required the patients and caregivers to select only one symptom that causes them the most distress. This process was difficult for patients and caregivers, and in fact, the number of those multiple choice answers was over 20%. In addition, physicians may have chosen the second most distress symptom. These may have resulted in a low concordance rate. Third, despite efforts made to address this point (the movie provided and table with symptom definitions), in particular, there was an issue with the reliability of the patients’ answers. The concordance rate for the symptom causing the patient most distress was similar regardless of the severity of cognitive impairment based on the MMSE. Furthermore, caregivers were allowed to assist the patients in answering the questionnaire, but the possibility of inaccuracies in the patients’ answers due to their cognitive impairment must be considered. Fourth, most of the patients in this study were residing at home rather than in residential facilities (e.g., nursing homes) and their mean total MMSE-J score was 20.9, suggesting that many of them had relatively mild DLB. Therefore, the results of this study may not be applicable to all patients with DLB but rather those with mild DLB. Fifth, in this study, the concordance rate of symptom domains was used as an indicator for the attending physician’s understanding of the treatment needs of the patients and caregivers. However, considering that even within the same symptom domain, treatment methods for “hallucinations and depression” and “constipation and frequent urination” are different, it may have been desirable to use concordance rates for all symptoms. Therefore, the true concordance rate may have been lower than the results presented in this study. Finally, this study was conducted under conditions of increased anxiety and concerns about various social conditions among patients and their caregivers due to the COVID-19 pandemic. Therefore, the findings may differ from those obtained in normal conditions.

## Conclusions

In conclusion, this study investigated the treatment needs of patients with DLB and their caregivers using a questionnaire. The most frequent symptom causing distress among patients was memory impairment, followed by constipation and bradykinesia. Among caregivers, memory impairment was also the most frequently selected symptom causing distress, followed by visual hallucinations and agitation/aggression. Thirty-five symptoms were selected as those causing the patients most distress and 38 symptoms were selected as those causing the caregivers most distress, indicating that there is some variability in the treatment needs of patients with DLB and their caregivers. Attending physicians had difficulty understanding the top treatment needs of their patients and caregivers, despite their expertise in DLB. Attending physicians should pay more attention to autonomic dysfunction and sleep-related disorders in the treatment of DLB.

## Supplementary Information

**Additional file 1: Supplementary Table 1.** Definitions for the 52 symptoms.

**Additional file 2: Supplementary Methods 1.** Questionnaire for patient.

**Additional file 3: Supplementary Methods 2.** Questionnaire for caregiver.

**Additional file 4: Supplementary Methods 3.** Questionnaire for physician: Part 1.

**Additional file 5: Supplementary Methods 4.** Questionnaire for physician: Part 2.

**Additional file 6: Supplementary Methods 5.** Other indicators assessed for concordance rate.

**Additional file 7: Supplementary Table 2.** Symptoms that cause the patient or caregiver distress.

**Additional file 8: Supplementary Fig. 1.** Number of answers selected by patients and physicians and by caregivers and physicians for “symptom domain that causes the patient most distress according to the patient/caregiver”. The gray cells indicate the symptom domains that are consistent between the two groups. *Abbreviations*: *Aut* autonomic dysfunction, *Cog* cognitive impairment, *Eat* eating behavior-related problems, *Par* parkinsonism, *Psy* psychiatric symptoms, *Sen* sensory disorders, *Sle* sleep-related disorders.

**Additional file 9: Supplementary Table 3.** Symptom domain causing patients most distress: matching status and regression analysis for patient–physician discordance.

**Additional file 10: Supplementary Table 4.** Symptom domain causing caregivers most distress: matching status and regression analysis for caregiver–physician discordance.

**Additional file 11: Supplementary information about coinvestigators.** List of coinvestigators.

## Data Availability

Owing to participant privacy, individual-level data cannot be made publicly available.

## Abbreviations

AD: Alzheimer’s disease
ADL: Activities of daily living
BPSD: Behavioral and psychological symptoms of dementia
CFI: Cognitive Fluctuation Inventory
CI: Confidence interval
DLB: Dementia with Lewy bodies
J-ZBI_8: Shortened Japanese version of the Zarit Caregiver Burden Interview
MDS-UPDRS: Movement Disorder Society-Unified Parkinson’s Disease Rating Scale
MMSE-J: Japanese version of the Mini-Mental State Examination
NPI-12: Neuropsychiatric Inventory-12
OR: Odds ratio
SF-8: Short Form-8
