# Correction: Treatment needs of dementia with Lewy bodies according to patients, caregivers, and physicians: a cross-sectional, observational, questionnaire-based study in Japan

**DOI:** 10.1186/s13195-022-01155-9

**Published:** 2023-01-05

**Authors:** Mamoru Hashimoto, Yuta Manabe, Takuhiro Yamaguchi, Shunji Toya, Manabu Ikeda

**Affiliations:** 1 Department of Psychiatry, Osaka University Graduate School of Medicine, Osaka, Japan; 2 Department of Neuropsychiatry, Kindai University Faculty of Medicine, Osakasayama, Japan; 3 Department of Dementia and Geriatric Medicine, Division of Clinical Science, Kanagawa Dental University School of Dentistry, Yokosuka, Japan; 4 Division of Biostatistics, Tohoku University Graduate School of Medicine, Sendai, Japan; 5 Medical Affairs, Sumitomo Pharma Co., Ltd., Tokyo, Japan

**Correction: Alz Res Therapy 14, 188 (2022)**

**https://doi.org/10.1186/s13195-022-01130-4**

Following publication of the original article [1], the authors corrected “the patient” to “the caregiver” in Table 4 header.

The original article [1] has been updated.
