# Supplementary material for: Treatment needs of dementia with Lewy bodies according to patients, caregivers, and physicians: a cross-sectional, observational, questionnaire-based study in Japan
Source: Alzheimers Res Ther. 2022 Dec 15;14:188. doi: 10.1186/s13195-022-01130-4 (PMC9751509; doi:10.1186/s13195-022-01130-4)
Supplement: Supplementary file 1 — Additional file 1: Supplementary Table 1. Definitions for the 52 symptoms. [file 13195_2022_1130_MOESM1_ESM.docx]

**Supplementary Table 1 Definitions for the 52 symptoms**

|  | **Symptom** | **Explanation** |
| --- | --- | --- |
| 1 | Memory impairment | A state in which one’s memory becomes extremely bad or one cannot recall things even with a hint. |
| 2 | Disorientation | A state in which one cannot tell what time of day it is or where one is. |
| 3 | Executive dysfunction | A state in which planning ahead to do something or to act in accordance with a procedure becomes difficult. |
| 4 | Attention dysfunction | A state in which one feels distracted or one’s attention or concentration is lost. |
| 5 | Fluctuating cognition | A state in which the cognitive function level varies from good to bad (stupor) and response levels fluctuate between lucid to reduced alertness. |
| 6 | Visuospatial dysfunction | A state in which one is unable to find something that is in front of them or is unable to accurately recognize something they see. |
| 7 | Other cognitive impairment | Other symptoms such as difficulty in speaking words (aphasia), difficulty in using items (apraxia), and telling far-fetched stories (fabrication). |
| 8 | Bradykinesia/Akinesia | A state in which it is difficult to move the body or move quickly. |
| 9 | Rigidity | A state in which relaxing the limbs or body is difficult, indicating that the muscles are always tense and stiff. |
| 10 | Action tremor | A state in which one’s hands shake when holding or writing something or one’s legs shake when sitting cross-legged. |
| 11 | Rest tremor | A state in which one’s hands and legs shake while resting relaxed, unlike when shaking occurs when trying to hold or write something. |
| 12 | Postural instability | A state in which keeping one’s balance is difficult and one feels like falling over. |
| 13 | Gait disturbance  (short-stepped gait) | A state in which taking the first step is difficult or one shuffles or takes small steps. |
| 14 | Freezing of gait | A state in which taking the first step is difficult or one stands rooted to the spot and is unable to take the first step. |
| 15 | Abnormal posture | A state in which one’s neck is lowered or one has a hunchback or forward-leaning posture. |
| 16 | Salivation | A state in which one is unable to swallow saliva and drools. |
| 17 | Fall | A state in which one often falls over on a flat road or stairs or falls off a chair from a sitting position. |
| 18 | Dysphagia | A state in which one has difficulty swallowing food and chokes or spits it out. |
| 19 | Delusions | A state in which one falsely believes that their spouse is an imposter or is having an affair or that something was stolen. |
| 20 | Visual hallucinations | A state in which one sees something that does not really exist (person, animal, etc.). |
| 21 | Hallucinations  other than visual hallucinations | A state in which one hears the voice of someone who is not present (auditory hallucination) or feels an illusory sensation and says something like “An insect is crawling under my skin” (cenesthesic hallucination). |
| 22 | Agitation/Aggression | A state in which one has a heightened sense of emotion, speaks violent words, or resorts to violence. |
| 23 | Depression | A state in which one is depressed and has no motivation. |
| 24 | Anxiety | A state in which one feels restless or fidgety. |
| 25 | Apathy | A state in which one has lost interest in one’s surroundings and is unable to take voluntary action. |
| 26 | Disinhibition | A state in which one is unable to suppress one’s emotions or desires and uses speech and actions that are not acceptable in society. |
| 27 | Aberrant motor behavior | A state in which one engages in unusual behavior (e.g., wanders around or checks something excessively). |
| 28 | Negativism | A state in which one refuses everything that is offered, including going to day service (refusal to eat should be classified as anorexia). |
| 29 | Delirium | A state in which one’s psychological state deteriorates owing to being in an environment that is different from normal, such as being in a hospital or being ill. |
| 30 | Other psychiatric symptoms | Other symptoms such as being excessively dependent on caregivers (dependence) or excessively persistent (obsession). |
| 31 | Loss of appetite | A state in which one has little or no desire to eat. |
| 32 | Increase in appetite | A state in which one has a heightened desire to eat. |
| 33 | Weight loss | A state in which one loses weight. |
| 34 | Weight gain | A state in which one gains weight. |
| 35 | Food refusal | A state in which one says no to meals or refuses to eat even when being encouraged to eat. |
| 36 | Eating non-edible things | A state in which one eats something that is not food. |
| 37 | Unbalanced diet | A state in which one is very picky about meals. |
| 38 | Rapid eye movement sleep behavior disorder | A state of sleep (dreaming) in which one talks in long sentences or talks as if in a conversation, yells, or does things like kicking and punching. |
| 39 | Daytime somnolence | A state in which one falls asleep during the daytime even after a good night’s sleep. |
| 40 | Day-night reversal | A state in which one stays awake during the night and sleeps during the day. |
| 41 | Nighttime sleep disorder | A state in which one is unable to sleep well, wakes up many times at night, and wakes up early in the morning. |
| 42 | Sudden sleep | A state in which one suddenly falls asleep despite having been awake until that time. |
| 43 | Restless legs syndrome | A state in which one feels restless in the legs while sitting or lying down. |
| 44 | Periodic limb movement disorder | A state in which one or both arms or legs involuntarily move periodically and repeatedly at a set interval. |
| 45 | Orthostatic hypotension | A state in which one feels like they are losing color or fainting, goes pale, or becomes dizzy when standing up from sitting or lying down. |
| 46 | Disturbance of  sweating | A state in which one sweats heavily only on the upper body or sweats only on the upper body despite feeling cold. |
| 47 | Constipation | A state in which one has poor bowel movements, which are sometimes accompanied by pain. |
| 48 | Nighttime dysuria | Frequent urination (one gets up to go to the toilet three or more times during sleep), urinary incontinence, and a sensation of residual urine (one feels the urge to urinate even after urination). |
| 49 | Daytime dysuria | Frequent urination (one urinates eight or more times during the day), urinary incontinence, and a sensation of residual urine (one feels the urge to urinate even after urination). |
| 50 | Syncope | A state in which one experiences a temporary loss of consciousness but recovers after a few minutes (often observed after a meal or going to the toilet). |
| 51 | Dizziness | Dizziness is a term used to describe a range of sensations, such as feeling faint, woozy, weak, or unsteady. |
| 52 | Dysosmia | Dysosmia is a disorder described as any qualitative alteration or distortion of the perception of smell. |
