# Supplementary material for: Treatment needs of dementia with Lewy bodies according to patients, caregivers, and physicians: a cross-sectional, observational, questionnaire-based study in Japan
Source: Alzheimers Res Ther. 2022 Dec 15;14:188. doi: 10.1186/s13195-022-01130-4 (PMC9751509; doi:10.1186/s13195-022-01130-4)
Supplement: Supplementary file 2 — Additional file 2: Supplementary Methods 1. Questionnaire for patient. [file 13195_2022_1130_MOESM2_ESM.docx]

| **Questionnaire for Patient**  Research on the Treatment Needs of Patients with  Dementia with Lewy Bodies, their Caregivers, and their Physicians |
| --- |

| **Date of Completion of Questionnaire: Y M D**  ***Please fill in the date.** |
| --- |

- Please complete this questionnaire at the earliest (within 3 weeks) from the day on which it was distributed.
- Approximately 15–20 min would be required to complete this questionnaire.

(The time required may vary slightly depending on the content of the responses.)

Please bear in mind the following when filling in the questionnaire:

- Please circle your response for each question. Please write a detailed answer in the space provided for questions where you are asked to make a written response.
- Your caregiver may ask you the questions and fill in the answers for you. However, in such a case, your caregiver must first fill in their own questionnaire before assisting you with your questionnaire. If your caregiver fills in the questionnaire for you, please do not allow your answers to be influenced by your caregiver.
- After completing, please check that you have not missed any questions.
- **After checking, please submit the questionnaire to your caregiver**.

| **Please tick if your caregiver listened to your opinions and completed the questionnaire for you.** | ⇒ | **□　Yes** |
| --- | --- | --- |

| Questionnaire Help Desk  TEL：0120-549-715  Available from 10:00 to 18:00 Monday to Friday  ＊Not available on Saturdays, Sundays or public holidays. |
| --- |

| For Company Use Only | Identification Code: ××-×× |
| --- | --- |

Symptoms of Dementia with Lewy Bodies (DLB)

|  | **Symptom domain** | **Symptom** | **Explanation** |
| --- | --- | --- | --- |
| 1 | Cognitive impairment | Memory impairment | A state in which one’s memory becomes extremely bad or one cannot recall things even with a hint. |
| 2 |  | Disorientation | A state in which one cannot tell what time of day it is or where they are. |
| 3 |  | Executive dysfunction | A state in which planning ahead to do something or to act in accordance with a procedure becomes difficult. |
| 4 |  | Attention dysfunction | A state in which one feels distracted or one’s attention or concentration is lost. |
| 5 |  | Fluctuating cognition | A state in which the cognitive function level varies from good to bad (stupor) and response levels fluctuate between lucid to reduced alertness. |
| 6 |  | Visuospatial dysfunction | A state in which one is unable to find something that is in front of them or is unable to accurately recognize something they see. |
| 7 |  | Other cognitive impairment | Other symptoms such as difficulty in speaking words (aphasia), difficulty in using items (apraxia), telling far-fetched stories (fabrication). |
| 8 | Parkinsonism | Bradykinesia/  Akinesia | A state in which it is difficult to move the body or move quickly. |
| 9 |  | Rigidity | A state in which relaxing the limbs or body is difficult, indicating that the muscles are always tense and stiff. |
| 10 |  | Action tremor | A state in which one’s hands shake when holding or writing something or one’s legs shake when sitting cross-legged. |
| 11 |  | Rest tremor | A state in which one’s hands and legs shake while resting relaxed, unlike when shaking occurs when trying to hold or write something. |
| 12 |  | Postural instability | A state in which keeping one’s balance is difficult and one feels like falling over. |
| 13 |  | Gait disturbance (short-stepped gait) | A state in which taking the first step is difficult or one shuffles or takes small steps. |
| 14 |  | Freezing of gait | A state in which taking the first step is difficult or one stands rooted to the spot and is unable to take the first step. |
| 15 |  | Abnormal posture | A state in which one’s neck is lowered or has a hunchback or forward-leaning posture. |
| 16 |  | Salivation | A state in which one is unable to swallow saliva and drools. |
| 17 |  | Fall | A state in which one often falls over on a flat road or stairs or falls off a chair from a sitting position. |
| 18 |  | Dysphagia | A state in which one has difficulty swallowing food and chokes or spits it out. |
| 19 | Psychiatric symptoms | Delusions | A state in which one falsely believes that their spouse is an imposter or is having an affair or that something was stolen. |
| 20 |  | Visual hallucinations | A state in which one sees something that does not really exist (person, animal, etc.). |
| 21 |  | Hallucinations other than visual hallucinations | A state in which one hears the voice of someone who is not present (auditory hallucination) or feels an illusory sensation and says something like “An insect is crawling under my skin” (cenesthesic hallucination). |
| 22 |  | Agitation/  Aggression | A state in which one has a heightened sense of emotion, speaks violent words, or resorts to violence. |
| 23 |  | Depression | A state in which one is depressed and has no motivation. |
| 24 |  | Anxiety | A state in which one feels restless or fidgety. |
| 25 |  | Apathy | A state in which one has lost interest in one’s surroundings and is unable to take voluntary action. |
| 26 |  | Disinhibition | A state in which one is unable to suppress one’s emotions or desires and uses speech and actions that are not acceptable in society. |
| 27 |  | Aberrant motor behavior | A state in which one engages in unusual behavior (e.g., wanders around or checks something excessively). |
| 28 |  | Negativism | A state in which one refuses everything that is offered, including going to day service (Refusal to eat should be classified as anorexia). |
| 29 |  | Delirium | A state in which one’s psychological state deteriorates owing to being in an environment that is different from normal, such as being in a hospital or being ill. |
| 30 |  | Other psychiatric symptom | Other symptoms such as being excessively dependent on caregivers (dependence) or excessively persistent (obsession). |

|  | **Symptom domain** | **Symptom** | **Explanation** |
| --- | --- | --- | --- |
| 31 | Eating behavior-related problems | Loss of appetite | A state in which one has little or no desire to eat. |
| 32 |  | Increase in appetite | A state in which one has a heightened desire to eat. |
| 33 |  | Weight loss | A state in which one loses weight. |
| 34 |  | Weight gain | A state in which one gains weight. |
| 35 |  | Food refusal | A state in which one says no to meals or refuses to eat even when being encouraged to eat. |
| 36 |  | Eating non-edible things | A state in which one eats something that is not food. |
| 37 |  | Unbalanced diet | A state in which one is very picky about meals. |
| 38 | Sleep-related disorders | Rapid eye movement sleep behavior disorder | A state of sleep (dreaming) in which one talks in long sentences or talks as if in a conversation, yells, or does things like kicking and punching. |
| 39 |  | Daytime somnolence | A state in which one falls asleep during the daytime even after a good night’s sleep. |
| 40 |  | Day-night reversal | A state in which one stays awake during the night and sleeps during the day. |
| 41 |  | Night-time sleep disorder | A state in which one is unable to sleep well, wakes up many times at night, and wakes up early in the morning. |
| 42 |  | Sudden sleep | A state in which one suddenly falls asleep despite having been awake until that time. |
| 43 |  | Restless legs syndrome | A state in which one feels restless in the legs while sitting or lying down. |
| 44 |  | Periodic limb movement disorder | A state in which one or both arms or legs involuntarily move periodically and repeatedly at a set interval. |
| 45 | Autonomic dysfunction | Orthostatic hypotension | A state in which one feels like they are losing color or fainting, goes pale, or becomes dizzy when standing up from sitting or lying down. |
| 46 |  | Disturbance of sweating | A state in which one sweats heavily only on the upper body or sweats only on the upper body despite feeling cold. |
| 47 |  | Constipation | A state in which one has poor bowel movements, which are sometimes accompanied by pain. |
| 48 |  | Night-time dysuria | Frequent urination (one gets up to go to the toilet three or more times during sleep), urinary incontinence, and a sensation of residual urine (one feels the urge to urinate even after urination). |
| 49 |  | Daytime dysuria | Frequent urination (one urinates eight or more times during the day), urinary incontinence, and a sensation of residual urine (one feels the urge to urinate even after urination). |
| 50 |  | Syncope | A state in which one experiences a temporary loss of consciousness but recovers after a few minutes (often observed after a meal or going to the toilet). |
| 51 |  | Dizziness | Dizziness is a term used to describe a range of sensations, such as feeling faint, woozy, weak or unsteady. |
| 52 | Sensory  disorders | Dysosmia | Dysosmia is a disorder described as any qualitative alteration or distortion of the perception of smell. |

Q1. Do you know a lot about dementia with Lewy Bodies (DLB)?

| 1  2  3 | I know a lot about it.  Neither yes nor no.  I do not know very much about it. | Please circle only one item. |
| --- | --- | --- |

Q2. Does your physician who treats you for DLB listen to what you say?

| 1  2  3  4  5  6 | My physician listens to me very well.  My physician listens to me.  My physician sometimes listens to me.  My physician does not listen to me much.  My physician does not listen to me at all.  I do not know. | Please circle only one item. |
| --- | --- | --- |

Q3. Is there someone at the hospital or clinic other than your physician with whom you can talk?

| 1  2  3 | Yes.  No.  I do not know. | Please circle only one item. |
| --- | --- | --- |

Q4. When prescribing medicine, does your physician explain about it to you?

| 1  2  3  4 | My physician explains.  My physician explains partially.  My physician does not explain.  I do not know. | Please circle only one item. |
| --- | --- | --- |

Q5. Have you ever experienced an issue after taking medicine prescribed by your physician?

| 1  2  3 | Yes.  No.  I do not know. | Please circle only one item. |
| --- | --- | --- |

Q6. If you selected “Yes” in Q5, what issue did you experience after taking medicine?

| Details of the issue |  |
| --- | --- |

Q7. If you selected “Yes” in Q5, did you inform your physician about the issue you experienced after taking medicine on your own or with the help of your caregiver or family member?

| 1  2  3 | Yes.  No.  I do not know. | Please circle only one item. |
| --- | --- | --- |

Q8. Are you taking medicine as instructed by your physician or pharmacist?

| 1  2  3 | Yes.  No.  I do not know. | Please circle only one item. |
| --- | --- | --- |

Q9. If you selected “No” in Q8 and stated that you are not taking medicine as instructed, what is the reason for not taking medicine as instructed or for not being able to take it properly?

| 1  2  3  4  5  6 | I forgot to take it.  I have difficulties swallowing it.  You may circle multiple items.  It does not work.  I am worried that a side effect may occur in the future.  I do not know.  Other reason (specifically： ) |
| --- | --- |

Q10. If you selected “I forgot to take it” in Q9, which medicine did you forget to take or were you unable to take properly?

| 1  2 | Medicine name: [ ] I do not know. | Please circle only one item. |
| --- | --- | --- |

Q11. Please select all of the symptoms that currently apply to you.

|  |  | **Symptom domain** | **Symptom** | **Explanation** |
| --- | --- | --- | --- | --- |
| You may circle multiple items. | 1 | Cognitive impairment | Memory impairment | A state in which one’s memory becomes extremely bad or one cannot recall things even with a hint. |
|  | 2 |  | Disorientation | A state in which one cannot tell what time of day it is or where they are. |
|  | 3 |  | Executive dysfunction | A state in which planning ahead to do something or to act in accordance with a procedure becomes difficult. |
|  | 4 |  | Attention dysfunction | A state in which one feels distracted or one’s attention or concentration is lost. |
|  | 5 |  | Fluctuating cognition | A state in which the cognitive function level varies from good to bad (stupor) and response levels fluctuate between lucid to reduced alertness. |
|  | 6 |  | Visuospatial dysfunction | A state in which one is unable to find something that is in front of them or is unable to accurately recognize something they see. |
|  | 7 |  | Other cognitive impairment | Other symptoms such as difficulty in speaking words (aphasia), difficulty in using items (apraxia), telling far-fetched stories (fabrication). |
|  | 8 | Parkinsonism | Bradykinesia/  Akinesia | A state in which it is difficult to move the body or move quickly. |
|  | 9 |  | Rigidity | A state in which relaxing the limbs or body is difficult, indicating that the muscles are always tense and stiff. |
|  | 10 |  | Action tremor | A state in which one’s hands shake when holding or writing something or one’s legs shake when sitting cross-legged. |
|  | 11 |  | Rest tremor | A state in which one’s hands and legs shake while resting relaxed, unlike when shaking occurs when trying to hold or write something. |
|  | 12 |  | Postural instability | A state in which keeping one’s balance is difficult and one feels like falling over. |
|  | 13 |  | Gait disturbance (short-stepped gait) | A state in which taking the first step is difficult or one shuffles or takes small steps. |
|  | 14 |  | Freezing of gait | A state in which taking the first step is difficult or one stands rooted to the spot and is unable to take the first step. |
|  | 15 |  | Abnormal posture | A state in which one’s neck is lowered or has a hunchback or forward-leaning posture. |
|  | 16 |  | Salivation | A state in which one is unable to swallow saliva and drools. |
|  | 17 |  | Fall | A state in which one often falls over on a flat road or stairs or falls off a chair from a sitting position. |
|  | 18 |  | Dysphagia | A state in which one has difficulty swallowing food and chokes or spits it out. |
|  | 19 | Psychiatric symptoms | Delusions | A state in which one falsely believes that their spouse is an imposter or is having an affair or that something was stolen. |
|  | 20 |  | Visual hallucinations | A state in which one sees something that does not really exist (person, animal, etc.). |
|  | 21 |  | Hallucinations other than visual hallucinations | A state in which one hears the voice of someone who is not present (auditory hallucination) or feels an illusory sensation and says something like “An insect is crawling under my skin” (cenesthesic hallucination). |
|  | 22 |  | Agitation/  Aggression | A state in which one has a heightened sense of emotion, speaks violent words, or resorts to violence. |
|  | 23 |  | Depression | A state in which one is depressed and has no motivation. |
|  | 24 |  | Anxiety | A state in which one feels restless or fidgety. |
|  | 25 |  | Apathy | A state in which one has lost interest in one’s surroundings and is unable to take voluntary action. |
|  | 26 |  | Disinhibition | A state in which one is unable to suppress one’s emotions or desires and uses speech and actions that are not acceptable in society. |
|  | 27 |  | Aberrant motor behavior | A state in which one engages in unusual behavior (e.g., wanders around or checks something excessively). |
|  | 28 |  | Negativism | A state in which one refuses everything that is offered, including going to day service (Refusal to eat should be classified as anorexia). |
|  | 29 |  | Delirium | A state in which one’s psychological state deteriorates owing to being in an environment that is different from normal, such as being in a hospital or being ill. |
|  | 30 |  | Other psychiatric symptom | Other symptoms such as being excessively dependent on caregivers (dependence) or excessively persistent (obsession). |

|  |  | **Symptom domain** | **Symptom** | **Explanation** |
| --- | --- | --- | --- | --- |
| You may circle multiple items. | 31 | Eating behavior-related problems | Loss of appetite | A state in which one has little or no desire to eat. |
|  | 32 |  | Increase in appetite | A state in which one has a heightened desire to eat. |
|  | 33 |  | Weight loss | A state in which one loses weight. |
|  | 34 |  | Weight gain | A state in which one gains weight. |
|  | 35 |  | Food refusal | A state in which one says no to meals or refuses to eat even when being encouraged to eat. |
|  | 36 |  | Eating non-edible  things | A state in which one eats something that is not food. |
|  | 37 |  | Unbalanced diet | A state in which one is very picky about meals. |
|  | 38 | Sleep-related disorders | Rapid eye movement sleep behavior disorder | A state of sleep (dreaming) in which one talks in long sentences or talks as if in a conversation, yells, or does things like kicking and punching. |
|  | 39 |  | Daytime somnolence | A state in which one falls asleep during the daytime even after a good night’s sleep. |
|  | 40 |  | Day-night reversal | A state in which one stays awake during the night and sleeps during the day. |
|  | 41 |  | Night-time sleep disorder | A state in which one is unable to sleep well, wakes up many times at night, and wakes up early in the morning. |
|  | 42 |  | Sudden sleep | A state in which one suddenly falls asleep despite having been awake until that time. |
|  | 43 |  | Restless legs syndrome | A state in which one feels restless in the legs while sitting or lying down. |
|  | 44 |  | Periodic limb movement disorder | A state in which one or both arms or legs involuntarily move periodically and repeatedly at a set interval. |
|  | 45 | Autonomic dysfunction | Orthostatic hypotension | A state in which one feels like they are losing color or fainting, goes pale, or becomes dizzy when standing up from sitting or lying down. |
|  | 46 |  | Disturbance of  sweating | A state in which one sweats heavily only on the upper body or sweats only on the upper body despite feeling cold. |
|  | 47 |  | Constipation | A state in which one has poor bowel movements, which are sometimes accompanied by pain. |
|  | 48 |  | Night-time dysuria | Frequent urination (one gets up to go to the toilet three or more times during sleep), urinary incontinence, and a sensation of residual urine (one feels the urge to urinate even after urination). |
|  | 49 |  | Daytime dysuria | Frequent urination (one urinates eight or more times during the day), urinary incontinence, and a sensation of residual urine (one feels the urge to urinate even after urination). |
|  | 50 |  | Syncope | A state in which one experiences a temporary loss of consciousness but recovers after a few minutes (often observed after a meal or going to the toilet). |
|  | 51 |  | Dizziness | Dizziness is a term used to describe a range of sensations, such as feeling faint, woozy, weak or unsteady. |
|  | 52 | Sensory  disorders | Dysosmia | Dysosmia is a disorder described as any qualitative alteration or distortion of the perception of smell. |
|  | 53 | I do not know. | | |

Q12. Please select all of the symptoms for which you think you are currently receiving treatment.

|  |  | **Symptom domain** | **Symptom** | **Explanation** |
| --- | --- | --- | --- | --- |
| You may circle multiple items. | 1 | Cognitive impairment | Memory impairment | A state in which one’s memory becomes extremely bad or one cannot recall things even with a hint. |
|  | 2 |  | Disorientation | A state in which one cannot tell what time of day it is or where they are. |
|  | 3 |  | Executive dysfunction | A state in which planning ahead to do something or to act in accordance with a procedure becomes difficult. |
|  | 4 |  | Attention dysfunction | A state in which one feels distracted or one’s attention or concentration is lost. |
|  | 5 |  | Fluctuating cognition | A state in which the cognitive function level varies from good to bad (stupor) and response levels fluctuate between lucid to reduced alertness. |
|  | 6 |  | Visuospatial dysfunction | A state in which one is unable to find something that is in front of them or is unable to accurately recognize something they see. |
|  | 7 |  | Other cognitive impairment | Other symptoms such as difficulty in speaking words (aphasia), difficulty in using items (apraxia), telling far-fetched stories (fabrication). |
|  | 8 | Parkinsonism | Bradykinesia/  Akinesia | A state in which it is difficult to move the body or move quickly. |
|  | 9 |  | Rigidity | A state in which relaxing the limbs or body is difficult, indicating that the muscles are always tense and stiff. |
|  | 10 |  | Action tremor | A state in which one’s hands shake when holding or writing something or one’s legs shake when sitting cross-legged. |
|  | 11 |  | Rest tremor | A state in which one’s hands and legs shake while resting relaxed, unlike when shaking occurs when trying to hold or write something. |
|  | 12 |  | Postural instability | A state in which keeping one’s balance is difficult and one feels like falling over. |
|  | 13 |  | Gait disturbance (short-stepped gait) | A state in which taking the first step is difficult or one shuffles or takes small steps. |
|  | 14 |  | Freezing of gait | A state in which taking the first step is difficult or one stands rooted to the spot and is unable to take the first step. |
|  | 15 |  | Abnormal posture | A state in which one’s neck is lowered or has a hunchback or forward-leaning posture. |
|  | 16 |  | Salivation | A state in which one is unable to swallow saliva and drools. |
|  | 17 |  | Fall | A state in which one often falls over on a flat road or stairs or falls off a chair from a sitting position. |
|  | 18 |  | Dysphagia | A state in which one has difficulty swallowing food and chokes or spits it out. |
|  | 19 | Psychiatric symptoms | Delusions | A state in which one falsely believes that their spouse is an imposter or is having an affair or that something was stolen. |
|  | 20 |  | Visual hallucinations | A state in which one sees something that does not really exist (person, animal, etc.). |
|  | 21 |  | Hallucinations other than visual hallucinations | A state in which one hears the voice of someone who is not present (auditory hallucination) or feels an illusory sensation and says something like “An insect is crawling under my skin” (cenesthesic hallucination). |
|  | 22 |  | Agitation/  Aggression | A state in which one has a heightened sense of emotion, speaks violent words, or resorts to violence. |
|  | 23 |  | Depression | A state in which one is depressed and has no motivation. |
|  | 24 |  | Anxiety | A state in which one feels restless or fidgety. |
|  | 25 |  | Apathy | A state in which one has lost interest in one’s surroundings and is unable to take voluntary action. |
|  | 26 |  | Disinhibition | A state in which one is unable to suppress one’s emotions or desires and uses speech and actions that are not acceptable in society. |
|  | 27 |  | Aberrant motor behavior | A state in which one engages in unusual behavior (e.g., wanders around or checks something excessively). |
|  | 28 |  | Negativism | A state in which one refuses everything that is offered, including going to day service (Refusal to eat should be classified as anorexia). |
|  | 29 |  | Delirium | A state in which one’s psychological state deteriorates owing to being in an environment that is different from normal, such as being in a hospital or being ill. |
|  | 30 |  | Other psychiatric symptom | Other symptoms such as being excessively dependent on caregivers (dependence) or excessively persistent (obsession). |

|  |  | **Symptom domain** | **Symptom** | **Explanation** |
| --- | --- | --- | --- | --- |
| You may circle multiple items. | 31 | Eating behavior-related problems | Loss of appetite | A state in which one has little or no desire to eat. |
|  | 32 |  | Increase in appetite | A state in which one has a heightened desire to eat. |
|  | 33 |  | Weight loss | A state in which one loses weight. |
|  | 34 |  | Weight gain | A state in which one gains weight. |
|  | 35 |  | Food refusal | A state in which one says no to meals or refuses to eat even when being encouraged to eat. |
|  | 36 |  | Eating non-edible  things | A state in which one eats something that is not food. |
|  | 37 |  | Unbalanced diet | A state in which one is very picky about meals. |
|  | 38 | Sleep-related disorders | Rapid eye movement sleep behavior disorder | A state of sleep (dreaming) in which one talks in long sentences or talks as if in a conversation, yells, or does things like kicking and punching. |
|  | 39 |  | Daytime somnolence | A state in which one falls asleep during the daytime even after a good night’s sleep. |
|  | 40 |  | Day-night reversal | A state in which one stays awake during the night and sleeps during the day. |
|  | 41 |  | Night-time sleep disorder | A state in which one is unable to sleep well, wakes up many times at night, and wakes up early in the morning. |
|  | 42 |  | Sudden sleep | A state in which one suddenly falls asleep despite having been awake until that time. |
|  | 43 |  | Restless legs syndrome | A state in which one feels restless in the legs while sitting or lying down. |
|  | 44 |  | Periodic limb movement disorder | A state in which one or both arms or legs involuntarily move periodically and repeatedly at a set interval. |
|  | 45 | Autonomic dysfunction | Orthostatic hypotension | A state in which one feels like they are losing color or fainting, goes pale, or becomes dizzy when standing up from sitting or lying down. |
|  | 46 |  | Disturbance of  sweating | A state in which one sweats heavily only on the upper body or sweats only on the upper body despite feeling cold. |
|  | 47 |  | Constipation | A state in which one has poor bowel movements, which are sometimes accompanied by pain. |
|  | 48 |  | Night-time dysuria | Frequent urination (one gets up to go to the toilet three or more times during sleep), urinary incontinence, and a sensation of residual urine (one feels the urge to urinate even after urination). |
|  | 49 |  | Daytime dysuria | Frequent urination (one urinates eight or more times during the day), urinary incontinence, and a sensation of residual urine (one feels the urge to urinate even after urination). |
|  | 50 |  | Syncope | A state in which one experiences a temporary loss of consciousness but recovers after a few minutes (often observed after a meal or going to the toilet). |
|  | 51 |  | Dizziness | Dizziness is a term used to describe a range of sensations, such as feeling faint, woozy, weak or unsteady. |
|  | 52 | Sensory  disorders | Dysosmia | Dysosmia is a disorder described as any qualitative alteration or distortion of the perception of smell. |
|  | 53 | I do not know. | | |

Q13. Please select only one symptom that currently causes you the most distress.

|  |  | **Symptom domain** | **Symptom** | **Explanation** |
| --- | --- | --- | --- | --- |
| Please circle only one item. | 1 | Cognitive impairment | Memory impairment | A state in which one’s memory becomes extremely bad or one cannot recall things even with a hint. |
|  | 2 |  | Disorientation | A state in which one cannot tell what time of day it is or where they are. |
|  | 3 |  | Executive dysfunction | A state in which planning ahead to do something or to act in accordance with a procedure becomes difficult. |
|  | 4 |  | Attention dysfunction | A state in which one feels distracted or one’s attention or concentration is lost. |
|  | 5 |  | Fluctuating cognition | A state in which the cognitive function level varies from good to bad (stupor) and response levels fluctuate between lucid to reduced alertness. |
|  | 6 |  | Visuospatial dysfunction | A state in which one is unable to find something that is in front of them or is unable to accurately recognize something they see. |
|  | 7 |  | Other cognitive impairment | Other symptoms such as difficulty in speaking words (aphasia), difficulty in using items (apraxia), telling far-fetched stories (fabrication). |
|  | 8 | Parkinsonism | Bradykinesia/  Akinesia | A state in which it is difficult to move the body or move quickly. |
|  | 9 |  | Rigidity | A state in which relaxing the limbs or body is difficult, indicating that the muscles are always tense and stiff. |
|  | 10 |  | Action tremor | A state in which one’s hands shake when holding or writing something or one’s legs shake when sitting cross-legged. |
|  | 11 |  | Rest tremor | A state in which one’s hands and legs shake while resting relaxed, unlike when shaking occurs when trying to hold or write something. |
|  | 12 |  | Postural instability | A state in which keeping one’s balance is difficult and one feels like falling over. |
|  | 13 |  | Gait disturbance (short-stepped gait) | A state in which taking the first step is difficult or one shuffles or takes small steps. |
|  | 14 |  | Freezing of gait | A state in which taking the first step is difficult or one stands rooted to the spot and is unable to take the first step. |
|  | 15 |  | Abnormal posture | A state in which one’s neck is lowered or has a hunchback or forward-leaning posture. |
|  | 16 |  | Salivation | A state in which one is unable to swallow saliva and drools. |
|  | 17 |  | Fall | A state in which one often falls over on a flat road or stairs or falls off a chair from a sitting position. |
|  | 18 |  | Dysphagia | A state in which one has difficulty swallowing food and chokes or spits it out. |
|  | 19 | Psychiatric symptoms | Delusions | A state in which one falsely believes that their spouse is an imposter or is having an affair or that something was stolen. |
|  | 20 |  | Visual hallucinations | A state in which one sees something that does not really exist (person, animal, etc.). |
|  | 21 |  | Hallucinations other than visual hallucinations | A state in which one hears the voice of someone who is not present (auditory hallucination) or feels an illusory sensation and says something like “An insect is crawling under my skin” (cenesthesic hallucination). |
|  | 22 |  | Agitation/  Aggression | A state in which one has a heightened sense of emotion, speaks violent words, or resorts to violence. |
|  | 23 |  | Depression | A state in which one is depressed and has no motivation. |
|  | 24 |  | Anxiety | A state in which one feels restless or fidgety. |
|  | 25 |  | Apathy | A state in which one has lost interest in one’s surroundings and is unable to take voluntary action. |
|  | 26 |  | Disinhibition | A state in which one is unable to suppress one’s emotions or desires and uses speech and actions that are not acceptable in society. |
|  | 27 |  | Aberrant motor behavior | A state in which one engages in unusual behavior (e.g., wanders around or checks something excessively). |
|  | 28 |  | Negativism | A state in which one refuses everything that is offered, including going to day service (Refusal to eat should be classified as anorexia). |
|  | 29 |  | Delirium | A state in which one’s psychological state deteriorates owing to being in an environment that is different from normal, such as being in a hospital or being ill. |
|  | 30 |  | Other psychiatric symptom | Other symptoms such as being excessively dependent on caregivers (dependence) or excessively persistent (obsession). |

|  |  | **Symptom domain** | **Symptom** | **Explanation** |
| --- | --- | --- | --- | --- |
| Please circle only one item. | 31 | Eating behavior-related problems | Loss of appetite | A state in which one has little or no desire to eat. |
|  | 32 |  | Increase in appetite | A state in which one has a heightened desire to eat. |
|  | 33 |  | Weight loss | A state in which one loses weight. |
|  | 34 |  | Weight gain | A state in which one gains weight. |
|  | 35 |  | Food refusal | A state in which one says no to meals or refuses to eat even when being encouraged to eat. |
|  | 36 |  | Eating non-edible  things | A state in which one eats something that is not food. |
|  | 37 |  | Unbalanced diet | A state in which one is very picky about meals. |
|  | 38 | Sleep-related disorders | Rapid eye movement sleep behavior disorder | A state of sleep (dreaming) in which one talks in long sentences or talks as if in a conversation, yells, or does things like kicking and punching. |
|  | 39 |  | Daytime somnolence | A state in which one falls asleep during the daytime even after a good night’s sleep. |
|  | 40 |  | Day-night reversal | A state in which one stays awake during the night and sleeps during the day. |
|  | 41 |  | Night-time sleep disorder | A state in which one is unable to sleep well, wakes up many times at night, and wakes up early in the morning. |
|  | 42 |  | Sudden sleep | A state in which one suddenly falls asleep despite having been awake until that time. |
|  | 43 |  | Restless legs syndrome | A state in which one feels restless in the legs while sitting or lying down. |
|  | 44 |  | Periodic limb movement disorder | A state in which one or both arms or legs involuntarily move periodically and repeatedly at a set interval. |
|  | 45 | Autonomic dysfunction | Orthostatic hypotension | A state in which one feels like they are losing color or fainting, goes pale, or becomes dizzy when standing up from sitting or lying down. |
|  | 46 |  | Disturbance of  sweating | A state in which one sweats heavily only on the upper body or sweats only on the upper body despite feeling cold. |
|  | 47 |  | Constipation | A state in which one has poor bowel movements, which are sometimes accompanied by pain. |
|  | 48 |  | Night-time dysuria | Frequent urination (one gets up to go to the toilet three or more times during sleep), urinary incontinence, and a sensation of residual urine (one feels the urge to urinate even after urination). |
|  | 49 |  | Daytime dysuria | Frequent urination (one urinates eight or more times during the day), urinary incontinence, and a sensation of residual urine (one feels the urge to urinate even after urination). |
|  | 50 |  | Syncope | A state in which one experiences a temporary loss of consciousness but recovers after a few minutes (often observed after a meal or going to the toilet). |
|  | 51 |  | Dizziness | Dizziness is a term used to describe a range of sensations, such as feeling faint, woozy, weak or unsteady. |
|  | 52 | Sensory  disorders | Dysosmia | Dysosmia is a disorder described as any qualitative alteration or distortion of the perception of smell. |
|  | 53 | I do not know. | | |

Q14. If you responded to Q13 and selected the symptom that causes you the most distress, what was your reason for selecting that symptom (selected in Q13)?

| Reason |  |
| --- | --- |

Q15. If you responded to Q13 and selected the symptom that causes you the most distress, have you informed your physician about that symptom (selected in Q13)?

| 1  2  3 | Yes.  No.  I do not know. | Please circle only one item. |
| --- | --- | --- |

Q16. If you responded to Q13 and selected the symptom that causes you the most distress, has your physician inquired about that symptom (selected in Q13)?

| 1  2  3 | Yes.  No.  I do not know. | Please circle only one item. |
| --- | --- | --- |

Q17. If you responded to Q13 and selected the symptom that causes you the most distress, has your caregiver or family member been informed about that symptom (selected in Q13)?

| 1  2  3 | Yes.  No.  I do not know. | Please circle only one item. |
| --- | --- | --- |

Q18. If you responded to Q13 and selected the symptom that causes you the most distress, has your caregiver or family member inquired about that symptom (selected in Q13)?

| 1  2  3 | Yes.  No.  I do not know. | Please circle only one item. |
| --- | --- | --- |

| Please proceed to the next page ▶▶▶ |
| --- |

Q19. Please select all symptoms other than the symptom that currently causes you the most distress.

|  |  | **Symptom domain** | **Symptom** | **Explanation** |
| --- | --- | --- | --- | --- |
| You may circle multiple items. | 1 | Cognitive impairment | Memory impairment | A state in which one’s memory becomes extremely bad or one cannot recall things even with a hint. |
|  | 2 |  | Disorientation | A state in which one cannot tell what time of day it is or where they are. |
|  | 3 |  | Executive dysfunction | A state in which planning ahead to do something or to act in accordance with a procedure becomes difficult. |
|  | 4 |  | Attention dysfunction | A state in which one feels distracted or one’s attention or concentration is lost. |
|  | 5 |  | Fluctuating cognition | A state in which the cognitive function level varies from good to bad (stupor) and response levels fluctuate between lucid to reduced alertness. |
|  | 6 |  | Visuospatial dysfunction | A state in which one is unable to find something that is in front of them or is unable to accurately recognize something they see. |
|  | 7 |  | Other cognitive impairment | Other symptoms such as difficulty in speaking words (aphasia), difficulty in using items (apraxia), telling far-fetched stories (fabrication). |
|  | 8 | Parkinsonism | Bradykinesia/  Akinesia | A state in which it is difficult to move the body or move quickly. |
|  | 9 |  | Rigidity | A state in which relaxing the limbs or body is difficult, indicating that the muscles are always tense and stiff. |
|  | 10 |  | Action tremor | A state in which one’s hands shake when holding or writing something or one’s legs shake when sitting cross-legged. |
|  | 11 |  | Rest tremor | A state in which one’s hands and legs shake while resting relaxed, unlike when shaking occurs when trying to hold or write something. |
|  | 12 |  | Postural instability | A state in which keeping one’s balance is difficult and one feels like falling over. |
|  | 13 |  | Gait disturbance (short-stepped gait) | A state in which taking the first step is difficult or one shuffles or takes small steps. |
|  | 14 |  | Freezing of gait | A state in which taking the first step is difficult or one stands rooted to the spot and is unable to take the first step. |
|  | 15 |  | Abnormal posture | A state in which one’s neck is lowered or has a hunchback or forward-leaning posture. |
|  | 16 |  | Salivation | A state in which one is unable to swallow saliva and drools. |
|  | 17 |  | Fall | A state in which one often falls over on a flat road or stairs or falls off a chair from a sitting position. |
|  | 18 |  | Dysphagia | A state in which one has difficulty swallowing food and chokes or spits it out. |
|  | 19 | Psychiatric symptoms | Delusions | A state in which one falsely believes that their spouse is an imposter or is having an affair or that something was stolen. |
|  | 20 |  | Visual hallucinations | A state in which one sees something that does not really exist (person, animal, etc.). |
|  | 21 |  | Hallucinations other than visual hallucinations | A state in which one hears the voice of someone who is not present (auditory hallucination) or feels an illusory sensation and says something like “An insect is crawling under my skin” (cenesthesic hallucination). |
|  | 22 |  | Agitation/  Aggression | A state in which one has a heightened sense of emotion, speaks violent words, or resorts to violence. |
|  | 23 |  | Depression | A state in which one is depressed and has no motivation. |
|  | 24 |  | Anxiety | A state in which one feels restless or fidgety. |
|  | 25 |  | Apathy | A state in which one has lost interest in one’s surroundings and is unable to take voluntary action. |
|  | 26 |  | Disinhibition | A state in which one is unable to suppress one’s emotions or desires and uses speech and actions that are not acceptable in society. |
|  | 27 |  | Aberrant motor behavior | A state in which one engages in unusual behavior (e.g., wanders around or checks something excessively). |
|  | 28 |  | Negativism | A state in which one refuses everything that is offered, including going to day service (Refusal to eat should be classified as anorexia). |
|  | 29 |  | Delirium | A state in which one’s psychological state deteriorates owing to being in an environment that is different from normal, such as being in a hospital or being ill. |
|  | 30 |  | Other psychiatric symptom | Other symptoms such as being excessively dependent on caregivers (dependence) or excessively persistent (obsession). |

|  |  | **Symptom domain** | **Symptom** | **Explanation** |
| --- | --- | --- | --- | --- |
| You may circle multiple items. | 31 | Eating behavior-related problems | Loss of appetite | A state in which one has little or no desire to eat. |
|  | 32 |  | Increase in appetite | A state in which one has a heightened desire to eat. |
|  | 33 |  | Weight loss | A state in which one loses weight. |
|  | 34 |  | Weight gain | A state in which one gains weight. |
|  | 35 |  | Food refusal | A state in which one says no to meals or refuses to eat even when being encouraged to eat. |
|  | 36 |  | Eating non-edible  things | A state in which one eats something that is not food. |
|  | 37 |  | Unbalanced diet | A state in which one is very picky about meals. |
|  | 38 | Sleep-related disorders | Rapid eye movement sleep behavior disorder | A state of sleep (dreaming) in which one talks in long sentences or talks as if in a conversation, yells, or does things like kicking and punching. |
|  | 39 |  | Daytime somnolence | A state in which one falls asleep during the daytime even after a good night’s sleep. |
|  | 40 |  | Day-night reversal | A state in which one stays awake during the night and sleeps during the day. |
|  | 41 |  | Night-time sleep disorder | A state in which one is unable to sleep well, wakes up many times at night, and wakes up early in the morning. |
|  | 42 |  | Sudden sleep | A state in which one suddenly falls asleep despite having been awake until that time. |
|  | 43 |  | Restless legs syndrome | A state in which one feels restless in the legs while sitting or lying down. |
|  | 44 |  | Periodic limb movement disorder | A state in which one or both arms or legs involuntarily move periodically and repeatedly at a set interval. |
|  | 45 | Autonomic dysfunction | Orthostatic hypotension | A state in which one feels like they are losing color or fainting, goes pale, or becomes dizzy when standing up from sitting or lying down. |
|  | 46 |  | Disturbance of  sweating | A state in which one sweats heavily only on the upper body or sweats only on the upper body despite feeling cold. |
|  | 47 |  | Constipation | A state in which one has poor bowel movements, which are sometimes accompanied by pain. |
|  | 48 |  | Night-time dysuria | Frequent urination (one gets up to go to the toilet three or more times during sleep), urinary incontinence, and a sensation of residual urine (one feels the urge to urinate even after urination). |
|  | 49 |  | Daytime dysuria | Frequent urination (one urinates eight or more times during the day), urinary incontinence, and a sensation of residual urine (one feels the urge to urinate even after urination). |
|  | 50 |  | Syncope | A state in which one experiences a temporary loss of consciousness but recovers after a few minutes (often observed after a meal or going to the toilet). |
|  | 51 |  | Dizziness | Dizziness is a term used to describe a range of sensations, such as feeling faint, woozy, weak or unsteady. |
|  | 52 | Sensory  disorders | Dysosmia | Dysosmia is a disorder described as any qualitative alteration or distortion of the perception of smell. |
|  | 53 | No other troubling symptoms. | | |
|  | 54 | I do not know. | | |

Q20. As you continue with treatment, which symptom would you most likely prioritize for receiving treatment? Please select only one.

|  |  | **Symptom domain** | **Symptom** | **Explanation** |
| --- | --- | --- | --- | --- |
| Please circle only one item. | 1 | Cognitive impairment | Memory impairment | A state in which one’s memory becomes extremely bad or one cannot recall things even with a hint. |
|  | 2 |  | Disorientation | A state in which one cannot tell what time of day it is or where they are. |
|  | 3 |  | Executive dysfunction | A state in which planning ahead to do something or to act in accordance with a procedure becomes difficult. |
|  | 4 |  | Attention dysfunction | A state in which one feels distracted or one’s attention or concentration is lost. |
|  | 5 |  | Fluctuating cognition | A state in which the cognitive function level varies from good to bad (stupor) and response levels fluctuate between lucid to reduced alertness. |
|  | 6 |  | Visuospatial dysfunction | A state in which one is unable to find something that is in front of them or is unable to accurately recognize something they see. |
|  | 7 |  | Other cognitive impairment | Other symptoms such as difficulty in speaking words (aphasia), difficulty in using items (apraxia), telling far-fetched stories (fabrication). |
|  | 8 | Parkinsonism | Bradykinesia/  Akinesia | A state in which it is difficult to move the body or move quickly. |
|  | 9 |  | Rigidity | A state in which relaxing the limbs or body is difficult, indicating that the muscles are always tense and stiff. |
|  | 10 |  | Action tremor | A state in which one’s hands shake when holding or writing something or one’s legs shake when sitting cross-legged. |
|  | 11 |  | Rest tremor | A state in which one’s hands and legs shake while resting relaxed, unlike when shaking occurs when trying to hold or write something. |
|  | 12 |  | Postural instability | A state in which keeping one’s balance is difficult and one feels like falling over. |
|  | 13 |  | Gait disturbance (short-stepped gait) | A state in which taking the first step is difficult or one shuffles or takes small steps. |
|  | 14 |  | Freezing of gait | A state in which taking the first step is difficult or one stands rooted to the spot and is unable to take the first step. |
|  | 15 |  | Abnormal posture | A state in which one’s neck is lowered or has a hunchback or forward-leaning posture. |
|  | 16 |  | Salivation | A state in which one is unable to swallow saliva and drools. |
|  | 17 |  | Fall | A state in which one often falls over on a flat road or stairs or falls off a chair from a sitting position. |
|  | 18 |  | Dysphagia | A state in which one has difficulty swallowing food and chokes or spits it out. |
|  | 19 | Psychiatric symptoms | Delusions | A state in which one falsely believes that their spouse is an imposter or is having an affair or that something was stolen. |
|  | 20 |  | Visual hallucinations | A state in which one sees something that does not really exist (person, animal, etc.). |
|  | 21 |  | Hallucinations other than visual hallucinations | A state in which one hears the voice of someone who is not present (auditory hallucination) or feels an illusory sensation and says something like “An insect is crawling under my skin” (cenesthesic hallucination). |
|  | 22 |  | Agitation/  Aggression | A state in which one has a heightened sense of emotion, speaks violent words, or resorts to violence. |
|  | 23 |  | Depression | A state in which one is depressed and has no motivation. |
|  | 24 |  | Anxiety | A state in which one feels restless or fidgety. |
|  | 25 |  | Apathy | A state in which one has lost interest in one’s surroundings and is unable to take voluntary action. |
|  | 26 |  | Disinhibition | A state in which one is unable to suppress one’s emotions or desires and uses speech and actions that are not acceptable in society. |
|  | 27 |  | Aberrant motor behavior | A state in which one engages in unusual behavior (e.g., wanders around or checks something excessively). |
|  | 28 |  | Negativism | A state in which one refuses everything that is offered, including going to day service (Refusal to eat should be classified as anorexia). |
|  | 29 |  | Delirium | A state in which one’s psychological state deteriorates owing to being in an environment that is different from normal, such as being in a hospital or being ill. |
|  | 30 |  | Other psychiatric symptom | Other symptoms such as being excessively dependent on caregivers (dependence) or excessively persistent (obsession). |

|  |  | **Symptom domain** | **Symptom** | **Explanation** |
| --- | --- | --- | --- | --- |
| Please circle only one item. | 31 | Eating behavior-related problems | Loss of appetite | A state in which one has little or no desire to eat. |
|  | 32 |  | Increase in appetite | A state in which one has a heightened desire to eat. |
|  | 33 |  | Weight loss | A state in which one loses weight. |
|  | 34 |  | Weight gain | A state in which one gains weight. |
|  | 35 |  | Food refusal | A state in which one says no to meals or refuses to eat even when being encouraged to eat. |
|  | 36 |  | Eating non-edible  things | A state in which one eats something that is not food. |
|  | 37 |  | Unbalanced diet | A state in which one is very picky about meals. |
|  | 38 | Sleep-related disorders | Rapid eye movement sleep behavior disorder | A state of sleep (dreaming) in which one talks in long sentences or talks as if in a conversation, yells, or does things like kicking and punching. |
|  | 39 |  | Daytime somnolence | A state in which one falls asleep during the daytime even after a good night’s sleep. |
|  | 40 |  | Day-night reversal | A state in which one stays awake during the night and sleeps during the day. |
|  | 41 |  | Night-time sleep disorder | A state in which one is unable to sleep well, wakes up many times at night, and wakes up early in the morning. |
|  | 42 |  | Sudden sleep | A state in which one suddenly falls asleep despite having been awake until that time. |
|  | 43 |  | Restless legs syndrome | A state in which one feels restless in the legs while sitting or lying down. |
|  | 44 |  | Periodic limb movement disorder | A state in which one or both arms or legs involuntarily move periodically and repeatedly at a set interval. |
|  | 45 | Autonomic dysfunction | Orthostatic hypotension | A state in which one feels like they are losing color or fainting, goes pale, or becomes dizzy when standing up from sitting or lying down. |
|  | 46 |  | Disturbance of  sweating | A state in which one sweats heavily only on the upper body or sweats only on the upper body despite feeling cold. |
|  | 47 |  | Constipation | A state in which one has poor bowel movements, which are sometimes accompanied by pain. |
|  | 48 |  | Night-time dysuria | Frequent urination (one gets up to go to the toilet three or more times during sleep), urinary incontinence, and a sensation of residual urine (one feels the urge to urinate even after urination). |
|  | 49 |  | Daytime dysuria | Frequent urination (one urinates eight or more times during the day), urinary incontinence, and a sensation of residual urine (one feels the urge to urinate even after urination). |
|  | 50 |  | Syncope | A state in which one experiences a temporary loss of consciousness but recovers after a few minutes (often observed after a meal or going to the toilet). |
|  | 51 |  | Dizziness | Dizziness is a term used to describe a range of sensations, such as feeling faint, woozy, weak or unsteady. |
|  | 52 | Sensory  disorders | Dysosmia | Dysosmia is a disorder described as any qualitative alteration or distortion of the perception of smell. |
|  | 53 | I do not know. | | |

Q21. Please confirm your response to Q11 on page 5–6.

Did you select any items in 19–44?

| 1  2 | Yes  No | ▶  ▶ | Go to Q22  Go to Q28 | Please circle only one item. |
| --- | --- | --- | --- | --- |

Q22. Did your physician explain about psychiatric symptoms, eating behavior-related problems, and sleep-related disorders to you?

* Please refer to the table on the following page regarding Psychiatric symptoms, Eating behavior-related problems, and Sleep-related disorders.

| 1  2  3  4 | My physician explained.  My physician explained partially.  My physician did not explain.  I do not know. | Please circle only one item. |
| --- | --- | --- |

Q23. Did your caregiver or family member explain about psychiatric symptoms, eating behavior-related problems, and sleep-related disorders to you?

* Please refer to the table on the following page regarding Psychiatric symptoms, Eating behavior-related problems, and Sleep-related disorders.

| 1  2  3  4 | My caregiver or family member explained.  My caregiver or family member explained partially.  My caregiver or family member did not explain.  I do not know. | Please circle only one item. |
| --- | --- | --- |

**※** Psychiatric symptoms, Eating behavior-related problems, and Sleep-related disorders are as follows:

|  | **Symptom** | **Explanation** |
| --- | --- | --- |
| Psychiatric symptoms | Delusions | A state in which one falsely believes that their spouse is an imposter or is having an affair or that something was stolen. |
|  | Visual hallucinations | A state in which one sees something that does not really exist (person, animal, etc.). |
|  | Hallucinations other than visual hallucinations | A state in which one hears the voice of someone who is not present (auditory hallucination) or feels an illusory sensation and says something like “An insect is crawling under my skin” (cenesthesic hallucination). |
|  | Agitation/Aggression | A state in which one has a heightened sense of emotion, speaks violent words, or resorts to violence. |
|  | Depression | A state in which one is depressed and has no motivation. |
|  | Anxiety | A state in which one feels restless or fidgety. |
|  | Apathy | A state in which one has lost interest in one’s surroundings and is unable to take voluntary action. |
|  | Disinhibition | A state in which one is unable to suppress one’s emotions or desires and uses speech and actions that are not acceptable in society. |
|  | Aberrant motor behavior | A state in which one engages in unusual behavior (e.g., wanders around or checks something excessively). |
|  | Negativism | A state in which one refuses everything that is offered, including going to day service (Refusal to eat should be classified as anorexia). |
|  | Delirium | A state in which one’s psychological state deteriorates owing to being in an environment that is different from normal, such as being in a hospital or being ill. |
|  | Other psychiatric symptom | Other symptoms such as being excessively dependent on caregivers (dependence) or excessively persistent (obsession). |
| Eating behavior-related problems | Loss of appetite | A state in which one has little or no desire to eat. |
|  | Increase in appetite | A state in which one has a heightened desire to eat. |
|  | Weight loss | A state in which one loses weight. |
|  | Weight gain | A state in which one gains weight. |
|  | Food refusal | A state in which one says no to meals or refuses to eat even when being encouraged to eat. |
|  | Eating non-edible things | A state in which one eats something that is not food. |
|  | Unbalanced diet | A state in which one is very picky about meals. |
| Sleep-related disorders | Rapid eye movement sleep behavior disorder | A state of sleep (dreaming) in which one talks in long sentences or talks as if in a conversation, yells, or does things like kicking and punching. |
|  | Daytime somnolence | A state in which one falls asleep during the daytime even after a good night’s sleep. |
|  | Day-night reversal | A state in which one stays awake during the night and sleeps during the day. |
|  | Night-time sleep disorder | A state in which one is unable to sleep well, wakes up many times at night, and wakes up early in the morning. |
|  | Sudden sleep | A state in which one suddenly falls asleep despite having been awake until that time. |
|  | Restless legs syndrome | A state in which one feels restless in the legs while sitting or lying down. |
|  | Periodic limb movement disorder | A state in which one or both arms or legs involuntarily move periodically and repeatedly at a set interval. |

Q24. If you selected any items in 19–44 in Q11, which is the symptom that causes you the most distress among those classified under psychiatric symptoms, eating behavior-related problems, and sleep related-disorders? Please select only one.

|  |  |  | **Symptom** | **Explanation** |
| --- | --- | --- | --- | --- |
| Please circle only one item. | 1 | Psychiatric symptoms | Delusions | A state in which one falsely believes that their spouse is an imposter or is having an affair or that something was stolen. |
|  | 2 |  | Visual hallucinations | A state in which one sees something that does not really exist (person, animal, etc.). |
|  | 3 |  | Hallucinations other than visual hallucinations | A state in which one hears the voice of someone who is not present (auditory hallucination) or feels an illusory sensation and says something like “An insect is crawling under my skin” (cenesthesic hallucination). |
|  | 4 |  | Agitation/Aggression | A state in which one has a heightened sense of emotion, speaks violent words, or resorts to violence. |
|  | 5 |  | Depression | A state in which one is depressed and has no motivation. |
|  | 6 |  | Anxiety | A state in which one feels restless or fidgety. |
|  | 7 |  | Apathy | A state in which one has lost interest in one’s surroundings and is unable to take voluntary action. |
|  | 8 |  | Disinhibition | A state in which one is unable to suppress one’s emotions or desires and uses speech and actions that are not acceptable in society. |
|  | 9 |  | Aberrant motor behavior | A state in which one engages in unusual behavior (e.g., wanders around or checks something excessively). |
|  | 10 |  | Negativism | A state in which one refuses everything that is offered, including going to day service (Refusal to eat should be classified as anorexia). |
|  | 11 |  | Delirium | A state in which one’s psychological state deteriorates owing to being in an environment that is different from normal, such as being in a hospital or being ill. |
|  | 12 |  | Other psychiatric symptom | Other symptoms such as being excessively dependent on caregivers (dependence) or excessively persistent (obsession). |
|  | 13 | Eating behavior-related problems | Loss of appetite | A state in which one has little or no desire to eat. |
|  | 14 |  | Increase in appetite | A state in which one has a heightened desire to eat. |
|  | 15 |  | Weight loss | A state in which one loses weight. |
|  | 16 |  | Weight gain | A state in which one gains weight. |
|  | 17 |  | Food refusal | A state in which one says no to meals or refuses to eat even when being encouraged to eat. |
|  | 18 |  | Eating non-edible things | A state in which one eats something that is not food. |
|  | 19 |  | Unbalanced diet | A state in which one is very picky about meals. |
|  | 20 | Sleep-related disorders | Rapid eye movement sleep behavior disorder | A state of sleep (dreaming) in which one talks in long sentences or talks as if in a conversation, yells, or does things like kicking and punching. |
|  | 21 |  | Daytime somnolence | A state in which one falls asleep during the daytime even after a good night’s sleep. |
|  | 22 |  | Day–night reversal | A state in which one stays awake during the night and sleeps during the day. |
|  | 23 |  | Night-time sleep disorder | A state in which one is unable to sleep well, wakes up many times at night, and wakes up early in the morning. |
|  | 24 |  | Sudden sleep | A state in which one suddenly falls asleep despite having been awake until that time. |
|  | 25 |  | Restless legs syndrome | A state in which one feels restless in the legs while sitting or lying down. |
|  | 26 |  | Periodic limb movement disorder | A state in which one or both arms or legs involuntarily move periodically and repeatedly at a set interval. |
|  | 27 | I do not know. | | |

Q25. If you responded to Q24 and selected the symptom that causes you the most distress, would you prefer to receive treatment for that symptom (selected in Q24)?

| 1  2  3 | Yes.  No.  I do not know. | Please circle only one item. |
| --- | --- | --- |

Q26. If you responded to Q24 and selected the symptom that causes you the most distress, do you think you are being treated (receiving medication or advice from your physician) for that symptom (selected in Q24)?

| 1  2  3 | Yes.  No.  I do not know. | Please circle only one item. |
| --- | --- | --- |

Q27. If you selected any items in Q24, as you continue with treatment, which symptoms would you prefer to prioritize for receiving treatment? Please select all applicable items.

|  |  |  | **Symptom** | **Explanation** |
| --- | --- | --- | --- | --- |
| You may circle multiple items. | 1 | Psychiatric symptoms | Delusions | A state in which one falsely believes that their spouse is an imposter or is having an affair or that something was stolen. |
|  | 2 |  | Visual hallucinations | A state in which one sees something that does not really exist (person, animal, etc.). |
|  | 3 |  | Hallucinations other than visual hallucinations | A state in which one hears the voice of someone who is not present (auditory hallucination) or feels an illusory sensation and says something like “An insect is crawling under my skin” (cenesthesic hallucination). |
|  | 4 |  | Agitation/Aggression | A state in which one has a heightened sense of emotion, speaks violent words, or resorts to violence. |
|  | 5 |  | Depression | A state in which one is depressed and has no motivation. |
|  | 6 |  | Anxiety | A state in which one feels restless or fidgety. |
|  | 7 |  | Apathy | A state in which one has lost interest in one’s surroundings and is unable to take voluntary action. |
|  | 8 |  | Disinhibition | A state in which one is unable to suppress one’s emotions or desires and uses speech and actions that are not acceptable in society. |
|  | 9 |  | Aberrant motor behavior | A state in which one engages in unusual behavior (e.g., wanders around or checks something excessively). |
|  | 10 |  | Negativism | A state in which one refuses everything that is offered, including going to day service (Refusal to eat should be classified as anorexia). |
|  | 11 |  | Delirium | A state in which one’s psychological state deteriorates owing to being in an environment that is different from normal, such as being in a hospital or being ill. |
|  | 12 |  | Other psychiatric symptom | Other symptoms such as being excessively dependent on caregivers (dependence) or excessively persistent (obsession). |
|  | 13 | Eating behavior-related problems | Loss of appetite | A state in which one has little or no desire to eat. |
|  | 14 |  | Increase in appetite | A state in which one has a heightened desire to eat. |
|  | 15 |  | Weight loss | A state in which one loses weight. |
|  | 16 |  | Weight gain | A state in which one gains weight. |
|  | 17 |  | Food refusal | A state in which one says no to meals or refuses to eat even when being encouraged to eat. |
|  | 18 |  | Eating non-edible things | A state in which one eats something that is not food. |
|  | 19 |  | Unbalanced diet | A state in which one is very picky about meals. |
|  | 20 | Sleep-related disorders | Rapid eye movement sleep behavior disorder | A state of sleep (dreaming) in which one talks in long sentences or talks as if in a conversation, yells, or does things like kicking and punching. |
|  | 21 |  | Daytime somnolence | A state in which one falls asleep during the daytime even after a good night’s sleep. |
|  | 22 |  | Day–night reversal | A state in which one stays awake during the night and sleeps during the day. |
|  | 23 |  | Night-time sleep disorder | A state in which one is unable to sleep well, wakes up many times at night, and wakes up early in the morning. |
|  | 24 |  | Sudden sleep | A state in which one suddenly falls asleep despite having been awake until that time. |
|  | 25 |  | Restless legs syndrome | A state in which one feels restless in the legs while sitting or lying down. |
|  | 26 |  | Periodic limb movement disorder | A state in which one or both arms or legs involuntarily move periodically and repeatedly at a set interval. |
|  | 27 | I do not know. | | |

Q28. Please confirm your response to Q11 on page 5–6.

Did you select any items in 8–18?

| 1  2 | Yes  No | ▶  ▶ | Go to Q29  Go to Q36 | Please circle only one item. |
| --- | --- | --- | --- | --- |

Q29. Did your physician explain about parkinsonism to you?

* Please refer to the table below regarding parkinsonism.

| 1  2  3  4 | My physician explained.  My physician explained partially.  My physician did not explain.  I do not know. | Please circle only one item. |
| --- | --- | --- |

**※** Parkinsonism has the following symptoms:

|  | **Symptom** | **Explanation** |
| --- | --- | --- |
| Parkinsonism | Bradykinesia/  Akinesia | A state in which it is difficult to move the body or move quickly. |
|  | Rigidity | A state in which relaxing the limbs or body is difficult, indicating that the muscles are always tense and stiff. |
|  | Action tremor | A state in which one’s hands shake when holding or writing something or one’s legs shake when sitting cross-legged. |
|  | Rest tremor | A state in which one’s hands and legs shake while resting relaxed, unlike when shaking occurs when trying to hold or write something. |
|  | Postural instability | A state in which keeping one’s balance is difficult and one feels like falling over. |
|  | Gait disturbance (short-stepped gait) | A state in which taking the first step is difficult or one shuffles or takes small steps. |
|  | Freezing of gait | A state in which taking the first step is difficult or one stands rooted to the spot and is unable to take the first step. |
|  | Abnormal posture | A state in which one’s neck is lowered or has a hunchback or forward-leaning posture. |
|  | Salivation | A state in which one is unable to swallow saliva and drools. |
|  | Fall | A state in which one often falls over on a flat road or stairs or falls off a chair from a sitting position. |
|  | Dysphagia | A state in which one has difficulty swallowing food and chokes or spits it out. |

Q30. Did your caregiver or family member explain about parkinsonism to you?

| 1  2  3  4 | My caregiver or family member explained.  My caregiver or family member explained partially.  My caregiver or family member did not explain.  I do not know. | Please circle only one item. |
| --- | --- | --- |

Q31. Levodopa formulations include Madopar and Menesit. Has a levodopa formulation been prescribed to you? What was its effect on parkinsonism when you started taking it? Please select one applicable item.

| 1  2  3  4  5  6  7 | It was extremely effective.  It was moderately effective.  No change was observed.  It was not very effective.  It was not effective at all.  I have never taken it.  I do not know. I cannot recall. | Please circle only one item. |
| --- | --- | --- |

Q32. If you selected any items in 8–18 in Q11, which is the symptom of parkinsonism that causes you the most distress? Please select only one.

|  |  |  | **Symptom** | **Explanation** |
| --- | --- | --- | --- | --- |
| Please circle only one item. | 1 | Parkinsonism | Bradykinesia/  Akinesia | A state in which it is difficult to move the body or move quickly. |
|  | 2 |  | Rigidity | A state in which relaxing the limbs or body is difficult, indicating that the muscles are always tense and stiff. |
|  | 3 |  | Action tremor | A state in which one’s hands shake when holding or writing something or one’s legs shake when sitting cross-legged. |
|  | 4 |  | Rest tremor | A state in which one’s hands and legs shake while resting relaxed, unlike when shaking occurs when trying to hold or write something. |
|  | 5 |  | Postural instability | A state in which keeping one’s balance is difficult and one feels like falling over. |
|  | 6 |  | Gait disturbance (short-stepped gait) | A state in which taking the first step is difficult or one shuffles or takes small steps. |
|  | 7 |  | Freezing of gait | A state in which taking the first step is difficult or one stands rooted to the spot and is unable to take the first step. |
|  | 8 |  | Abnormal posture | A state in which one’s neck is lowered or has a hunchback or forward-leaning posture. |
|  | 9 |  | Salivation | A state in which one is unable to swallow saliva and drools. |
|  | 10 |  | Fall | A state in which one often falls over on a flat road or stairs or falls off a chair from a sitting position. |
|  | 11 |  | Dysphagia | A state in which one has difficulty swallowing food and chokes or spits it out. |
|  | 12 | I do not know. | | |

Q33. If you responded to Q32 and selected the symptom of parkinsonism that causes you the most distress, would you prefer to receive treatment for that symptom (selected in Q32)?

| 1  2  3 | Yes.  No.  I do not know. | Please circle only one item. |
| --- | --- | --- |

Q34. If you responded to Q32 and selected the symptom of parkinsonism that causes you the most distress, do you think you are being treated (receiving medication or advice from your physician) for that symptom (selected in Q32)?

| 1  2  3 | Yes.  No.  I do not know. | Please circle only one item. |
| --- | --- | --- |

Q35. If you responded to Q32 and selected the symptom that causes you the most distress, as you continue with treatment, which symptoms of parkinsonism would you prioritize for receiving treatment? Please select all applicable items.

|  |  |  | **Symptom** | **Explanation** |
| --- | --- | --- | --- | --- |
| You may circle multiple items. | 1 | Parkinsonism | Bradykinesia/  Akinesia | A state in which it is difficult to move the body or move quickly. |
|  | 2 |  | Rigidity | A state in which relaxing the limbs or body is difficult, indicating that the muscles are always tense and stiff. |
|  | 3 |  | Action tremor | A state in which one’s hands shake when holding or writing something or one’s legs shake when sitting cross-legged. |
|  | 4 |  | Rest tremor | A state in which one’s hands and legs shake while resting relaxed, unlike when shaking occurs when trying to hold or write something. |
|  | 5 |  | Postural instability | A state in which keeping one’s balance is difficult and one feels like falling over. |
|  | 6 |  | Gait disturbance (short-stepped gait) | A state in which taking the first step is difficult or one shuffles or takes small steps. |
|  | 7 |  | Freezing of gait | A state in which taking the first step is difficult or one stands rooted to the spot and is unable to take the first step. |
|  | 8 |  | Abnormal posture | A state in which one’s neck is lowered or has a hunchback or forward-leaning posture. |
|  | 9 |  | Salivation | A state in which one is unable to swallow saliva and drools. |
|  | 10 |  | Fall | A state in which one often falls over on a flat road or stairs or falls off a chair from a sitting position. |
|  | 11 |  | Dysphagia | A state in which one has difficulty swallowing food and chokes or spits it out. |
|  | 12 | I do not know. | | |

Q36. Please select the degree of satisfaction in terms of the treatment effect of the medication currently prescribed for cognitive impairment.

| 1  2  3  4  5  6  7 | I am extremely satisfied.  I am moderately satisfied.  It is difficult to select either.  I am slightly dissatisfied.  I am extremely dissatisfied.  I do not take it.  I do not know. | Please circle only one item. |
| --- | --- | --- |

Q37. Please select the degree of satisfaction in terms of the treatment effect of the medication currently prescribed for parkinsonism.

| 1  2  3  4  5  6  7 | I am extremely satisfied.  I am moderately satisfied.  It is difficult to select either.  I am slightly dissatisfied.  I am extremely dissatisfied.  I do not take it.  I do not know. | Please circle only one item. |
| --- | --- | --- |

Q38. Please select the degree of satisfaction in terms of the treatment effect of the medication currently prescribed for psychiatric symptoms.

| 1  2  3  4  5  6  7 | I am extremely satisfied.  I am moderately satisfied.  It is difficult to select either.  I am slightly dissatisfied.  I am extremely dissatisfied.  I do not take it.  I do not know. | Please circle only one item. |
| --- | --- | --- |

Q39. Please select the degree of satisfaction in terms of the treatment effect of the medication currently prescribed for sleep-related problems.

| 1  2  3  4  5  6  7 | I am extremely satisfied.  I am moderately satisfied.  It is difficult to select either.  I am slightly dissatisfied.  I am extremely dissatisfied.  I do not take it.  I do not know. | Please circle only one item. |
| --- | --- | --- |

Q40. Please select the degree of satisfaction in terms of the treatment effect of the medication currently prescribed for autonomic dysfunction.

| 1  2  3  4  5  6  7 | I am extremely satisfied.  I am moderately satisfied.  It is difficult to select either.  I am slightly dissatisfied.  I am extremely dissatisfied.  I do not take it.  I do not know. | Please circle only one item. |
| --- | --- | --- |

| **The questionnaire is complete.**  **Thank you for your cooperation.** |
| --- |
