# Supplementary material for: Treatment needs of dementia with Lewy bodies according to patients, caregivers, and physicians: a cross-sectional, observational, questionnaire-based study in Japan
Source: Alzheimers Res Ther. 2022 Dec 15;14:188. doi: 10.1186/s13195-022-01130-4 (PMC9751509; doi:10.1186/s13195-022-01130-4)
Supplement: Supplementary file 3 — Additional file 3: Supplementary Methods 2. Questionnaire for caregiver. [file 13195_2022_1130_MOESM3_ESM.docx]

| **Questionnaire for Caregiver**  Research on the Treatment Needs of Patients with  Dementia with Lewy bodies, their Caregivers, and their Physicians |
| --- |

| **Date of Completion of Questionnaire: Y M D**  **※Please fill in the date** |
| --- |

- Please complete this questionnaire at the earliest (within 3 weeks) from the day on which it was distributed.
- Approximately 25–30 min would be required to complete this questionnaire.

(The time required may vary slightly depending on the content of the responses.)

Please bear in mind the following when filling in the questionnaire:

- Please circle your response for each question. Please write a detailed answer in the space provided for questions where you are asked to make a written response.
- You may ask the patient questions and fill in the questionnaire on their behalf. However, in such a case, please first fill in your own questionnaire as caregiver before assisting the patient with their own questionnaire. If you fill in the questionnaire for the patient, please do not influence their answers.
- After completing, please check that you have not missed any questions.
- Please also check that no questions have been missed on the patient’s questionnaire.
- After checking, please insert both the caregiver and patient questionnaires into the letter pack provided to you at the hospital and post it in a mailbox.

| **[Final Confirmation] Please check the following when placing the questionnaires in the letter pack.**  □　I have placed the patient questionnaire in the letter pack  □　I have placed the caregiver questionnaire in the letter pack |
| --- |

| Questionnaire Help Desk  TEL: 0120-549-715  Available from 10:00 to 18:00 Monday to Friday  ＊Not available on Saturdays, Sundays or public holidays. |
| --- |

| For Company Use Only | Identification Code: ××-×× |
| --- | --- |

Symptoms of Dementia with Lewy Bodies (DLB)

|  | **Symptom domain** | **Symptom** | **Explanation** |
| --- | --- | --- | --- |
| 1 | Cognitive impairment | Memory impairment | A state in which one’s memory becomes extremely bad or one cannot recall things even with a hint. |
| 2 |  | Disorientation | A state in which one cannot tell what time of day it is or where they are. |
| 3 |  | Executive dysfunction | A state in which planning ahead to do something or to act in accordance with a procedure becomes difficult. |
| 4 |  | Attention dysfunction | A state in which one feels distracted or one’s attention or concentration is lost. |
| 5 |  | Fluctuating cognition | A state in which the cognitive function level varies from good to bad (stupor) and response levels fluctuate between lucid to reduced alertness. |
| 6 |  | Visuospatial dysfunction | A state in which one is unable to find something that is in front of them or is unable to accurately recognize something they see. |
| 7 |  | Other cognitive impairment | Other symptoms such as difficulty in speaking words (aphasia), difficulty in using items (apraxia), telling far-fetched stories (fabrication). |
| 8 | Parkinsonism | Bradykinesia/  Akinesia | A state in which it is difficult to move the body or move quickly. |
| 9 |  | Rigidity | A state in which relaxing the limbs or body is difficult, indicating that the muscles are always tense and stiff. |
| 10 |  | Action tremor | A state in which one’s hands shake when holding or writing something or one’s legs shake when sitting cross-legged. |
| 11 |  | Rest tremor | A state in which one’s hands and legs shake while resting relaxed, unlike when shaking occurs when trying to hold or write something. |
| 12 |  | Postural instability | A state in which keeping one’s balance is difficult and one feels like falling over. |
| 13 |  | Gait disturbance (short-stepped gait) | A state in which taking the first step is difficult or one shuffles or takes small steps. |
| 14 |  | Freezing of gait | A state in which taking the first step is difficult or one stands rooted to the spot and is unable to take the first step. |
| 15 |  | Abnormal posture | A state in which one’s neck is lowered or has a hunchback or forward-leaning posture. |
| 16 |  | Salivation | A state in which one is unable to swallow saliva and drools. |
| 17 |  | Fall | A state in which one often falls over on a flat road or stairs or falls off a chair from a sitting position. |
| 18 |  | Dysphagia | A state in which one has difficulty swallowing food and chokes or spits it out. |
| 19 | Psychiatric symptoms | Delusions | A state in which one falsely believes that their spouse is an imposter or is having an affair or that something was stolen. |
| 20 |  | Visual hallucinations | A state in which one sees something that does not really exist (person, animal, etc.). |
| 21 |  | Hallucinations other than visual hallucinations | A state in which one hears the voice of someone who is not present (auditory hallucination) or feels an illusory sensation and says something like “An insect is crawling under my skin” (cenesthesic hallucination). |
| 22 |  | Agitation/  Aggression | A state in which one has a heightened sense of emotion, speaks violent words, or resorts to violence. |
| 23 |  | Depression | A state in which one is depressed and has no motivation. |
| 24 |  | Anxiety | A state in which one feels restless or fidgety. |
| 25 |  | Apathy | A state in which one has lost interest in one’s surroundings and is unable to take voluntary action. |
| 26 |  | Disinhibition | A state in which one is unable to suppress one’s emotions or desires and uses speech and actions that are not acceptable in society. |
| 27 |  | Aberrant motor behavior | A state in which one engages in unusual behavior (e.g., wanders around or checks something excessively). |
| 28 |  | Negativism | A state in which one refuses everything that is offered, including going to day service (Refusal to eat should be classified as anorexia). |
| 29 |  | Delirium | A state in which one’s psychological state deteriorates owing to being in an environment that is different from normal, such as being in a hospital or being ill. |
| 30 |  | Other psychiatric symptom | Other symptoms such as being excessively dependent on caregivers (dependence) or excessively persistent (obsession). |

|  | **Symptom domain** | **Symptom** | **Explanation** |
| --- | --- | --- | --- |
| 31 | Eating behavior-related problems | Loss of appetite | A state in which one has little or no desire to eat. |
| 32 |  | Increase in appetite | A state in which one has a heightened desire to eat. |
| 33 |  | Weight loss | A state in which one loses weight. |
| 34 |  | Weight gain | A state in which one gains weight. |
| 35 |  | Food refusal | A state in which one says no to meals or refuses to eat even when being encouraged to eat. |
| 36 |  | Eating non-edible  things | A state in which one eats something that is not food. |
| 37 |  | Unbalanced diet | A state in which one is very picky about meals. |
| 38 | Sleep-related disorders | Rapid eye movement sleep behavior disorder | A state of sleep (dreaming) in which one talks in long sentences or talks as if in a conversation, yells, or does things like kicking and punching. |
| 39 |  | Daytime somnolence | A state in which one falls asleep during the daytime even after a good night’s sleep. |
| 40 |  | Day-night reversal | A state in which one stays awake during the night and sleeps during the day. |
| 41 |  | Night-time sleep disorder | A state in which one is unable to sleep well, wakes up many times at night, and wakes up early in the morning. |
| 42 |  | Sudden sleep | A state in which one suddenly falls asleep despite having been awake until that time. |
| 43 |  | Restless legs syndrome | A state in which one feels restless in the legs while sitting or lying down. |
| 44 |  | Periodic limb movement disorder | A state in which one or both arms or legs involuntarily move periodically and repeatedly at a set interval. |
| 45 | Autonomic dysfunction | Orthostatic hypotension | A state in which one feels like they are losing color or fainting, goes pale, or becomes dizzy when standing up from sitting or lying down. |
| 46 |  | Disturbance of  sweating | A state in which one sweats heavily only on the upper body or sweats only on the upper body despite feeling cold. |
| 47 |  | Constipation | A state in which one has poor bowel movements, which are sometimes accompanied by pain. |
| 48 |  | Night-time dysuria | Frequent urination (one gets up to go to the toilet three or more times during sleep), urinary incontinence, and a sensation of residual urine (one feels the urge to urinate even after urination). |
| 49 |  | Daytime dysuria | Frequent urination (one urinates eight or more times during the day), urinary incontinence, and a sensation of residual urine (one feels the urge to urinate even after urination). |
| 50 |  | Syncope | A state in which one experiences a temporary loss of consciousness but recovers after a few minutes (often observed after a meal or going to the toilet). |
| 51 |  | Dizziness | Dizziness is a term used to describe a range of sensations, such as feeling faint, woozy, weak or unsteady. |
| 52 | Sensory  disorders | Dysosmia | Dysosmia is a disorder described as any qualitative alteration or distortion of the perception of smell. |

Some of the initial questions will be about the patient.

Q1. How old is the patient?

| Age | years |
| --- | --- |

Q2. Is the patient male or female?

| 1  2 | Male  Female | Please circle only one item. |
| --- | --- | --- |

Q3. How many years of education did the patient have?

| 1  2 | (　　　　　　　　　) years  I do not know. | Please circle only one item. |
| --- | --- | --- |

Q4. What is the current level of care that the patient requires?

| 1  2  3  4  5  6  7  8 | Not applied for/application being processed  Requiring help 1  Requiring help 2  Long-term care level 1  Long-term care level 2  Long-term care level 3  Long-term care level 4  Long-term care level 5 | Please circle only  one item. |
| --- | --- | --- |

Q5. Where does the patient currently reside?

| 1  2  3 | Own home  Facility  Other (specifically: ) | Please circle only one item. |
| --- | --- | --- |

Q6. If you selected “Facility” in Q5, please specify the type of facility.

| 1  2  3  4  5  6 | Private nursing home  Group home (nursing home for patients with dementia)  Elderly housing (apartment with nursing service)  Please circle only one item.  Tokuyo Nursing Home  Roken Nursing Home (with rehabilitation service)  Other (specifically: ) |
| --- | --- |

Q7. If you selected “Own home” in Q5, please specify the number of individuals living with the patient (including the patient).

| 1  2  3 | One (living alone)  Please circle only one item.  Two  Three or more |
| --- | --- |

Q8. If you selected “Own home” in Q5, please specify whether the patient uses “Short stay.”

※“Short stay” refers to a nursing care service in which elderly individuals who live in their own homes can stay in a facility for a few days to receive meals, be bathed, or enjoy recreational activities.

| 1  2  3  4  5  6  7 | Twice a month or more  Once a month  Please circle only one item.  Once every 2 months  Once every 3 months  Frequency other than the above  Does not use  I do not know. |
| --- | --- |

Q9. If you selected “Own home” in Q5, please specify whether the patient uses “Long-term care (daytime service).”

※“Long-term care (daytime service)” refers to a nursing care service in which elderly individuals who live in their own homes can visit a facility during daytime to receive meals, be bathed, or enjoy recreational activities.

| 1  2  3  4  5  6  7  8 | Uses it more than 6 times a week  Uses it 5 times a week  Uses it 4 times a week  Uses it 3 times a week  Please circle only one item.  Uses it twice a week  Uses it once a week  Does not use  I do not know. |
| --- | --- |

Q10. If you selected “Own home” in Q5, please specify whether the patient uses “Outpatient rehabilitation (daytime care).”

※“Outpatient rehabilitation (daytime care)” refers to rehabilitation services (health management services) aimed at maintaining/improving mental and physical functions and supporting self-sustenance in daily lives of elderly people. Service users are elderly people living at home, who visit rehabilitation facilities (health facilities for the elderly, hospitals, clinics, etc.) without staying overnight.

| 1  2  3  4  5  6  7  8 | Uses it more than 6 times a week  Uses it 5 times a week  Uses it 4 times a week  Please circle only one item.  Uses it 3 times a week  Uses it twice a week  Uses it once a week  Does not use  I do not know. |
| --- | --- |

Q11. If you selected “Own home” in Q5, please specify whether the patient uses “small-scale, multifunctional home care.“

※“Small-scale, multifunctional home care“ is a service that provides a combination of day services, home care and short stays to enable people to continue living at home.

| 1  2  3 | Yes.  Please circle only one item.  No.  I do not know. |
| --- | --- |

Please tell us about yourself.

Q12. How old are you?

| Age | years |
| --- | --- |

Q13. Are you male or female?

| 1  2 | Male  Please circle only one item.  Female |
| --- | --- |

Q14. Are you currently employed?

| 1  2 | Yes.  Please circle only one item.  No. |
| --- | --- |

Q15. Do you live with the patient?

| 1  2 | Yes.  Please circle only one item.  No. |
| --- | --- |

Q16. How many hours do you spend with the patient each day?

| Time | Approximately( ) h |
| --- | --- |

Q17. What is your relationship with the patient?

| 1  2  3  4  5  6  7  8 | Spouse  Father or Mother  Sibling  Son or Daughter  Please circle only one item.  Son- or Daughter-in-law  Grandchild  Care provider  Other (specifically: ) |
| --- | --- |

Q18. If you did NOT select “Care provider” in Q17, do you have an assistant caregiver?

※“Assistant caregiver” refers to someone who assists you as a caregiver while providing care or who provides care in your stead and is not involved in the care industry.

| 1  2  3 | Yes.  Please circle only one item.  No.  I do not know. |
| --- | --- |

Q19. Please describe the patient.

Which was the first symptom that you observed in the patient? Please select one applicable symptom.

|  |  | **Symptom domain** | **Symptom** | **Explanation** |
| --- | --- | --- | --- | --- |
| Please circle only one item. | 1 | Cognitive impairment | Memory impairment | A state in which one’s memory becomes extremely bad or one cannot recall things even with a hint. |
|  | 2 |  | Disorientation | A state in which one cannot tell what time of day it is or where they are. |
|  | 3 |  | Executive dysfunction | A state in which planning ahead to do something or to act in accordance with a procedure becomes difficult. |
|  | 4 |  | Attention dysfunction | A state in which one feels distracted or one’s attention or concentration is lost. |
|  | 5 |  | Fluctuating cognition | A state in which the cognitive function level varies from good to bad (stupor) and response levels fluctuate between lucid to reduced alertness. |
|  | 6 |  | Visuospatial dysfunction | A state in which one is unable to find something that is in front of them or is unable to accurately recognize something they see. |
|  | 7 |  | Other cognitive impairment | Other symptoms such as difficulty in speaking words (aphasia), difficulty in using items (apraxia), telling far-fetched stories (fabrication). |
|  | 8 | Parkinsonism | Bradykinesia/  Akinesia | A state in which it is difficult to move the body or move quickly. |
|  | 9 |  | Rigidity | A state in which relaxing the limbs or body is difficult, indicating that the muscles are always tense and stiff. |
|  | 10 |  | Action tremor | A state in which one’s hands shake when holding or writing something or one’s legs shake when sitting cross-legged. |
|  | 11 |  | Rest tremor | A state in which one’s hands and legs shake while resting relaxed, unlike when shaking occurs when trying to hold or write something. |
|  | 12 |  | Postural instability | A state in which keeping one’s balance is difficult and one feels like falling over. |
|  | 13 |  | Gait disturbance (short-stepped gait) | A state in which taking the first step is difficult or one shuffles or takes small steps. |
|  | 14 |  | Freezing of gait | A state in which taking the first step is difficult or one stands rooted to the spot and is unable to take the first step. |
|  | 15 |  | Abnormal posture | A state in which one’s neck is lowered or has a hunchback or forward-leaning posture. |
|  | 16 |  | Salivation | A state in which one is unable to swallow saliva and drools. |
|  | 17 |  | Fall | A state in which one often falls over on a flat road or stairs or falls off a chair from a sitting position. |
|  | 18 |  | Dysphagia | A state in which one has difficulty swallowing food and chokes or spits it out. |
|  | 19 | Psychiatric symptoms | Delusions | A state in which one falsely believes that their spouse is an imposter or is having an affair or that something was stolen. |
|  | 20 |  | Visual hallucinations | A state in which one sees something that does not really exist (person, animal, etc.). |
|  | 21 |  | Hallucinations other than visual hallucinations | A state in which one hears the voice of someone who is not present (auditory hallucination) or feels an illusory sensation and says something like “An insect is crawling under my skin” (cenesthesic hallucination). |
|  | 22 |  | Agitation/Aggression | A state in which one has a heightened sense of emotion, speaks violent words, or resorts to violence. |
|  | 23 |  | Depression | A state in which one is depressed and has no motivation. |
|  | 24 |  | Anxiety | A state in which one feels restless or fidgety. |
|  | 25 |  | Apathy | A state in which one has lost interest in one’s surroundings and is unable to take voluntary action. |
|  | 26 |  | Disinhibition | A state in which one is unable to suppress one’s emotions or desires and uses speech and actions that are not acceptable in society. |
|  | 27 |  | Aberrant motor behavior | A state in which one engages in unusual behavior (e.g., wanders around or checks something excessively). |
|  | 28 |  | Negativism | A state in which one refuses everything that is offered, including going to day service (Refusal to eat should be classified as anorexia). |
|  | 29 |  | Delirium | A state in which one’s psychological state deteriorates owing to being in an environment that is different from normal, such as being in a hospital or being ill. |
|  | 30 |  | Other psychiatric symptom | Other symptoms such as being excessively dependent on caregivers (dependence) or excessively persistent (obsession). |

|  |  | **Symptom domain** | **Symptom** | **Explanation** |
| --- | --- | --- | --- | --- |
| Please circle only one item. | 31 | Eating behavior-related problems | Loss of appetite | A state in which one has little or no desire to eat. |
|  | 32 |  | Increase in appetite | A state in which one has a heightened desire to eat. |
|  | 33 |  | Weight loss | A state in which one loses weight. |
|  | 34 |  | Weight gain | A state in which one gains weight. |
|  | 35 |  | Food refusal | A state in which one says no to meals or refuses to eat even when being encouraged to eat. |
|  | 36 |  | Eating non-edible  things | A state in which one eats something that is not food. |
|  | 37 |  | Unbalanced diet | A state in which one is very picky about meals. |
|  | 38 | Sleep-related disorders | Rapid eye movement sleep behavior disorder | A state of sleep (dreaming) in which one talks in long sentences or talks as if in a conversation, yells, or does things like kicking and punching. |
|  | 39 |  | Daytime somnolence | A state in which one falls asleep during the daytime even after a good night’s sleep. |
|  | 40 |  | Day-night reversal | A state in which one stays awake during the night and sleeps during the day. |
|  | 41 |  | Night-time sleep disorder | A state in which one is unable to sleep well, wakes up many times at night, and wakes up early in the morning. |
|  | 42 |  | Sudden sleep | A state in which one suddenly falls asleep despite having been awake until that time. |
|  | 43 |  | Restless legs syndrome | A state in which one feels restless in the legs while sitting or lying down. |
|  | 44 |  | Periodic limb movement disorder | A state in which one or both arms or legs involuntarily move periodically and repeatedly at a set interval. |
|  | 45 | Autonomic dysfunction | Orthostatic hypotension | A state in which one feels like they are losing color or fainting, goes pale, or becomes dizzy when standing up from sitting or lying down. |
|  | 46 |  | Disturbance of  sweating | A state in which one sweats heavily only on the upper body or sweats only on the upper body despite feeling cold. |
|  | 47 |  | Constipation | A state in which one has poor bowel movements, which are sometimes accompanied by pain. |
|  | 48 |  | Night-time dysuria | Frequent urination (one gets up to go to the toilet three or more times during sleep), urinary incontinence, and a sensation of residual urine (one feels the urge to urinate even after urination). |
|  | 49 |  | Daytime dysuria | Frequent urination (one urinates eight or more times during the day), urinary incontinence, and a sensation of residual urine (one feels the urge to urinate even after urination). |
|  | 50 |  | Syncope | A state in which one experiences a temporary loss of consciousness but recovers after a few minutes (often observed after a meal or going to the toilet). |
|  | 51 |  | Dizziness | Dizziness is a term used to describe a range of sensations, such as feeling faint, woozy, weak or unsteady. |
|  | 52 | Sensory  disorders | Dysosmia | Dysosmia is a disorder described as any qualitative alteration or distortion of the perception of smell. |
|  | 53 | I do not know. | | |

Q20. If you were able to respond about the first symptom that appeared, how long ago did you observe that symptom? Please specify.

| 1  2  3  4  5  6 | Less than half a year  Between half a year and 1 year  Between 1 and 3 years  Please circle only one item.  Between 3 and 5 years  5 years or more  I do not know. |
| --- | --- |

Q21. How many years have passed since the patient was diagnosed with dementia with Lewy bodies (DLB)? Please specify the number of years. If it is under 1 year, please write the number of months.

| 1  2 | ( ) years ( ) months  Please circle only one item.  I do not know. |
| --- | --- |

Q22. What was the diagnosis of the patient before being diagnosed with DLB?

| 1  2  3  4  5  6  7 | The initial diagnosis was DLB.  Rapid eye movement (REM) sleep behavior disorder  Alzheimer’s disease  You may circle multiple items.  Depression  Parkinson’s disease  I do not know.  Other　(specifically: ) |
| --- | --- |

Q23. How often does the patient visit the hospital or clinic of the patient’s physician for DLB treatment?

| 1  2  ３４  ５ | Once every two or three weeks  Once a month  Please circle only one item.  Once every 2 months  Once every 3 months  Other (specifically: ) |
| --- | --- |

Q24. How often would you like the patient to visit the hospital or clinic of the patient’s physician?

| 1  2  ３４  ５ | Once every two or three weeks  Once a month  Please circle only one item.  Once every 2 months  Once every 3 months  Other (specifically: ) |
| --- | --- |

Q25. Please tell us about yourself.

Do you know a lot about dementia with Lewy bodies (DLB)?

| 1  2  3 | I know a lot about it.  Please circle only one item.  Neither yes nor no.  I do not know very much about it. |
| --- | --- |

Q26. Does the patient’s physician listen to what you say?

| 1  2  3  4  5 | The patient’s physician listens to me very well.  The patient’s physician listens to me.  Please circle only one item.  The patient’s physician sometimes listens to me.  The patient’s physician does not listen to me much.  The patient’s physician does not listen to me at all. |
| --- | --- |

Q27. Is there someone at the hospital or clinic other than the patient’s physician with whom you can talk?

| 1  2  3 | Yes.  Please circle only one item.  No.  I do not know. |
| --- | --- |

Q28. When prescribing medicine to the patient, does the patient's physician explain about it to you?

| 1  2  3  4 | The patient’s physician explains.  Please circle only one item.  The patient’s physician explains partially.  The patient’s physician does not explain.  I do not know. |
| --- | --- |

Q29. If you selected “The patient’s physician explains” or “The patient’s physician explains partially” in Q28, did you inform the patient about the explanation from the patient's physician?

| 1  2  3  4  5 | Yes.  Partially.  Please circle only one item.  No.  No, because I always hear it with the patient.  I do not know. |
| --- | --- |

Q30. Have you ever experienced an issue when the patient took medicine prescribed by the patient's physician?

| 1  2  3 | Yes.  Please circle only one item.  No.  I do not know. |
| --- | --- |

Q31. If you selected “Yes” in Q30, what issue did you experience when the patient took medicine? Please describe in detail.

| Details of the issue | ( ) |
| --- | --- |

Q32. If you selected “Yes” in Q30, did you inform the patient’s physician about the issue you experienced (Q31)?

| 1  2  3 | Yes.  Please circle only one item.  No.  I do not know. |
| --- | --- |

Q33. Is the patient taking medicine as instructed by the patient’s physician or pharmacist?

| 1  2  3 | Yes.  Please circle only one item.  No.  I do not know. |
| --- | --- |

Q34. If you selected “No” (the patient is not taking medicine as instructed) in Q33, what is the reason for not taking medicine as instructed or for not being able to take it properly?

| 1  2  3  4  5  6 | The patient forgot to take it.  You may circle  multiple items.  The patient has difficulties swallowing it  It does not work.  The patient is worried that a side effect may occur in the future.  I do not know.  Other reason (specifically: ) |
| --- | --- |

Q35. If you selected “The patient forgot to take it” in Q34, which medicine did the patient forget to take or was the patient unable to take properly?

| 1  2 | Medicine name: (specifically: 　　 )  Please circle only one item.  I do not know. |
| --- | --- |

| Please respond to the following questions based on what you believe (feel).  (Q36–Q41) |
| --- |

| Please proceed to the following page ▶▶▶ |
| --- |

Q36. Please select only one symptom of the patient that currently causes you the most distress.

|  |  | **Symptom domain** | **Symptom** | **Explanation** |
| --- | --- | --- | --- | --- |
| Please circle only one item. | 1 | Cognitive impairment | Memory impairment | A state in which one’s memory becomes extremely bad or one cannot recall things even with a hint. |
|  | 2 |  | Disorientation | A state in which one cannot tell what time of day it is or where they are. |
|  | 3 |  | Executive dysfunction | A state in which planning ahead to do something or to act in accordance with a procedure becomes difficult. |
|  | 4 |  | Attention dysfunction | A state in which one feels distracted or one’s attention or concentration is lost. |
|  | 5 |  | Fluctuating cognition | A state in which the cognitive function level varies from good to bad (stupor) and response levels fluctuate between lucid to reduced alertness. |
|  | 6 |  | Visuospatial dysfunction | A state in which one is unable to find something that is in front of them or is unable to accurately recognize something they see. |
|  | 7 |  | Other cognitive impairment | Other symptoms such as difficulty in speaking words (aphasia), difficulty in using items (apraxia), telling far-fetched stories (fabrication). |
|  | 8 | Parkinsonism | Bradykinesia/  Akinesia | A state in which it is difficult to move the body or move quickly. |
|  | 9 |  | Rigidity | A state in which relaxing the limbs or body is difficult, indicating that the muscles are always tense and stiff. |
|  | 10 |  | Action tremor | A state in which one’s hands shake when holding or writing something or one’s legs shake when sitting cross-legged. |
|  | 11 |  | Rest tremor | A state in which one’s hands and legs shake while resting relaxed, unlike when shaking occurs when trying to hold or write something. |
|  | 12 |  | Postural instability | A state in which keeping one’s balance is difficult and one feels like falling over. |
|  | 13 |  | Gait disturbance (short-stepped gait) | A state in which taking the first step is difficult or one shuffles or takes small steps. |
|  | 14 |  | Freezing of gait | A state in which taking the first step is difficult or one stands rooted to the spot and is unable to take the first step. |
|  | 15 |  | Abnormal posture | A state in which one’s neck is lowered or has a hunchback or forward-leaning posture. |
|  | 16 |  | Salivation | A state in which one is unable to swallow saliva and drools. |
|  | 17 |  | Fall | A state in which one often falls over on a flat road or stairs or falls off a chair from a sitting position. |
|  | 18 |  | Dysphagia | A state in which one has difficulty swallowing food and chokes or spits it out. |
|  | 19 | Psychiatric symptoms | Delusions | A state in which one falsely believes that their spouse is an imposter or is having an affair or that something was stolen. |
|  | 20 |  | Visual hallucinations | A state in which one sees something that does not really exist (person, animal, etc.). |
|  | 21 |  | Hallucinations other than visual hallucinations | A state in which one hears the voice of someone who is not present (auditory hallucination) or feels an illusory sensation and says something like “An insect is crawling under my skin” (cenesthesic hallucination). |
|  | 22 |  | Agitation/  Aggression | A state in which one has a heightened sense of emotion, speaks violent words, or resorts to violence. |
|  | 23 |  | Depression | A state in which one is depressed and has no motivation. |
|  | 24 |  | Anxiety | A state in which one feels restless or fidgety. |
|  | 25 |  | Apathy | A state in which one has lost interest in one’s surroundings and is unable to take voluntary action. |
|  | 26 |  | Disinhibition | A state in which one is unable to suppress one’s emotions or desires and uses speech and actions that are not acceptable in society. |
|  | 27 |  | Aberrant motor behavior | A state in which one engages in unusual behavior (e.g., wanders around or checks something excessively). |
|  | 28 |  | Negativism | A state in which one refuses everything that is offered, including going to day service (Refusal to eat should be classified as anorexia). |
|  | 29 |  | Delirium | A state in which one’s psychological state deteriorates owing to being in an environment that is different from normal, such as being in a hospital or being ill. |
|  | 30 |  | Other psychiatric symptom | Other symptoms such as being excessively dependent on caregivers (dependence) or excessively persistent (obsession). |

|  |  | **Symptom domain** | **Symptom** | **Explanation** |
| --- | --- | --- | --- | --- |
| Please circle only one item. | 31 | Eating behavior-related problems | Loss of appetite | A state in which one has little or no desire to eat. |
|  | 32 |  | Increase in appetite | A state in which one has a heightened desire to eat. |
|  | 33 |  | Weight loss | A state in which one loses weight. |
|  | 34 |  | Weight gain | A state in which one gains weight. |
|  | 35 |  | Food refusal | A state in which one says no to meals or refuses to eat even when being encouraged to eat. |
|  | 36 |  | Eating non-edible  things | A state in which one eats something that is not food. |
|  | 37 |  | Unbalanced diet | A state in which one is very picky about meals. |
|  | 38 | Sleep-related disorders | Rapid eye movement sleep behavior disorder | A state of sleep (dreaming) in which one talks in long sentences or talks as if in a conversation, yells, or does things like kicking and punching. |
|  | 39 |  | Daytime somnolence | A state in which one falls asleep during the daytime even after a good night’s sleep. |
|  | 40 |  | Day-night reversal | A state in which one stays awake during the night and sleeps during the day. |
|  | 41 |  | Night-time sleep disorder | A state in which one is unable to sleep well, wakes up many times at night, and wakes up early in the morning. |
|  | 42 |  | Sudden sleep | A state in which one suddenly falls asleep despite having been awake until that time. |
|  | 43 |  | Restless legs syndrome | A state in which one feels restless in the legs while sitting or lying down. |
|  | 44 |  | Periodic limb movement disorder | A state in which one or both arms or legs involuntarily move periodically and repeatedly at a set interval. |
|  | 45 | Autonomic dysfunction | Orthostatic hypotension | A state in which one feels like they are losing color or fainting, goes pale, or becomes dizzy when standing up from sitting or lying down. |
|  | 46 |  | Disturbance of  sweating | A state in which one sweats heavily only on the upper body or sweats only on the upper body despite feeling cold. |
|  | 47 |  | Constipation | A state in which one has poor bowel movements, which are sometimes accompanied by pain. |
|  | 48 |  | Night-time dysuria | Frequent urination (one gets up to go to the toilet three or more times during sleep), urinary incontinence, and a sensation of residual urine (one feels the urge to urinate even after urination). |
|  | 49 |  | Daytime dysuria | Frequent urination (one urinates eight or more times during the day), urinary incontinence, and a sensation of residual urine (one feels the urge to urinate even after urination). |
|  | 50 |  | Syncope | A state in which one experiences a temporary loss of consciousness but recovers after a few minutes (often observed after a meal or going to the toilet). |
|  | 51 |  | Dizziness | Dizziness is a term used to describe a range of sensations, such as feeling faint, woozy, weak or unsteady. |
|  | 52 | Sensory  disorders | Dysosmia | Dysosmia is a disorder described as any qualitative alteration or distortion of the perception of smell. |
|  | 53 | I do not know. | | |

Q37. If you responded to Q36 and selected the patient’s symptom that causes you the most distress, what was your reason for selecting that symptom (selected in Q36)?

| Reason |  |
| --- | --- |

Q38. If you responded to Q36 and selected the patient’s symptom that causes you the most distress, have you informed the patient’s physician about that symptom (selected in Q36)?

| 1  2  3 | Yes.  Please circle only one item.  No.  I do not know. |
| --- | --- |

Q39. If you responded to Q36 and selected the patient’s symptom that causes you the most distress, has the patient’s physician inquired about that symptom (selected in Q36)?

| 1  2  3 | Yes.  Please circle only one item.  No.  I do not know. |
| --- | --- |

| Please proceed to the following page ▶▶▶ |
| --- |

Q40. Please select all of the patient's other symptoms other than the symptom that currently causes you the most distress.

|  |  | **Symptom domain** | **Symptom** | **Explanation** |
| --- | --- | --- | --- | --- |
| You may circle multiple items. | 1 | Cognitive impairment | Memory impairment | A state in which one’s memory becomes extremely bad or one cannot recall things even with a hint. |
|  | 2 |  | Disorientation | A state in which one cannot tell what time of day it is or where they are. |
|  | 3 |  | Executive dysfunction | A state in which planning ahead to do something or to act in accordance with a procedure becomes difficult. |
|  | 4 |  | Attention dysfunction | A state in which one feels distracted or one’s attention or concentration is lost. |
|  | 5 |  | Fluctuating cognition | A state in which the cognitive function level varies from good to bad (stupor) and response levels fluctuate between lucid to reduced alertness. |
|  | 6 |  | Visuospatial dysfunction | A state in which one is unable to find something that is in front of them or is unable to accurately recognize something they see. |
|  | 7 |  | Other cognitive impairment | Other symptoms such as difficulty in speaking words (aphasia), difficulty in using items (apraxia), telling far-fetched stories (fabrication). |
|  | 8 | Parkinsonism | Bradykinesia/  Akinesia | A state in which it is difficult to move the body or move quickly. |
|  | 9 |  | Rigidity | A state in which relaxing the limbs or body is difficult, indicating that the muscles are always tense and stiff. |
|  | 10 |  | Action tremor | A state in which one’s hands shake when holding or writing something or one’s legs shake when sitting cross-legged. |
|  | 11 |  | Rest tremor | A state in which one’s hands and legs shake while resting relaxed, unlike when shaking occurs when trying to hold or write something. |
|  | 12 |  | Postural instability | A state in which keeping one’s balance is difficult and one feels like falling over. |
|  | 13 |  | Gait disturbance (short-stepped gait) | A state in which taking the first step is difficult or one shuffles or takes small steps. |
|  | 14 |  | Freezing of gait | A state in which taking the first step is difficult or one stands rooted to the spot and is unable to take the first step. |
|  | 15 |  | Abnormal posture | A state in which one’s neck is lowered or has a hunchback or forward-leaning posture. |
|  | 16 |  | Salivation | A state in which one is unable to swallow saliva and drools. |
|  | 17 |  | Fall | A state in which one often falls over on a flat road or stairs or falls off a chair from a sitting position. |
|  | 18 |  | Dysphagia | A state in which one has difficulty swallowing food and chokes or spits it out. |
|  | 19 | Psychiatric symptoms | Delusions | A state in which one falsely believes that their spouse is an imposter or is having an affair or that something was stolen. |
|  | 20 |  | Visual hallucinations | A state in which one sees something that does not really exist (person, animal, etc.). |
|  | 21 |  | Hallucinations other than visual hallucinations | A state in which one hears the voice of someone who is not present (auditory hallucination) or feels an illusory sensation and says something like “An insect is crawling under my skin” (cenesthesic hallucination). |
|  | 22 |  | Agitation/  Aggression | A state in which one has a heightened sense of emotion, speaks violent words, or resorts to violence. |
|  | 23 |  | Depression | A state in which one is depressed and has no motivation. |
|  | 24 |  | Anxiety | A state in which one feels restless or fidgety. |
|  | 25 |  | Apathy | A state in which one has lost interest in one’s surroundings and is unable to take voluntary action. |
|  | 26 |  | Disinhibition | A state in which one is unable to suppress one’s emotions or desires and uses speech and actions that are not acceptable in society. |
|  | 27 |  | Aberrant motor behavior | A state in which one engages in unusual behavior (e.g., wanders around or checks something excessively). |
|  | 28 |  | Negativism | A state in which one refuses everything that is offered, including going to day service (Refusal to eat should be classified as anorexia). |
|  | 29 |  | Delirium | A state in which one’s psychological state deteriorates owing to being in an environment that is different from normal, such as being in a hospital or being ill. |
|  | 30 |  | Other psychiatric symptom | Other symptoms such as being excessively dependent on caregivers (dependence) or excessively persistent (obsession). |

|  |  | **Symptom domain** | **Symptom** | **Explanation** |
| --- | --- | --- | --- | --- |
| You may circle multiple items. | 31 | Eating behavior-related problems | Loss of appetite | A state in which one has little or no desire to eat. |
|  | 32 |  | Increase in appetite | A state in which one has a heightened desire to eat. |
|  | 33 |  | Weight loss | A state in which one loses weight. |
|  | 34 |  | Weight gain | A state in which one gains weight. |
|  | 35 |  | Food refusal | A state in which one says no to meals or refuses to eat even when being encouraged to eat. |
|  | 36 |  | Eating non-edible  things | A state in which one eats something that is not food. |
|  | 37 |  | Unbalanced diet | A state in which one is very picky about meals. |
|  | 38 | Sleep-related disorders | Rapid eye movement sleep behavior disorder | A state of sleep (dreaming) in which one talks in long sentences or talks as if in a conversation, yells, or does things like kicking and punching. |
|  | 39 |  | Daytime somnolence | A state in which one falls asleep during the daytime even after a good night’s sleep. |
|  | 40 |  | Day-night reversal | A state in which one stays awake during the night and sleeps during the day. |
|  | 41 |  | Night-time sleep disorder | A state in which one is unable to sleep well, wakes up many times at night, and wakes up early in the morning. |
|  | 42 |  | Sudden sleep | A state in which one suddenly falls asleep despite having been awake until that time. |
|  | 43 |  | Restless legs syndrome | A state in which one feels restless in the legs while sitting or lying down. |
|  | 44 |  | Periodic limb movement disorder | A state in which one or both arms or legs involuntarily move periodically and repeatedly at a set interval. |
|  | 45 | Autonomic dysfunction | Orthostatic hypotension | A state in which one feels like they are losing color or fainting, goes pale, or becomes dizzy when standing up from sitting or lying down. |
|  | 46 |  | Disturbance of  sweating | A state in which one sweats heavily only on the upper body or sweats only on the upper body despite feeling cold. |
|  | 47 |  | Constipation | A state in which one has poor bowel movements, which are sometimes accompanied by pain. |
|  | 48 |  | Night-time dysuria | Frequent urination (one gets up to go to the toilet three or more times during sleep), urinary incontinence, and a sensation of residual urine (one feels the urge to urinate even after urination). |
|  | 49 |  | Daytime dysuria | Frequent urination (one urinates eight or more times during the day), urinary incontinence, and a sensation of residual urine (one feels the urge to urinate even after urination). |
|  | 50 |  | Syncope | A state in which one experiences a temporary loss of consciousness but recovers after a few minutes (often observed after a meal or going to the toilet). |
|  | 51 |  | Dizziness | Dizziness is a term used to describe a range of sensations, such as feeling faint, woozy, weak or unsteady. |
|  | 52 | Sensory  disorders | Dysosmia | Dysosmia is a disorder described as any qualitative alteration or distortion of the perception of smell. |
|  | 53 | No other troubling symptoms. | | |
|  | 54 | I do not know. | | |

Q41. As the patient continues with treatment, which patient’s symptom would you most likely prioritize for receiving treatment? Please select only one.

|  |  | **Symptom domain** | **Symptom** | **Explanation** |
| --- | --- | --- | --- | --- |
| Please circle only one item. | 1 | Cognitive impairment | Memory impairment | A state in which one’s memory becomes extremely bad or one cannot recall things even with a hint. |
|  | 2 |  | Disorientation | A state in which one cannot tell what time of day it is or where they are. |
|  | 3 |  | Executive dysfunction | A state in which planning ahead to do something or to act in accordance with a procedure becomes difficult. |
|  | 4 |  | Attention dysfunction | A state in which one feels distracted or one’s attention or concentration is lost. |
|  | 5 |  | Fluctuating cognition | A state in which the cognitive function level varies from good to bad (stupor) and response levels fluctuate between lucid to reduced alertness. |
|  | 6 |  | Visuospatial dysfunction | A state in which one is unable to find something that is in front of them or is unable to accurately recognize something they see. |
|  | 7 |  | Other cognitive impairment | Other symptoms such as difficulty in speaking words (aphasia), difficulty in using items (apraxia), telling far-fetched stories (fabrication). |
|  | 8 | Parkinsonism | Bradykinesia/  Akinesia | A state in which it is difficult to move the body or move quickly. |
|  | 9 |  | Rigidity | A state in which relaxing the limbs or body is difficult, indicating that the muscles are always tense and stiff. |
|  | 10 |  | Action tremor | A state in which one’s hands shake when holding or writing something or one’s legs shake when sitting cross-legged. |
|  | 11 |  | Rest tremor | A state in which one’s hands and legs shake while resting relaxed, unlike when shaking occurs when trying to hold or write something. |
|  | 12 |  | Postural instability | A state in which keeping one’s balance is difficult and one feels like falling over. |
|  | 13 |  | Gait disturbance (short-stepped gait) | A state in which taking the first step is difficult or one shuffles or takes small steps. |
|  | 14 |  | Freezing of gait | A state in which taking the first step is difficult or one stands rooted to the spot and is unable to take the first step. |
|  | 15 |  | Abnormal posture | A state in which one’s neck is lowered or has a hunchback or forward-leaning posture. |
|  | 16 |  | Salivation | A state in which one is unable to swallow saliva and drools. |
|  | 17 |  | Fall | A state in which one often falls over on a flat road or stairs or falls off a chair from a sitting position. |
|  | 18 |  | Dysphagia | A state in which one has difficulty swallowing food and chokes or spits it out. |
|  | 19 | Psychiatric symptoms | Delusions | A state in which one falsely believes that their spouse is an imposter or is having an affair or that something was stolen. |
|  | 20 |  | Visual hallucinations | A state in which one sees something that does not really exist (person, animal, etc.). |
|  | 21 |  | Hallucinations other than visual hallucinations | A state in which one hears the voice of someone who is not present (auditory hallucination) or feels an illusory sensation and says something like “An insect is crawling under my skin” (cenesthesic hallucination). |
|  | 22 |  | Agitation/  Aggression | A state in which one has a heightened sense of emotion, speaks violent words, or resorts to violence. |
|  | 23 |  | Depression | A state in which one is depressed and has no motivation. |
|  | 24 |  | Anxiety | A state in which one feels restless or fidgety. |
|  | 25 |  | Apathy | A state in which one has lost interest in one’s surroundings and is unable to take voluntary action. |
|  | 26 |  | Disinhibition | A state in which one is unable to suppress one’s emotions or desires and uses speech and actions that are not acceptable in society. |
|  | 27 |  | Aberrant motor behavior | A state in which one engages in unusual behavior (e.g., wanders around or checks something excessively). |
|  | 28 |  | Negativism | A state in which one refuses everything that is offered, including going to day service (Refusal to eat should be classified as anorexia). |
|  | 29 |  | Delirium | A state in which one’s psychological state deteriorates owing to being in an environment that is different from normal, such as being in a hospital or being ill. |
|  | 30 |  | Other psychiatric symptom | Other symptoms such as being excessively dependent on caregivers (dependence) or excessively persistent (obsession). |

|  |  | **Symptom domain** | **Symptom** | **Explanation** |
| --- | --- | --- | --- | --- |
| Please circle only one item. | 31 | Eating behavior-related problems | Loss of appetite | A state in which one has little or no desire to eat. |
|  | 32 |  | Increase in appetite | A state in which one has a heightened desire to eat. |
|  | 33 |  | Weight loss | A state in which one loses weight. |
|  | 34 |  | Weight gain | A state in which one gains weight. |
|  | 35 |  | Food refusal | A state in which one says no to meals or refuses to eat even when being encouraged to eat. |
|  | 36 |  | Eating non-edible  things | A state in which one eats something that is not food. |
|  | 37 |  | Unbalanced diet | A state in which one is very picky about meals. |
|  | 38 | Sleep-related disorders | Rapid eye movement sleep behavior disorder | A state of sleep (dreaming) in which one talks in long sentences or talks as if in a conversation, yells, or does things like kicking and punching. |
|  | 39 |  | Daytime somnolence | A state in which one falls asleep during the daytime even after a good night’s sleep. |
|  | 40 |  | Day-night reversal | A state in which one stays awake during the night and sleeps during the day. |
|  | 41 |  | Night-time sleep disorder | A state in which one is unable to sleep well, wakes up many times at night, and wakes up early in the morning. |
|  | 42 |  | Sudden sleep | A state in which one suddenly falls asleep despite having been awake until that time. |
|  | 43 |  | Restless legs syndrome | A state in which one feels restless in the legs while sitting or lying down. |
|  | 44 |  | Periodic limb movement disorder | A state in which one or both arms or legs involuntarily move periodically and repeatedly at a set interval. |
|  | 45 | Autonomic dysfunction | Orthostatic hypotension | A state in which one feels like they are losing color or fainting, goes pale, or becomes dizzy when standing up from sitting or lying down. |
|  | 46 |  | Disturbance of  sweating | A state in which one sweats heavily only on the upper body or sweats only on the upper body despite feeling cold. |
|  | 47 |  | Constipation | A state in which one has poor bowel movements, which are sometimes accompanied by pain. |
|  | 48 |  | Night-time dysuria | Frequent urination (one gets up to go to the toilet three or more times during sleep), urinary incontinence, and a sensation of residual urine (one feels the urge to urinate even after urination). |
|  | 49 |  | Daytime dysuria | Frequent urination (one urinates eight or more times during the day), urinary incontinence, and a sensation of residual urine (one feels the urge to urinate even after urination). |
|  | 50 |  | Syncope | A state in which one experiences a temporary loss of consciousness but recovers after a few minutes (often observed after a meal or going to the toilet). |
|  | 51 |  | Dizziness | Dizziness is a term used to describe a range of sensations, such as feeling faint, woozy, weak or unsteady. |
|  | 52 | Sensory  disorders | Dysosmia | Dysosmia is a disorder described as any qualitative alteration or distortion of the perception of smell. |
|  | 53 | I do not know. | | |

| Please consider the patient and respond to the following questions without discussing them with the patient.  (Q42–Q52) |
| --- |

Q42. Do you think that the patient has some issue with bowel movements?

| 1  2  3 | Yes.  Please circle only one item.  No.  I do not know. |
| --- | --- |

| Please proceed to the following page ▶▶▶ |
| --- |

Q43. Please select all of the symptoms that currently apply to the patient.

|  |  | **Symptom domain** | **Symptom** | **Explanation** |
| --- | --- | --- | --- | --- |
| You may circle multiple items. | 1 | Cognitive impairment | Memory impairment | A state in which one’s memory becomes extremely bad or one cannot recall things even with a hint. |
|  | 2 |  | Disorientation | A state in which one cannot tell what time of day it is or where they are. |
|  | 3 |  | Executive dysfunction | A state in which planning ahead to do something or to act in accordance with a procedure becomes difficult. |
|  | 4 |  | Attention dysfunction | A state in which one feels distracted or one’s attention or concentration is lost. |
|  | 5 |  | Fluctuating cognition | A state in which the cognitive function level varies from good to bad (stupor) and response levels fluctuate between lucid to reduced alertness. |
|  | 6 |  | Visuospatial dysfunction | A state in which one is unable to find something that is in front of them or is unable to accurately recognize something they see. |
|  | 7 |  | Other cognitive impairment | Other symptoms such as difficulty in speaking words (aphasia), difficulty in using items (apraxia), telling far-fetched stories (fabrication). |
|  | 8 | Parkinsonism | Bradykinesia/  Akinesia | A state in which it is difficult to move the body or move quickly. |
|  | 9 |  | Rigidity | A state in which relaxing the limbs or body is difficult, indicating that the muscles are always tense and stiff. |
|  | 10 |  | Action tremor | A state in which one’s hands shake when holding or writing something or one’s legs shake when sitting cross-legged. |
|  | 11 |  | Rest tremor | A state in which one’s hands and legs shake while resting relaxed, unlike when shaking occurs when trying to hold or write something. |
|  | 12 |  | Postural instability | A state in which keeping one’s balance is difficult and one feels like falling over. |
|  | 13 |  | Gait disturbance (short-stepped gait) | A state in which taking the first step is difficult or one shuffles or takes small steps. |
|  | 14 |  | Freezing of gait | A state in which taking the first step is difficult or one stands rooted to the spot and is unable to take the first step. |
|  | 15 |  | Abnormal posture | A state in which one’s neck is lowered or has a hunchback or forward-leaning posture. |
|  | 16 |  | Salivation | A state in which one is unable to swallow saliva and drools. |
|  | 17 |  | Fall | A state in which one often falls over on a flat road or stairs or falls off a chair from a sitting position. |
|  | 18 |  | Dysphagia | A state in which one has difficulty swallowing food and chokes or spits it out. |
|  | 19 | Psychiatric symptoms | Delusions | A state in which one falsely believes that their spouse is an imposter or is having an affair or that something was stolen. |
|  | 20 |  | Visual hallucination | A state in which one sees something that does not really exist (person, animal, etc.). |
|  | 21 |  | Hallucinations other than visual hallucinations | A state in which one hears the voice of someone who is not present (auditory hallucination) or feels an illusory sensation and says something like “An insect is crawling under my skin” (cenesthesic hallucination). |
|  | 22 |  | Agitation/  Aggression | A state in which one has a heightened sense of emotion, speaks violent words, or resorts to violence. |
|  | 23 |  | Depression | A state in which one is depressed and has no motivation. |
|  | 24 |  | Anxiety | A state in which one feels restless or fidgety. |
|  | 25 |  | Apathy | A state in which one has lost interest in one’s surroundings and is unable to take voluntary action. |
|  | 26 |  | Disinhibition | A state in which one is unable to suppress one’s emotions or desires and uses speech and actions that are not acceptable in society. |
|  | 27 |  | Aberrant motor behavior | A state in which one engages in unusual behavior (e.g., wanders around or checks something excessively). |
|  | 28 |  | Negativism | A state in which one refuses everything that is offered, including going to day service (Refusal to eat should be classified as anorexia). |
|  | 29 |  | Delirium | A state in which one’s psychological state deteriorates owing to being in an environment that is different from normal, such as being in a hospital or being ill. |
|  | 30 |  | Other psychiatric symptom | Other symptoms such as being excessively dependent on caregivers (dependence) or excessively persistent (obsession). |

|  |  | **Symptom domain** | **Symptom** | **Explanation** |
| --- | --- | --- | --- | --- |
| You may circle multiple items. | 31 | Eating behavior-related problems | Loss of appetite | A state in which one has little or no desire to eat. |
|  | 32 |  | Increase in appetite | A state in which one has a heightened desire to eat. |
|  | 33 |  | Weight loss | A state in which one loses weight. |
|  | 34 |  | Weight gain | A state in which one gains weight. |
|  | 35 |  | Food refusal | A state in which one says no to meals or refuses to eat even when being encouraged to eat. |
|  | 36 |  | Eating non-edible  things | A state in which one eats something that is not food. |
|  | 37 |  | Unbalanced diet | A state in which one is very picky about meals. |
|  | 38 | Sleep-related disorders | Rapid eye movement sleep behavior disorder | A state of sleep (dreaming) in which one talks in long sentences or talks as if in a conversation, yells, or does things like kicking and punching. |
|  | 39 |  | Daytime somnolence | A state in which one falls asleep during the daytime even after a good night’s sleep. |
|  | 40 |  | Day-night reversal | A state in which one stays awake during the night and sleeps during the day. |
|  | 41 |  | Night-time sleep disorder | A state in which one is unable to sleep well, wakes up many times at night, and wakes up early in the morning. |
|  | 42 |  | Sudden sleep | A state in which one suddenly falls asleep despite having been awake until that time. |
|  | 43 |  | Restless legs syndrome | A state in which one feels restless in the legs while sitting or lying down. |
|  | 44 |  | Periodic limb movement disorder | A state in which one or both arms or legs involuntarily move periodically and repeatedly at a set interval. |
|  | 45 | Autonomic dysfunction | Orthostatic hypotension | A state in which one feels like they are losing color or fainting, goes pale, or becomes dizzy when standing up from sitting or lying down. |
|  | 46 |  | Disturbance of  sweating | A state in which one sweats heavily only on the upper body or sweats only on the upper body despite feeling cold. |
|  | 47 |  | Constipation | A state in which one has poor bowel movements, which are sometimes accompanied by pain. |
|  | 48 |  | Night-time dysuria | Frequent urination (one gets up to go to the toilet three or more times during sleep), urinary incontinence, and a sensation of residual urine (one feels the urge to urinate even after urination). |
|  | 49 |  | Daytime dysuria | Frequent urination (one urinates eight or more times during the day), urinary incontinence, and a sensation of residual urine (one feels the urge to urinate even after urination). |
|  | 50 |  | Syncope | A state in which one experiences a temporary loss of consciousness but recovers after a few minutes (often observed after a meal or going to the toilet). |
|  | 51 |  | Dizziness | Dizziness is a term used to describe a range of sensations, such as feeling faint, woozy, weak or unsteady. |
|  | 52 | Sensory  disorders | Dysosmia | Dysosmia is a disorder described as any qualitative alteration or distortion of the perception of smell. |
|  | 53 | I do not know. | | |

Q44. Please select all of the patient’s symptoms for which you think the patient is currently receiving treatment.

|  |  | **Symptom domain** | **Symptom** | **Explanation** |
| --- | --- | --- | --- | --- |
| You may circle multiple items. | 1 | Cognitive impairment | Memory impairment | A state in which one’s memory becomes extremely bad or one cannot recall things even with a hint. |
|  | 2 |  | Disorientation | A state in which one cannot tell what time of day it is or where they are. |
|  | 3 |  | Executive dysfunction | A state in which planning ahead to do something or to act in accordance with a procedure becomes difficult. |
|  | 4 |  | Attention dysfunction | A state in which one feels distracted or one’s attention or concentration is lost. |
|  | 5 |  | Fluctuating cognition | A state in which the cognitive function level varies from good to bad (stupor) and response levels fluctuate between lucid to reduced alertness. |
|  | 6 |  | Visuospatial dysfunction | A state in which one is unable to find something that is in front of them or is unable to accurately recognize something they see. |
|  | 7 |  | Other cognitive impairment | Other symptoms such as difficulty in speaking words (aphasia), difficulty in using items (apraxia), telling far-fetched stories (fabrication). |
|  | 8 | Parkinsonism | Bradykinesia/  Akinesia | A state in which it is difficult to move the body or move quickly. |
|  | 9 |  | Rigidity | A state in which relaxing the limbs or body is difficult, indicating that the muscles are always tense and stiff. |
|  | 10 |  | Action tremor | A state in which one’s hands shake when holding or writing something or one’s legs shake when sitting cross-legged. |
|  | 11 |  | Rest tremor | A state in which one’s hands and legs shake while resting relaxed, unlike when shaking occurs when trying to hold or write something. |
|  | 12 |  | Postural instability | A state in which keeping one’s balance is difficult and one feels like falling over. |
|  | 13 |  | Gait disturbance (short-stepped gait) | A state in which taking the first step is difficult or one shuffles or takes small steps. |
|  | 14 |  | Freezing of gait | A state in which taking the first step is difficult or one stands rooted to the spot and is unable to take the first step. |
|  | 15 |  | Abnormal posture | A state in which one’s neck is lowered or has a hunchback or forward-leaning posture. |
|  | 16 |  | Salivation | A state in which one is unable to swallow saliva and drools. |
|  | 17 |  | Fall | A state in which one often falls over on a flat road or stairs or falls off a chair from a sitting position. |
|  | 18 |  | Dysphagia | A state in which one has difficulty swallowing food and chokes or spits it out. |
|  | 19 | Psychiatric symptoms | Delusions | A state in which one falsely believes that their spouse is an imposter or is having an affair or that something was stolen. |
|  | 20 |  | Visual hallucinations | A state in which one sees something that does not really exist (person, animal, etc.). |
|  | 21 |  | Hallucinations other than visual hallucinations | A state in which one hears the voice of someone who is not present (auditory hallucination) or feels an illusory sensation and says something like “An insect is crawling under my skin” (cenesthesic hallucination). |
|  | 22 |  | Agitation/  Aggression | A state in which one has a heightened sense of emotion, speaks violent words, or resorts to violence. |
|  | 23 |  | Depression | A state in which one is depressed and has no motivation. |
|  | 24 |  | Anxiety | A state in which one feels restless or fidgety. |
|  | 25 |  | Apathy | A state in which one has lost interest in one’s surroundings and is unable to take voluntary action. |
|  | 26 |  | Disinhibition | A state in which one is unable to suppress one’s emotions or desires and uses speech and actions that are not acceptable in society. |
|  | 27 |  | Aberrant motor behavior | A state in which one engages in unusual behavior (e.g., wanders around or checks something excessively). |
|  | 28 |  | Negativism | A state in which one refuses everything that is offered, including going to day service (Refusal to eat should be classified as anorexia). |
|  | 29 |  | Delirium | A state in which one’s psychological state deteriorates owing to being in an environment that is different from normal, such as being in a hospital or being ill. |
|  | 30 |  | Other psychiatric symptom | Other symptoms such as being excessively dependent on caregivers (dependence) or excessively persistent (obsession). |

|  |  | **Symptom domain** | **Symptom** | **Explanation** |
| --- | --- | --- | --- | --- |
| You may circle multiple items. | 31 | Eating behavior-related problems | Loss of appetite | A state in which one has little or no desire to eat. |
|  | 32 |  | Increase in appetite | A state in which one has a heightened desire to eat. |
|  | 33 |  | Weight loss | A state in which one loses weight. |
|  | 34 |  | Weight gain | A state in which one gains weight. |
|  | 35 |  | Food refusal | A state in which one says no to meals or refuses to eat even when being encouraged to eat. |
|  | 36 |  | Eating non-edible  things | A state in which one eats something that is not food. |
|  | 37 |  | Unbalanced diet | A state in which one is very picky about meals. |
|  | 38 | Sleep-related disorders | Rapid eye movement sleep behavior disorder | A state of sleep (dreaming) in which one talks in long sentences or talks as if in a conversation, yells, or does things like kicking and punching. |
|  | 39 |  | Daytime somnolence | A state in which one falls asleep during the daytime even after a good night’s sleep. |
|  | 40 |  | Day-night reversal | A state in which one stays awake during the night and sleeps during the day. |
|  | 41 |  | Night-time sleep disorder | A state in which one is unable to sleep well, wakes up many times at night, and wakes up early in the morning. |
|  | 42 |  | Sudden sleep | A state in which one suddenly falls asleep despite having been awake until that time. |
|  | 43 |  | Restless legs syndrome | A state in which one feels restless in the legs while sitting or lying down. |
|  | 44 |  | Periodic limb movement disorder | A state in which one or both arms or legs involuntarily move periodically and repeatedly at a set interval. |
|  | 45 | Autonomic dysfunction | Orthostatic hypotension | A state in which one feels like they are losing color or fainting, goes pale, or becomes dizzy when standing up from sitting or lying down. |
|  | 46 |  | Disturbance of  sweating | A state in which one sweats heavily only on the upper body or sweats only on the upper body despite feeling cold. |
|  | 47 |  | Constipation | A state in which one has poor bowel movements, which are sometimes accompanied by pain. |
|  | 48 |  | Night-time dysuria | Frequent urination (one gets up to go to the toilet three or more times during sleep), urinary incontinence, and a sensation of residual urine (one feels the urge to urinate even after urination). |
|  | 49 |  | Daytime dysuria | Frequent urination (one urinates eight or more times during the day), urinary incontinence, and a sensation of residual urine (one feels the urge to urinate even after urination). |
|  | 50 |  | Syncope | A state in which one experiences a temporary loss of consciousness but recovers after a few minutes (often observed after a meal or going to the toilet). |
|  | 51 |  | Dizziness | Dizziness is a term used to describe a range of sensations, such as feeling faint, woozy, weak or unsteady. |
|  | 52 | Sensory  disorders | Dysosmia | Dysosmia is a disorder described as any qualitative alteration or distortion of the perception of smell. |
|  | 53 | I do not know. | | |

Q45. Please select only one symptom that currently causes the patient the most distress.

|  |  | **Symptom domain** | **Symptom** | **Explanation** |
| --- | --- | --- | --- | --- |
| Please circle only one item. | 1 | Cognitive impairment | Memory impairment | A state in which one’s memory becomes extremely bad or one cannot recall things even with a hint. |
|  | 2 |  | Disorientation | A state in which one cannot tell what time of day it is or where they are. |
|  | 3 |  | Executive dysfunction | A state in which planning ahead to do something or to act in accordance with a procedure becomes difficult. |
|  | 4 |  | Attention dysfunction | A state in which one feels distracted or one’s attention or concentration is lost. |
|  | 5 |  | Fluctuating cognition | A state in which the cognitive function level varies from good to bad (stupor) and response levels fluctuate between lucid to reduced alertness. |
|  | 6 |  | Visuospatial dysfunction | A state in which one is unable to find something that is in front of them or is unable to accurately recognize something they see. |
|  | 7 |  | Other cognitive impairment | Other symptoms such as difficulty in speaking words (aphasia), difficulty in using items (apraxia), telling far-fetched stories (fabrication). |
|  | 8 | Parkinsonism | Bradykinesia/  Akinesia | A state in which it is difficult to move the body or move quickly. |
|  | 9 |  | Rigidity | A state in which relaxing the limbs or body is difficult, indicating that the muscles are always tense and stiff. |
|  | 10 |  | Action tremor | A state in which one’s hands shake when holding or writing something or one’s legs shake when sitting cross-legged. |
|  | 11 |  | Rest tremor | A state in which one’s hands and legs shake while resting relaxed, unlike when shaking occurs when trying to hold or write something. |
|  | 12 |  | Postural instability | A state in which keeping one’s balance is difficult and one feels like falling over. |
|  | 13 |  | Gait disturbance (short-stepped gait) | A state in which taking the first step is difficult or one shuffles or takes small steps. |
|  | 14 |  | Freezing of gait | A state in which taking the first step is difficult or one stands rooted to the spot and is unable to take the first step. |
|  | 15 |  | Abnormal posture | A state in which one’s neck is lowered or has a hunchback or forward-leaning posture. |
|  | 16 |  | Salivation | A state in which one is unable to swallow saliva and drools. |
|  | 17 |  | Fall | A state in which one often falls over on a flat road or stairs or falls off a chair from a sitting position. |
|  | 18 |  | Dysphagia | A state in which one has difficulty swallowing food and chokes or spits it out. |
|  | 19 | Psychiatric symptoms | Delusions | A state in which one falsely believes that their spouse is an imposter or is having an affair or that something was stolen. |
|  | 20 |  | Visual hallucinations | A state in which one sees something that does not really exist (person, animal, etc.). |
|  | 21 |  | Hallucinations other than visual hallucinations | A state in which one hears the voice of someone who is not present (auditory hallucination) or feels an illusory sensation and says something like “An insect is crawling under my skin” (cenesthesic hallucination). |
|  | 22 |  | Agitation/Aggression | A state in which one has a heightened sense of emotion, speaks violent words, or resorts to violence. |
|  | 23 |  | Depression | A state in which one is depressed and has no motivation. |
|  | 24 |  | Anxiety | A state in which one feels restless or fidgety. |
|  | 25 |  | Apathy | A state in which one has lost interest in one’s surroundings and is unable to take voluntary action. |
|  | 26 |  | Disinhibition | A state in which one is unable to suppress one’s emotions or desires and uses speech and actions that are not acceptable in society. |
|  | 27 |  | Aberrant motor behavior | A state in which one engages in unusual behavior (e.g., wanders around or checks something excessively). |
|  | 28 |  | Negativism | A state in which one refuses everything that is offered, including going to day service (Refusal to eat should be classified as anorexia). |
|  | 29 |  | Delirium | A state in which one’s psychological state deteriorates owing to being in an environment that is different from normal, such as being in a hospital or being ill. |
|  | 30 |  | Other psychiatric symptom | Other symptoms such as being excessively dependent on caregivers (dependence) or excessively persistent (obsession). |

|  |  | **Symptom domain** | **Symptom** | **Explanation** |
| --- | --- | --- | --- | --- |
| Please circle only one item. | 31 | Eating behavior-related problems | Loss of appetite | A state in which one has little or no desire to eat. |
|  | 32 |  | Increase in appetite | A state in which one has a heightened desire to eat. |
|  | 33 |  | Weight loss | A state in which one loses weight. |
|  | 34 |  | Weight gain | A state in which one gains weight. |
|  | 35 |  | Food refusal | A state in which one says no to meals or refuses to eat even when being encouraged to eat. |
|  | 36 |  | Eating non-edible  things | A state in which one eats something that is not food. |
|  | 37 |  | Unbalanced diet | A state in which one is very picky about meals. |
|  | 38 | Sleep-related disorders | Rapid eye movement sleep behavior disorder | A state of sleep (dreaming) in which one talks in long sentences or talks as if in a conversation, yells, or does things like kicking and punching. |
|  | 39 |  | Daytime somnolence | A state in which one falls asleep during the daytime even after a good night’s sleep. |
|  | 40 |  | Day-night reversal | A state in which one stays awake during the night and sleeps during the day. |
|  | 41 |  | Night-time sleep disorder | A state in which one is unable to sleep well, wakes up many times at night, and wakes up early in the morning. |
|  | 42 |  | Sudden sleep | A state in which one suddenly falls asleep despite having been awake until that time. |
|  | 43 |  | Restless legs syndrome | A state in which one feels restless in the legs while sitting or lying down. |
|  | 44 |  | Periodic limb movement disorder | A state in which one or both arms or legs involuntarily move periodically and repeatedly at a set interval. |
|  | 45 | Autonomic dysfunction | Orthostatic hypotension | A state in which one feels like they are losing color or fainting, goes pale, or becomes dizzy when standing up from sitting or lying down. |
|  | 46 |  | Disturbance of  sweating | A state in which one sweats heavily only on the upper body or sweats only on the upper body despite feeling cold. |
|  | 47 |  | Constipation | A state in which one has poor bowel movements, which are sometimes accompanied by pain. |
|  | 48 |  | Night-time dysuria | Frequent urination (one gets up to go to the toilet three or more times during sleep), urinary incontinence, and a sensation of residual urine (one feels the urge to urinate even after urination). |
|  | 49 |  | Daytime dysuria | Frequent urination (one urinates eight or more times during the day), urinary incontinence, and a sensation of residual urine (one feels the urge to urinate even after urination). |
|  | 50 |  | Syncope | A state in which one experiences a temporary loss of consciousness but recovers after a few minutes (often observed after a meal or going to the toilet). |
|  | 51 |  | Dizziness | Dizziness is a term used to describe a range of sensations, such as feeling faint, woozy, weak or unsteady. |
|  | 52 | Sensory  disorders | Dysosmia | Dysosmia is a disorder described as any qualitative alteration or distortion of the perception of smell. |
|  | 53 | I do not know. | | |

Q46. If you responded to Q45 and selected the symptom that causes the patient the most distress, what was the reason for selecting that symptom (selected in Q45)?

| Reason |  |
| --- | --- |

Q47. If you responded to Q45 and selected the symptom that causes the patient the most distress, have you informed the patient’s physician about that symptom (selected in Q45)?

| 1  2  3 | Yes.  Please circle only one item.  No.  I do not know. |
| --- | --- |

Q48. If you responded to Q45 and selected the symptom that causes the patient the most distress, has the patient’s physician inquired about that symptom (selected in Q45)?

| 1  2  3 | Yes.  Please circle only one item.  No.  I do not know. |
| --- | --- |

Q49. If you responded to Q45 and selected the symptom that causes the patient the most distress, has the patient complained about that symptom to you (selected in Q45)?

| 1  2  3 | Yes.  Please circle only one item.  No.  I do not know. |
| --- | --- |

Q50. If you responded to Q45 and selected the symptom that causes the patient the most distress, have you asked the patient about that symptom (selected in Q45)?

| 1  2  3 | Yes.  Please circle only one item.  No.  I do not know. |
| --- | --- |

| Please proceed to the following page ▶▶▶ |
| --- |

Q51.Please select all of the patient's other symptoms other than the symptom that currently causes the patient the most distress.

|  |  | **Symptom domain** | **Symptom** | **Explanation** |
| --- | --- | --- | --- | --- |
| You may circle multiple items. | 1 | Cognitive impairment | Memory impairment | A state in which one’s memory becomes extremely bad or one cannot recall things even with a hint. |
|  | 2 |  | Disorientation | A state in which one cannot tell what time of day it is or where they are. |
|  | 3 |  | Executive dysfunction | A state in which planning ahead to do something or to act in accordance with a procedure becomes difficult. |
|  | 4 |  | Attention dysfunction | A state in which one feels distracted or one’s attention or concentration is lost. |
|  | 5 |  | Fluctuating cognition | A state in which the cognitive function level varies from good to bad (stupor) and response levels fluctuate between lucid to reduced alertness. |
|  | 6 |  | Visuospatial dysfunction | A state in which one is unable to find something that is in front of them or is unable to accurately recognize something they see. |
|  | 7 |  | Other cognitive impairment | Other symptoms such as difficulty in speaking words (aphasia), difficulty in using items (apraxia), telling far-fetched stories (fabrication). |
|  | 8 | Parkinsonism | Bradykinesia/  Akinesia | A state in which it is difficult to move the body or move quickly. |
|  | 9 |  | Rigidity | A state in which relaxing the limbs or body is difficult, indicating that the muscles are always tense and stiff. |
|  | 10 |  | Action tremor | A state in which one’s hands shake when holding or writing something or one’s legs shake when sitting cross-legged. |
|  | 11 |  | Rest tremor | A state in which one’s hands and legs shake while resting relaxed, unlike when shaking occurs when trying to hold or write something. |
|  | 12 |  | Postural instability | A state in which keeping one’s balance is difficult and one feels like falling over. |
|  | 13 |  | Gait disturbance (short-stepped gait) | A state in which taking the first step is difficult or one shuffles or takes small steps. |
|  | 14 |  | Freezing of gait | A state in which taking the first step is difficult or one stands rooted to the spot and is unable to take the first step. |
|  | 15 |  | Abnormal posture | A state in which one’s neck is lowered or has a hunchback or forward-leaning posture. |
|  | 16 |  | Salivation | A state in which one is unable to swallow saliva and drools. |
|  | 17 |  | Fall | A state in which one often falls over on a flat road or stairs or falls off a chair from a sitting position. |
|  | 18 |  | Dysphagia | A state in which one has difficulty swallowing food and chokes or spits it out. |
|  | 19 | Psychiatric symptoms | Delusions | A state in which one falsely believes that their spouse is an imposter or is having an affair or that something was stolen. |
|  | 20 |  | Visual hallucinations | A state in which one sees something that does not really exist (person, animal, etc.). |
|  | 21 |  | Hallucinations other than visual hallucinations | A state in which one hears the voice of someone who is not present (auditory hallucination) or feels an illusory sensation and says something like “An insect is crawling under my skin” (cenesthesic hallucination). |
|  | 22 |  | Agitation/  Aggression | A state in which one has a heightened sense of emotion, speaks violent words, or resorts to violence. |
|  | 23 |  | Depression | A state in which one is depressed and has no motivation. |
|  | 24 |  | Anxiety | A state in which one feels restless or fidgety. |
|  | 25 |  | Apathy | A state in which one has lost interest in one’s surroundings and is unable to take voluntary action. |
|  | 26 |  | Disinhibition | A state in which one is unable to suppress one’s emotions or desires and uses speech and actions that are not acceptable in society. |
|  | 27 |  | Aberrant motor behavior | A state in which one engages in unusual behavior (e.g., wanders around or checks something excessively). |
|  | 28 |  | Negativism | A state in which one refuses everything that is offered, including going to day service (Refusal to eat should be classified as anorexia). |
|  | 29 |  | Delirium | A state in which one’s psychological state deteriorates owing to being in an environment that is different from normal, such as being in a hospital or being ill. |
|  | 30 |  | Other psychiatric symptom | Other symptoms such as being excessively dependent on caregivers (dependence) or excessively persistent (obsession). |

|  |  | **Symptom domain** | **Symptom** | **Explanation** |
| --- | --- | --- | --- | --- |
| You may circle multiple items. | 31 | Eating behavior-related problems | Loss of appetite | A state in which one has little or no desire to eat. |
|  | 32 |  | Increase in appetite | A state in which one has a heightened desire to eat. |
|  | 33 |  | Weight loss | A state in which one loses weight. |
|  | 34 |  | Weight gain | A state in which one gains weight. |
|  | 35 |  | Food refusal | A state in which one says no to meals or refuses to eat even when being encouraged to eat. |
|  | 36 |  | Eating non-edible  things | A state in which one eats something that is not food. |
|  | 37 |  | Unbalanced diet | A state in which one is very picky about meals. |
|  | 38 | Sleep-related disorders | Rapid eye movement sleep behavior disorder | A state of sleep (dreaming) in which one talks in long sentences or talks as if in a conversation, yells, or does things like kicking and punching. |
|  | 39 |  | Daytime somnolence | A state in which one falls asleep during the daytime even after a good night’s sleep. |
|  | 40 |  | Day-night reversal | A state in which one stays awake during the night and sleeps during the day. |
|  | 41 |  | Night-time sleep disorder | A state in which one is unable to sleep well, wakes up many times at night, and wakes up early in the morning. |
|  | 42 |  | Sudden sleep | A state in which one suddenly falls asleep despite having been awake until that time. |
|  | 43 |  | Restless legs syndrome | A state in which one feels restless in the legs while sitting or lying down. |
|  | 44 |  | Periodic limb movement disorder | A state in which one or both arms or legs involuntarily move periodically and repeatedly at a set interval. |
|  | 45 | Autonomic dysfunction | Orthostatic hypotension | A state in which one feels like they are losing color or fainting, goes pale, or becomes dizzy when standing up from sitting or lying down. |
|  | 46 |  | Disturbance of  sweating | A state in which one sweats heavily only on the upper body or sweats only on the upper body despite feeling cold. |
|  | 47 |  | Constipation | A state in which one has poor bowel movements, which are sometimes accompanied by pain. |
|  | 48 |  | Night-time dysuria | Frequent urination (one gets up to go to the toilet three or more times during sleep), urinary incontinence, and a sensation of residual urine (one feels the urge to urinate even after urination). |
|  | 49 |  | Daytime dysuria | Frequent urination (one urinates eight or more times during the day), urinary incontinence, and a sensation of residual urine (one feels the urge to urinate even after urination). |
|  | 50 |  | Syncope | A state in which one experiences a temporary loss of consciousness but recovers after a few minutes (often observed after a meal or going to the toilet). |
|  | 51 |  | Dizziness | Dizziness is a term used to describe a range of sensations, such as feeling faint, woozy, weak or unsteady. |
|  | 52 | Sensory  disorders | Dysosmia | Dysosmia is a disorder described as any qualitative alteration or distortion of the perception of smell. |
|  | 53 | No other troubling symptoms. | | |
|  | 54 | I do not know. | | |

Q52. As the patient continues with treatment, which patient’s symptom do you think the patient would most likely prioritize for receiving treatment? Please select only one.

|  |  | **Symptom domain** | **Symptom** | **Explanation** |
| --- | --- | --- | --- | --- |
| Please circle only one item. | 1 | Cognitive impairment | Memory impairment | A state in which one’s memory becomes extremely bad or one cannot recall things even with a hint. |
|  | 2 |  | Disorientation | A state in which one cannot tell what time of day it is or where they are. |
|  | 3 |  | Executive dysfunction | A state in which planning ahead to do something or to act in accordance with a procedure becomes difficult. |
|  | 4 |  | Attention dysfunction | A state in which one feels distracted or one’s attention or concentration is lost. |
|  | 5 |  | Fluctuating cognition | A state in which the cognitive function level varies from good to bad (stupor) and response levels fluctuate between lucid to reduced alertness. |
|  | 6 |  | Visuospatial dysfunction | A state in which one is unable to find something that is in front of them or is unable to accurately recognize something they see. |
|  | 7 |  | Other cognitive impairment | Other symptoms such as difficulty in speaking words (aphasia), difficulty in using items (apraxia), telling far-fetched stories (fabrication). |
|  | 8 | Parkinsonism | Bradykinesia/  Akinesia | A state in which it is difficult to move the body or move quickly. |
|  | 9 |  | Rigidity | A state in which relaxing the limbs or body is difficult, indicating that the muscles are always tense and stiff. |
|  | 10 |  | Action tremor | A state in which one’s hands shake when holding or writing something or one’s legs shake when sitting cross-legged. |
|  | 11 |  | Rest tremor | A state in which one’s hands and legs shake while resting relaxed, unlike when shaking occurs when trying to hold or write something. |
|  | 12 |  | Postural instability | A state in which keeping one’s balance is difficult and one feels like falling over. |
|  | 13 |  | Gait disturbance (short-stepped gait) | A state in which taking the first step is difficult or one shuffles or takes small steps. |
|  | 14 |  | Freezing of gait | A state in which taking the first step is difficult or one stands rooted to the spot and is unable to take the first step. |
|  | 15 |  | Abnormal posture | A state in which one’s neck is lowered or has a hunchback or forward-leaning posture. |
|  | 16 |  | Salivation | A state in which one is unable to swallow saliva and drools. |
|  | 17 |  | Fall | A state in which one often falls over on a flat road or stairs or falls off a chair from a sitting position. |
|  | 18 |  | Dysphagia | A state in which one has difficulty swallowing food and chokes or spits it out. |
|  | 19 | Psychiatric symptoms | Delusions | A state in which one falsely believes that their spouse is an imposter or is having an affair or that something was stolen. |
|  | 20 |  | Visual hallucinations | A state in which one sees something that does not really exist (person, animal, etc.). |
|  | 21 |  | Hallucinations other than visual hallucinations | A state in which one hears the voice of someone who is not present (auditory hallucination) or feels an illusory sensation and says something like “An insect is crawling under my skin” (cenesthesic hallucination). |
|  | 22 |  | Agitation/  Aggression | A state in which one has a heightened sense of emotion, speaks violent words, or resorts to violence. |
|  | 23 |  | Depression | A state in which one is depressed and has no motivation. |
|  | 24 |  | Anxiety | A state in which one feels restless or fidgety. |
|  | 25 |  | Apathy | A state in which one has lost interest in one’s surroundings and is unable to take voluntary action. |
|  | 26 |  | Disinhibition | A state in which one is unable to suppress one’s emotions or desires and uses speech and actions that are not acceptable in society. |
|  | 27 |  | Aberrant motor behavior | A state in which one engages in unusual behavior (e.g., wanders around or checks something excessively). |
|  | 28 |  | Negativism | A state in which one refuses everything that is offered, including going to day service (Refusal to eat should be classified as anorexia). |
|  | 29 |  | Delirium | A state in which one’s psychological state deteriorates owing to being in an environment that is different from normal, such as being in a hospital or being ill. |
|  | 30 |  | Other psychiatric symptom | Other symptoms such as being excessively dependent on caregivers (dependence) or excessively persistent (obsession). |

|  |  | **Symptom domain** | **Symptom** | **Explanation** |
| --- | --- | --- | --- | --- |
| Please circle only one item. | 31 | Eating behavior-related problems | Loss of appetite | A state in which one has little or no desire to eat. |
|  | 32 |  | Increase in appetite | A state in which one has a heightened desire to eat. |
|  | 33 |  | Weight loss | A state in which one loses weight. |
|  | 34 |  | Weight gain | A state in which one gains weight. |
|  | 35 |  | Food refusal | A state in which one says no to meals or refuses to eat even when being encouraged to eat. |
|  | 36 |  | Eating non-edible  things | A state in which one eats something that is not food. |
|  | 37 |  | Unbalanced diet | A state in which one is very picky about meals. |
|  | 38 | Sleep-related disorders | Rapid eye movement sleep behavior disorder | A state of sleep (dreaming) in which one talks in long sentences or talks as if in a conversation, yells, or does things like kicking and punching. |
|  | 39 |  | Daytime somnolence | A state in which one falls asleep during the daytime even after a good night’s sleep. |
|  | 40 |  | Day-night reversal | A state in which one stays awake during the night and sleeps during the day. |
|  | 41 |  | Night-time sleep disorder | A state in which one is unable to sleep well, wakes up many times at night, and wakes up early in the morning. |
|  | 42 |  | Sudden sleep | A state in which one suddenly falls asleep despite having been awake until that time. |
|  | 43 |  | Restless legs syndrome | A state in which one feels restless in the legs while sitting or lying down. |
|  | 44 |  | Periodic limb movement disorder | A state in which one or both arms or legs involuntarily move periodically and repeatedly at a set interval. |
|  | 45 | Autonomic dysfunction | Orthostatic hypotension | A state in which one feels like they are losing color or fainting, goes pale, or becomes dizzy when standing up from sitting or lying down. |
|  | 46 |  | Disturbance of  sweating | A state in which one sweats heavily only on the upper body or sweats only on the upper body despite feeling cold. |
|  | 47 |  | Constipation | A state in which one has poor bowel movements, which are sometimes accompanied by pain. |
|  | 48 |  | Night-time dysuria | Frequent urination (one gets up to go to the toilet three or more times during sleep), urinary incontinence, and a sensation of residual urine (one feels the urge to urinate even after urination). |
|  | 49 |  | Daytime dysuria | Frequent urination (one urinates eight or more times during the day), urinary incontinence, and a sensation of residual urine (one feels the urge to urinate even after urination). |
|  | 50 |  | Syncope | A state in which one experiences a temporary loss of consciousness but recovers after a few minutes (often observed after a meal or going to the toilet). |
|  | 51 |  | Dizziness | Dizziness is a term used to describe a range of sensations, such as feeling faint, woozy, weak or unsteady. |
|  | 52 | Sensory  disorders | Dysosmia | Dysosmia is a disorder described as any qualitative alteration or distortion of the perception of smell. |
|  | 53 | I do not know. | | |

Q53. Please confirm your response to Q43 on page 23–24.

Did you select any items in 19–44?

| 1  2 | Yes  No | ▶  ▶ | Go to Q54  Please circle only one item.  Go to Q63 |
| --- | --- | --- | --- |

Q54. Did the patient’s physician explain about psychiatric symptoms, eating behavior-related problems, and sleep-related disorders to you?

* Please refer to the table on the following page regarding Psychiatric symptoms, Eating behavior-related problems, and Sleep-related disorders.

| 1  2  3  4 | The patient’s physician explained.  Please circle only  one item.  The patient’s physician explained partially.  The patient’s physician did not explain.  I do not know. |
| --- | --- |

Q55. If you selected “The patient’s physician explained” or “The patient’s physician explained partially” in Q54, did you inform the patient about the physician’s explanation regarding psychiatric symptoms, eating behavior-related problems, and sleep-related disorders?

* Please refer to the table on the following page regarding Psychiatric symptoms, Eating behavior-related problems, and Sleep-related disorders.

| 1  2  3  4  5 | Yes.  Partially.  Please circle only one item.  No.  No, because I always hear it with the patient.  I do not know. |
| --- | --- |

**※** Psychiatric symptoms, Eating behavior-related problems, and Sleep-related disorders are as follows:

|  | **Symptom** | **Explanation** |
| --- | --- | --- |
| Psychiatric symptoms | Delusions | A state in which one falsely believes that their spouse is an imposter or is having an affair or that something was stolen. |
|  | Visual hallucinations | A state in which one sees something that does not really exist (person, animal, etc.). |
|  | Hallucinations other than visual hallucinations | A state in which one hears the voice of someone who is not present (auditory hallucination) or feels an illusory sensation and says something like “An insect is crawling under my skin” (cenesthesic hallucination). |
|  | Agitation/Aggression | A state in which one has a heightened sense of emotion, speaks violent words, or resorts to violence. |
|  | Depression | A state in which one is depressed and has no motivation. |
|  | Anxiety | A state in which one feels restless or fidgety. |
|  | Apathy | A state in which one has lost interest in one’s surroundings and is unable to take voluntary action. |
|  | Disinhibition | A state in which one is unable to suppress one’s emotions or desires and uses speech and actions that are not acceptable in society. |
|  | Aberrant motor behavior | A state in which one engages in unusual behavior (e.g., wanders around or checks something excessively). |
|  | Negativism | A state in which one refuses everything that is offered, including going to day service (Refusal to eat should be classified as anorexia). |
|  | Delirium | A state in which one’s psychological state deteriorates owing to being in an environment that is different from normal, such as being in a hospital or being ill. |
|  | Other psychiatric symptom | Other symptoms such as being excessively dependent on caregivers (dependence) or excessively persistent (obsession). |
| Eating behavior-related problems | Loss of appetite | A state in which one has little or no desire to eat. |
|  | Increase in appetite | A state in which one has a heightened desire to eat. |
|  | Weight loss | A state in which one loses weight. |
|  | Weight gain | A state in which one gains weight. |
|  | Food refusal | A state in which one says no to meals or refuses to eat even when being encouraged to eat. |
|  | Eating non-edible things | A state in which one eats something that is not food. |
|  | Unbalanced diet | A state in which one is very picky about meals. |
| Sleep-related disorders | Rapid eye movement sleep behavior disorder | A state of sleep (dreaming) in which one talks in long sentences or talks as if in a conversation, yells, or does things like kicking and punching. |
|  | Daytime somnolence | A state in which one falls asleep during the daytime even after a good night’s sleep. |
|  | Day–night reversal | A state in which one stays awake during the night and sleeps during the day. |
|  | Night-time sleep disorder | A state in which one is unable to sleep well, wakes up many times at night, and wakes up early in the morning. |
|  | Sudden sleep | A state in which one suddenly falls asleep despite having been awake until that time. |
|  | Restless legs syndrome | A state in which one feels restless in the legs while sitting or lying down. |
|  | Periodic limb movement disorder | A state in which one or both arms or legs involuntarily move periodically and repeatedly at a set interval. |

| Please respond to the following questions based on what you believe (feel).  (Q56–Q59) |
| --- |

| Please proceed to the following page ▶▶▶ |
| --- |

Q56. If you selected any items in 19–44 in Q43, which is the patient’s symptom that causes you the most distress among those classified under psychiatric symptoms, eating behavior-related problems, and sleep related-disorders? Please select only one.

|  |  |  | **Symptom** | **Explanation** |
| --- | --- | --- | --- | --- |
| Please circle only one item. | 1 | Psychiatric symptoms | Delusions | A state in which one falsely believes that their spouse is an imposter or is having an affair or that something was stolen. |
|  | 2 |  | Visual hallucinations | A state in which one sees something that does not really exist (person, animal, etc.). |
|  | 3 |  | Hallucinations other than visual hallucinations | A state in which one hears the voice of someone who is not present (auditory hallucination) or feels an illusory sensation and says something like “An insect is crawling under my skin” (cenesthesic hallucination). |
|  | 4 |  | Agitation/Aggression | A state in which one has a heightened sense of emotion, speaks violent words, or resorts to violence. |
|  | 5 |  | Depression | A state in which one is depressed and has no motivation. |
|  | 6 |  | Anxiety | A state in which one feels restless or fidgety. |
|  | 7 |  | Apathy | A state in which one has lost interest in one’s surroundings and is unable to take voluntary action. |
|  | 8 |  | Disinhibition | A state in which one is unable to suppress one’s emotions or desires and uses speech and actions that are not acceptable in society. |
|  | 9 |  | Aberrant motor behavior | A state in which one engages in unusual behavior (e.g., wanders around or checks something excessively). |
|  | 10 |  | Negativism | A state in which one refuses everything that is offered, including going to day service (Refusal to eat should be classified as anorexia). |
|  | 11 |  | Delirium | A state in which one’s psychological state deteriorates owing to being in an environment that is different from normal, such as being in a hospital or being ill. |
|  | 12 |  | Other psychiatric symptom | Other symptoms such as being excessively dependent on caregivers (dependence) or excessively persistent (obsession). |
|  | 13 | Eating behavior-related problems | Loss of appetite | A state in which one has little or no desire to eat. |
|  | 14 |  | Increase in appetite | A state in which one has a heightened desire to eat. |
|  | 15 |  | Weight loss | A state in which one loses weight. |
|  | 16 |  | Weight gain | A state in which one gains weight. |
|  | 17 |  | Food refusal | A state in which one says no to meals or refuses to eat even when being encouraged to eat. |
|  | 18 |  | Eating non-edible things | A state in which one eats something that is not food. |
|  | 19 |  | Unbalanced diet | A state in which one is very picky about meals. |
|  | 20 | Sleep-related disorders | Rapid eye movement sleep behavior disorder | A state of sleep (dreaming) in which one talks in long sentences or talks as if in a conversation, yells, or does things like kicking and punching. |
|  | 21 |  | Daytime somnolence | A state in which one falls asleep during the daytime even after a good night’s sleep. |
|  | 22 |  | Day–night reversal | A state in which one stays awake during the night and sleeps during the day. |
|  | 23 |  | Night-time sleep disorder | A state in which one is unable to sleep well, wakes up many times at night, and wakes up early in the morning. |
|  | 24 |  | Sudden sleep | A state in which one suddenly falls asleep despite having been awake until that time. |
|  | 25 |  | Restless legs syndrome | A state in which one feels restless in the legs while sitting or lying down. |
|  | 26 |  | Periodic limb movement disorder | A state in which one or both arms or legs involuntarily move periodically and repeatedly at a set interval. |
|  | 27 | I do not know. | | |

Q57. If you responded to Q56 and selected the patient’s symptom that causes you the most distress, would you prefer the patient to receive treatment for that symptom (selected in Q56)?

| 1  2  3 | Yes.  Please circle only one item.  No.  I do not know. |
| --- | --- |

Q58. If you responded to Q56 and selected the patient’s symptom that causes you the most distress, do you think the patient is being treated (receiving medication or advice from the patient’s physician) for that symptom (selected in Q56)?

| 1  2  3 | Yes.  Please circle only one item.  No.  I do not know. |
| --- | --- |

Q59. If you selected any items in 19–44 in Q43, as the patient continues with treatment, which patient’s symptoms among those classified under psychiatric symptoms, eating behavior-related problems, and sleep related-disorders would you prioritize for receiving treatment? Please select all applicable items.

|  |  |  | **Symptom** | **Explanation** |
| --- | --- | --- | --- | --- |
| You may circle multiple items. | 1 | Psychiatric symptoms | Delusions | A state in which one falsely believes that their spouse is an imposter or is having an affair or that something was stolen. |
|  | 2 |  | Visual hallucinations | A state in which one sees something that does not really exist (person, animal, etc.). |
|  | 3 |  | Hallucinations other than visual hallucinations | A state in which one hears the voice of someone who is not present (auditory hallucination) or feels an illusory sensation and says something like “An insect is crawling under my skin” (cenesthesic hallucination). |
|  | 4 |  | Agitation/Aggression | A state in which one has a heightened sense of emotion, speaks violent words, or resorts to violence. |
|  | 5 |  | Depression | A state in which one is depressed and has no motivation. |
|  | 6 |  | Anxiety | A state in which one feels restless or fidgety. |
|  | 7 |  | Apathy | A state in which one has lost interest in one’s surroundings and is unable to take voluntary action. |
|  | 8 |  | Disinhibition | A state in which one is unable to suppress one’s emotions or desires and uses speech and actions that are not acceptable in society. |
|  | 9 |  | Aberrant motor behavior | A state in which one engages in unusual behavior (e.g., wanders around or checks something excessively). |
|  | 10 |  | Negativism | A state in which one refuses everything that is offered, including going to day service (Refusal to eat should be classified as anorexia). |
|  | 11 |  | Delirium | A state in which one’s psychological state deteriorates owing to being in an environment that is different from normal, such as being in a hospital or being ill. |
|  | 12 |  | Other psychiatric symptom | Other symptoms such as being excessively dependent on caregivers (dependence) or excessively persistent (obsession). |
|  | 13 | Eating behavior-related problems | Loss of appetite | A state in which one has little or no desire to eat. |
|  | 14 |  | Increase in appetite | A state in which one has a heightened desire to eat. |
|  | 15 |  | Weight loss | A state in which one loses weight. |
|  | 16 |  | Weight gain | A state in which one gains weight. |
|  | 17 |  | Food refusal | A state in which one says no to meals or refuses to eat even when being encouraged to eat. |
|  | 18 |  | Eating non-edible things | A state in which one eats something that is not food. |
|  | 19 |  | Unbalanced diet | A state in which one is very picky about meals. |
|  | 20 | Sleep-related disorders | Rapid eye movement sleep behavior disorder | A state of sleep (dreaming) in which one talks in long sentences or talks as if in a conversation, yells, or does things like kicking and punching. |
|  | 21 |  | Daytime somnolence | A state in which one falls asleep during the daytime even after a good night’s sleep. |
|  | 22 |  | Day–night reversal | A state in which one stays awake during the night and sleeps during the day. |
|  | 23 |  | Night-time sleep disorder | A state in which one is unable to sleep well, wakes up many times at night, and wakes up early in the morning. |
|  | 24 |  | Sudden sleep | A state in which one suddenly falls asleep despite having been awake until that time. |
|  | 25 |  | Restless legs syndrome | A state in which one feels restless in the legs while sitting or lying down. |
|  | 26 |  | Periodic limb movement disorder | A state in which one or both arms or legs involuntarily move periodically and repeatedly at a set interval. |
|  | 27 | I do not know. | | |

| Please consider the patient and respond to the following questions without discussing them with the patient.  (Q60–Q62) |
| --- |

| Please proceed to the following page ▶▶▶ |
| --- |

Q60. If you selected any items in 19–44 in Q43, which is the patient’s symptom that currently causes the patient the most distress among those classified under psychiatric symptoms, eating behavior-related problems, and sleep-related disorders? Please select only one.

|  |  |  | **Symptom** | **Explanation** |
| --- | --- | --- | --- | --- |
| Please circle only one item. | 1 | Psychiatric symptoms | Delusions | A state in which one falsely believes that their spouse is an imposter or is having an affair or that something was stolen. |
|  | 2 |  | Visual hallucinations | A state in which one sees something that does not really exist (person, animal, etc.). |
|  | 3 |  | Hallucinations other than visual hallucinations | A state in which one hears the voice of someone who is not present (auditory hallucination) or feels an illusory sensation and says something like “An insect is crawling under my skin” (cenesthesic hallucination). |
|  | 4 |  | Agitation/Aggression | A state in which one has a heightened sense of emotion, speaks violent words, or resorts to violence. |
|  | 5 |  | Depression | A state in which one is depressed and has no motivation. |
|  | 6 |  | Anxiety | A state in which one feels restless or fidgety. |
|  | 7 |  | Apathy | A state in which one has lost interest in one’s surroundings and is unable to take voluntary action. |
|  | 8 |  | Disinhibition | A state in which one is unable to suppress one’s emotions or desires and uses speech and actions that are not acceptable in society. |
|  | 9 |  | Aberrant motor behavior | A state in which one engages in unusual behavior (e.g., wanders around or checks something excessively). |
|  | 10 |  | Negativism | A state in which one refuses everything that is offered, including going to day service (Refusal to eat should be classified as anorexia). |
|  | 11 |  | Delirium | A state in which one’s psychological state deteriorates owing to being in an environment that is different from normal, such as being in a hospital or being ill. |
|  | 12 |  | Other psychiatric symptom | Other symptoms such as being excessively dependent on caregivers (dependence) or excessively persistent (obsession). |
|  | 13 | Eating behavior-related problems | Loss of appetite | A state in which one has little or no desire to eat. |
|  | 14 |  | Increase in appetite | A state in which one has a heightened desire to eat. |
|  | 15 |  | Weight loss | A state in which one loses weight. |
|  | 16 |  | Weight gain | A state in which one gains weight. |
|  | 17 |  | Food refusal | A state in which one says no to meals or refuses to eat even when being encouraged to eat. |
|  | 18 |  | Eating non-edible things | A state in which one eats something that is not food. |
|  | 19 |  | Unbalanced diet | A state in which one is very picky about meals. |
|  | 20 | Sleep-related disorders | Rapid eye movement sleep behavior disorder | A state of sleep (dreaming) in which one talks in long sentences or talks as if in a conversation, yells, or does things like kicking and punching. |
|  | 21 |  | Daytime somnolence | A state in which one falls asleep during the daytime even after a good night’s sleep. |
|  | 22 |  | Day–night reversal | A state in which one stays awake during the night and sleeps during the day. |
|  | 23 |  | Night-time sleep disorder | A state in which one is unable to sleep well, wakes up many times at night, and wakes up early in the morning. |
|  | 24 |  | Sudden sleep | A state in which one suddenly falls asleep despite having been awake until that time. |
|  | 25 |  | Restless legs syndrome | A state in which one feels restless in the legs while sitting or lying down. |
|  | 26 |  | Periodic limb movement disorder | A state in which one or both arms or legs involuntarily move periodically and repeatedly at a set interval. |
|  | 27 | I do not know. | | |

Q61. If you responded to Q60 and selected the patient’s symptom that causes the patient the most distress, would you prefer the patient to receive treatment for that symptom (selected in Q60)?

| 1  2  3 | Yes.  Please circle only one item.  No.  I do not know. |
| --- | --- |

Q62. If you responded to Q60 and selected the patient’s symptom that causes the patient the most distress, do you think the patient is being treated (receiving medication or advice from the patient’s physician) for that symptom (selected in Q60)?

| 1  2  3 | Yes.  Please circle only one item.  No.  I do not know. |
| --- | --- |

Q63. Please confirm your response to Q43 on page 23–24.

Did you select any items in 8–18?

| 1  2 | Yes  No | ▶  ▶ | Go to Q64  Please circle only one item.  Go to Q74 |
| --- | --- | --- | --- |

Q64. Did the patient’s physician explain about parkinsonism to you?

* Please refer to the table below regarding parkinsonism.

| 1  2  3  4 | The patient’s physician explained.  Please circle only one item.  The patient’s physician explained partially.  The patient’s physician did not explain.  I do not know. |
| --- | --- |

**※** Parkinsonism includes the following symptoms

|  | **Symptom** | **Explanation** |
| --- | --- | --- |
| Parkinsonism | Bradykinesia/  Akinesia | A state in which it is difficult to move the body or move quickly. |
|  | Rigidity | A state in which relaxing the limbs or body is difficult, indicating that the muscles are always tense and stiff. |
|  | Action tremor | A state in which one’s hands shake when holding or writing something or one’s legs shake when sitting cross-legged. |
|  | Rest tremor | A state in which one’s hands and legs shake while resting relaxed, unlike when shaking occurs when trying to hold or write something. |
|  | Postural instability | A state in which keeping one’s balance is difficult and one feels like falling over. |
|  | Gait disturbance (short-stepped gait) | A state in which taking the first step is difficult or one shuffles or takes small steps. |
|  | Freezing of gait | A state in which taking the first step is difficult or one stands rooted to the spot and is unable to take the first step. |
|  | Abnormal posture | A state in which one’s neck is lowered or has a hunchback or forward-leaning posture. |
|  | Salivation | A state in which one is unable to swallow saliva and drools. |
|  | Fall | A state in which one often falls over on a flat road or stairs or falls off a chair from a sitting position. |
|  | Dysphagia | A state in which one has difficulty swallowing food and chokes or spits it out. |

Q65. If you selected “The patient’s physician explained” or “The patient’s physician explained partially” in Q64, did you inform the patient about the physician’s explanation regarding parkinsonism?

| 1  2  3  4  5 | Yes.  Partially.  Please circle only one item.  No.  No, because I always hear it with the patient.  I do not know. |
| --- | --- |

Q66. Levodopa formulations include Madopar and Menesit. Has a levodopa formulation been prescribed to the patient? What was its effect on parkinsonism when the patient started taking it? Please select one applicable item.

| 1  2  3  4  5  6  7 | It was extremely effective.  It was moderately effective.  No change was observed.  It was not very effective.  Please circle only one item.  It was not effective at all.  The patient has never taken it.  I do not know. I cannot recall. |
| --- | --- |

| Please respond to the following questions based on what you believe (feel).  (Q67–Q70) |
| --- |

Q67. If you selected any items in 8–18 in Q43, which is the patient’s symptom of parkinsonism that causes you the most distress? Please select only one.

|  |  |  | **Symptom** | **Explanation** |
| --- | --- | --- | --- | --- |
| Please circle only one item. | 1 | Parkinsonism | Bradykinesia/  Akinesia | A state in which it is difficult to move the body or move quickly. |
|  | 2 |  | Rigidity | A state in which relaxing the limbs or body is difficult, indicating that the muscles are always tense and stiff. |
|  | 3 |  | Action tremor | A state in which one’s hands shake when holding or writing something or one’s legs shake when sitting cross-legged. |
|  | 4 |  | Rest tremor | A state in which one’s hands and legs shake while resting relaxed, unlike when shaking occurs when trying to hold or write something. |
|  | 5 |  | Postural instability | A state in which keeping one’s balance is difficult and one feels like falling over. |
|  | 6 |  | Gait disturbance (short-stepped gait) | A state in which taking the first step is difficult or one shuffles or takes small steps. |
|  | 7 |  | Freezing of gait | A state in which taking the first step is difficult or one stands rooted to the spot and is unable to take the first step. |
|  | 8 |  | Abnormal posture | A state in which one’s neck is lowered or has a hunchback or forward-leaning posture. |
|  | 9 |  | Salivation | A state in which one is unable to swallow saliva and drools. |
|  | 10 |  | Fall | A state in which one often falls over on a flat road or stairs or falls off a chair from a sitting position. |
|  | 11 |  | Dysphagia | A state in which one has difficulty swallowing food and chokes or spits it out. |
|  | 12 | I do not know. | | |

Q68. If you responded to Q67 and selected the patient’s symptom of parkinsonism that causes you the most distress, would you prefer the patient to receive treatment for that symptom (selected in Q67)?

| 1  2  3 | Yes.  Please circle only one item.  No.  I do not know. |
| --- | --- |

Q69. If you responded to Q67 and selected the patient’s symptom of parkinsonism that causes you the most distress, do you think the patient is being treated (receiving medication or advice from the patient’s physician) for that symptom (selected in Q67)?

| 1  2  3 | Yes.  Please circle only one item.  No.  I do not know. |
| --- | --- |

Q70. If you selected any items in 8–18 in Q43, as the patient continues with treatment, which patient’s symptoms of parkinsonism would you prioritize for receiving treatment? Please select all applicable items.

|  |  |  | **Symptom** | **Explanation** |
| --- | --- | --- | --- | --- |
| You may circle multiple items. | 1 | Parkinsonism | Bradykinesia/  Akinesia | A state in which it is difficult to move the body or move quickly. |
|  | 2 |  | Rigidity | A state in which relaxing the limbs or body is difficult, indicating that the muscles are always tense and stiff. |
|  | 3 |  | Action tremor | A state in which one’s hands shake when holding or writing something or one’s legs shake when sitting cross-legged. |
|  | 4 |  | Rest tremor | A state in which one’s hands and legs shake while resting relaxed, unlike when shaking occurs when trying to hold or write something. |
|  | 5 |  | Postural instability | A state in which keeping one’s balance is difficult and one feels like falling over. |
|  | 6 |  | Gait disturbance (short-stepped gait) | A state in which taking the first step is difficult or one shuffles or takes small steps. |
|  | 7 |  | Freezing of gait | A state in which taking the first step is difficult or one stands rooted to the spot and is unable to take the first step. |
|  | 8 |  | Abnormal posture | A state in which one’s neck is lowered or has a hunchback or forward-leaning posture. |
|  | 9 |  | Salivation | A state in which one is unable to swallow saliva and drools. |
|  | 10 |  | Fall | A state in which one often falls over on a flat road or stairs or falls off a chair from a sitting position. |
|  | 11 |  | Dysphagia | A state in which one has difficulty swallowing food and chokes or spits it out. |
|  | 12 | I do not know. | | |

| Please consider the patient and respond to the following questions without discussing them with the patient.  (Q71–Q73) |
| --- |

Q71. If you selected any items in 8–18 in Q43, which is the patient’s symptom of parkinsonism that causes the patient the most distress? Please select only one.

|  |  |  | **Symptom** | **Explanation** |
| --- | --- | --- | --- | --- |
| Please circle only one item. | 1 | Parkinsonism | Bradykinesia/  Akinesia | A state in which it is difficult to move the body or move quickly. |
|  | 2 |  | Rigidity | A state in which relaxing the limbs or body is difficult, indicating that the muscles are always tense and stiff. |
|  | 3 |  | Action tremor | A state in which one’s hands shake when holding or writing something or one’s legs shake when sitting cross-legged. |
|  | 4 |  | Rest tremor | A state in which one’s hands and legs shake while resting relaxed, unlike when shaking occurs when trying to hold or write something. |
|  | 5 |  | Postural instability | A state in which keeping one’s balance is difficult and one feels like falling over. |
|  | 6 |  | Gait disturbance (short-stepped gait) | A state in which taking the first step is difficult or one shuffles or takes small steps. |
|  | 7 |  | Freezing of gait | A state in which taking the first step is difficult or one stands rooted to the spot and is unable to take the first step. |
|  | 8 |  | Abnormal posture | A state in which one’s neck is lowered or has a hunchback or forward-leaning posture. |
|  | 9 |  | Salivation | A state in which one is unable to swallow saliva and drools. |
|  | 10 |  | Fall | A state in which one often falls over on a flat road or stairs or falls off a chair from a sitting position. |
|  | 11 |  | Dysphagia | A state in which one has difficulty swallowing food and chokes or spits it out. |
|  | 12 | I do not know. | | |

Q72. If you responded to Q71 and selected the patient’s symptom that causes the patient the most distress, would you prefer the patient to receive treatment for that symptom (selected in Q71)?

| 1  2  3 | Yes.  Please circle only one item.  No.  I do not know. |
| --- | --- |

Q73. If you responded to Q71 and selected the patient’s symptom that causes the patient the most distress, do you think the patient is being treated (receiving medication or advice from the patient’s physician) for that symptom (selected in Q71)?

| 1  2  3 | Yes.  Please circle only one item.  No.  I do not know. |
| --- | --- |

Q74. Please select the degree of satisfaction in terms of the treatment effect of the patient’s medication currently prescribed for cognitive impairment.

| 1  2  3  4  5  6  7 | I am extremely satisfied.  I am moderately satisfied.  It is difficult to select either.  Please circle only one item.  I am slightly dissatisfied.  I am extremely dissatisfied.  The patient does not take it.  I do not know. |
| --- | --- |

Q75. Please select the degree of satisfaction in terms of the treatment effect of the patient’s medication currently prescribed for parkinsonism.

| 1  2  3  4  5  6  7 | I am extremely satisfied.  I am moderately satisfied.  It is difficult to select either.  Please circle only one item.  I am slightly dissatisfied.  I am extremely dissatisfied.  The patient does not take it.  I do not know. |
| --- | --- |

Q76. Please select the degree of satisfaction in terms of the treatment effect of the patient’s medication currently prescribed for psychiatric symptoms.

| 1  2  3  4  5  6  7 | I am extremely satisfied.  I am moderately satisfied.  It is difficult to select either.  Please circle only one item.  I am slightly dissatisfied.  I am extremely dissatisfied.  The patient does not take it.  I do not know. |
| --- | --- |

Q77. Please select the degree of satisfaction in terms of the treatment effect of the patient’s medication currently prescribed for sleep-related disorders.

| 1  2  3  4  5  6  7 | I am extremely satisfied.  I am moderately satisfied.  It is difficult to select either.  Please circle only one item.  I am slightly dissatisfied.  I am extremely dissatisfied.  The patient does not take it.  I do not know. |
| --- | --- |

Q78. Please select the degree of satisfaction in terms of the treatment effect of the patient’s medication currently prescribed for autonomic dysfunction.

| 1  2  3  4  5  6  7 | I am extremely satisfied.  I am moderately satisfied.  It is difficult to select either.  Please circle only one item.  I am slightly dissatisfied.  I am extremely dissatisfied.  The patient does not take it.  I do not know. |
| --- | --- |

| **The questionnaire is complete.**  **Thank you for your cooperation.** |
| --- |
