# Supplementary material for: Treatment needs of dementia with Lewy bodies according to patients, caregivers, and physicians: a cross-sectional, observational, questionnaire-based study in Japan
Source: Alzheimers Res Ther. 2022 Dec 15;14:188. doi: 10.1186/s13195-022-01130-4 (PMC9751509; doi:10.1186/s13195-022-01130-4)
Supplement: Supplementary file 4 — Additional file 4: Supplementary Methods 3. Questionnaire for physician: Part 1. [file 13195_2022_1130_MOESM4_ESM.docx]

| **Questionnaire for Physician**  **Attributes of the Doctor in Charge and DLB Treatment Policy** |
| --- |

１．How old are you?　　　　　　　　　　　　　years

２．Which clinical department do you primarily belong to?

□　Neurology

□　Psychiatry

□　Neurosurgery

□　Geriatric medicine

□　Other　[　　　　　　　　　　　　　　　　　　　　　　　　]

３．Please select all the specialist certifications that you have earned.

□　Specialist certification by the Japan Society for Dementia Research

□　Clinical Dementia Specialist certification by the Japan Psychiatric Medical Conference

□　Specialist certification by the Japanese Psychogeriatric Society

□　Neurology specialist

□　Psychiatry specialist

□　Geriatric medicine specialist

□　Other　[　　　　　　　　　　　　　　　　　　　　　　　　]

４．How many cases of DLB have you treated?

□　<10

□　10–30

□　30–100

□　≥100

５．Please select all of the evaluation scales that you use in routine examinations for DLB.

□　MMSE

□　HDS-R

□　CDR

□　ADAS

□　NPI

□　J-ZBI

□　MDS-UPDRS (including UPDRS)

□　CFI

□　Barthel Index

□　IADL

□　Pareidolia test

□　I do not use evaluation scales in routine examinations for DLB

６．Do you refer to the Guidelines for Dementia by the Japan Society for Dementia Research in routine examination for DLB?

□　Yes

□　No

７．Do you practice off-label prescribing of medications to treat DLB?

□　Yes, often

□　Yes, sometimes

□　No

８．If your patient exhibits the same level of impairment due to hallucinations, delusions, and parkinsonism due to DLB, in light of your clinical experience to date, which symptom domains are your priority for treatment?

□　Psychiatric symptoms, such as hallucinations and delusions

□　Parkinsonism

□　Autonomic dysfunction

9. 　Please state your reason for the response selected in Q8.

□　Because it is a what the patient/caregiver(s) need

□　Other reasons

[ ]

10．Which symptoms of parkinsonism in patients with DLB would you prioritize when providing treatment in a routine examination? Please select the top three symptoms.

　　　[Options]

1. Bradykinesia/Akinesia 2. Rigidity 3. Action tremor 4. Rest tremor 5. Postural instability

　　　6. Gait disturbance (short-stepped gait) 7. Freezing of gait 8. Abnormal posture

9. Salivation 10. Fall 11. Dysphagia

Selected symptoms: 1:　　　　　　　　　2:　　　　　　　　　　3:

11．Please state the reason for the response provided in Q10.

[ 　 ]
