# Supplementary material for: Treatment needs of dementia with Lewy bodies according to patients, caregivers, and physicians: a cross-sectional, observational, questionnaire-based study in Japan
Source: Alzheimers Res Ther. 2022 Dec 15;14:188. doi: 10.1186/s13195-022-01130-4 (PMC9751509; doi:10.1186/s13195-022-01130-4)
Supplement: Supplementary file 5 — Additional file 5: Supplementary Methods 4. Questionnaire for physician: Part 2. [file 13195_2022_1130_MOESM5_ESM.docx]

Identifying code: ××-××: Questionnaire for Physician

Please answer the following questions about your patient.

1. How old is your patient?

　　　　　　　　　Years

1. Is your patient male or female?

□Male

□Female

1. How many years of education has your patient received?

※Under the pre-war education system, the years of education were as follows:

Junior high school graduate: 5 years; Girls’ high school graduate: 4 or 5 years;

Ordinary elementary school graduate and national elementary school graduate: 6 years;

Higher elementary school graduate and national secondary school graduate: 8 years.

□　　　　　　　Years □　I do not know.

1. What is the present state of rehabilitation of your patient?

□　Physiotherapy

□　Cognitive rehabilitation

□　No rehabilitation

□　I do not know

□　Other　[　　　　　　　]

1. How long have you been treating your patient? If for more than one year, please state the number of years.

□　Less than half a year

□　Between half a year and 1 year

□　One year or more →　[　　　　　　　 years]

1. Please select the first symptom exhibited by your patient.

Cognitive impairment

□ Memory impairment　□ Disorientation　□ Executive dysfunction □ Attention dysfunction

□ Fluctuating cognition　□ Visuospatial dysfunction　□ Other cognitive impairment

Parkinsonism

□ Bradykinesia/Akinesia　□ Rigidity　□ Action tremor　□ Rest tremor　□ Postural instability

□ Gait disturbance（short-stepped gait）□ Freezing of gait　□ Abnormal posture

□ Salivation　□ Fall　□ Dysphagia

Psychiatric symptoms

□ Delusions　□ Visual hallucinations　□ Hallucinations other than visual hallucinations

□ Agitation/Aggression　□ Depression　□ Anxiety　□ Apathy　□ Disinhibition

□ Aberrant motor behavior　□ Negativism □ Delirium　□ Other psychiatric symptom

Eating behavior-related problems

□ Loss of appetite　□ Increase in appetite　□ Weight loss　□ Weight gain

□ Food refusal　□ Eating non-edible things □ Unbalanced diet

Sleep-related disorders

□ Rapid eye movement sleep behavior disorder □ Daytime somnolence

□ Day–night reversal □ Night-time sleep disorder □ Sudden sleep

□ Restless legs syndrome　□ Periodic limb movement disorder

Autonomic dysfunction

□ Orthostatic hypotension　□ Disturbance of sweating 　□Constipation

□ Night-time dysuria　□ Daytime dysuria □ Syncope　□ Dizziness

Sensory disorders

□ Dysosmia

Unknown symptom

□ I do not know.

1. If you responded to Q6 regarding the first symptom that appeared, how many years ago did you observe that first symptom?

□ Less than half a year　 □ Between half a year and 1 year　□ Between 1 and 3 years

□ Between 3 and 5 years □ 5 years　or more □ I do not know.

1. How many years have passed since your patient was diagnosed with DLB?
   If it is <1 year, please fill in as (0) years and the applicable number of months.

□ (　　　) year(s) (　　　　　　) month(s)

□ I do not know

1. What was the diagnosis that your patient had received before DLB?

□ DLB was the initial diagnosis.　□ REM sleep behavior disorder　□ Alzheimer’s disease

□ Depression　□ Parkinson’s disease　□ I do not know　□ Other [　　　　 　　　]

1. Does your patient have any comorbidities and/or complications in addition to DLB?

□ Yes. □ No. □ I do not know.

1. If you selected “Yes” in Q10 regarding comorbidities and complications, please provide the names of those conditions.

[　　　　　　　　　　　　　　　　]　　[　　　　　　　　　　　　　　　　]　　[　　　　　　　　　　　　　　　　]

[　　　　　　　　　　　　　　　　]　　[　　　　　　　　　　　　　　　　]　　[　　　　　　　　　　　　　　　　]

[　　　　　　　　　　　　　　　　]　　[　　　　　　　　　　　　　　　　]　　[　　　　　　　　　　　　　　　　]

[　　　　　　　　　　　　　　　　]

1. What degree of understanding does your patient have about what you say?

□ Excellent □ Good　□ Normal □ Poor □ No understanding

1. What degree of understanding does your patient’s caregiver have about what you say?

□ Excellent　□ Good　□ Normal □ Poor □ No understanding

1. Is there someone at the hospital/clinic other than you with whom your patient can talk?

□ Yes. □ No.

1. How often do you believe is appropriate for your patient to visit you for DLB treatment?

□ Once every 2–3 weeks　□ Once a month　□ Once every 2 months

□ Once every 3 months □Other [　　　　　　　　　　　　　　　　　　　　　　]

1. When prescribing medicine, do you explain to your patient about its efficacy and side effects?

□ I always explain.　□ It depends on the medicine.　□ I do not explain.

□ Cannot provide a response

1. When prescribing medicine, do you explain to your patient’s caregiver about its efficacy and side effects?

□ I always explain. □ It depends on the medicine. □ I do not explain.

□ Cannot provide a response

1. Has your patient ever complained to you about an issue they experienced from the medicine you prescribed?

□ Yes. □ No.

1. If you selected “Yes” in Q18, please explain the issue that your patient experienced.

[ ]

1. Has your patient’s caregiver complained to you about an issue they experienced from the medicine you prescribed to your patient?

□ Yes.　□ No.

1. If you selected “Yes” in Q18, please explain the issue that your patient’s caregiver experienced.

[ ]

1. Do you believe your patient is taking medicine prescribed as instructed by you?

□ Yes.　□ No.

1. If you selected “No” in Q22, why do you believe that your patient is not taking medicine as instructed?

□ Forgot to take it.　□ Has difficulties swallowing it.

□ Considers that medicine does not work.

□ Is worried that a side effect may occur in the future.

□ I do not know.

□ Other reason 【 】

1. If you selected “Forgot to take it” in Q23, what are some of the medicines that your patient forgot to take?

Medicine name: [　　　　　　　　　　　　　　　　]　　 Medicine name: [　　　　　　　　　　　　　　　　]

Medicine name: [　　　　　　　　　　　　　　　　]　　 Medicine name: [　　　　　　　　　　　　　　　　]

Medicine name: [　　　　　　　　　　　　　　　　]

1. Do you always check leftover medicines that your patient has?

□ Yes (including in cases where I ask a pharmacist).　□ I am not aware of the details.

1. What are the medicines/dosages that you prescribed and that your patient is currently taking for cognitive impairment?

Name: [　　　　　　　　　　　　　　　　　　] Daily dose: [　　　　　　　　　　　　　　]

Name: [　　　　　　　　　　　　　　　　　　] Daily dose: [　　　　　　　　　　　　　　]

Name: [　　　　　　　　　　　　　　　　　　] Daily dose: [　　　　　　　　　　　　　　]

Name: [　　　　　　　　　　　　　　　　　　] Daily dose: [　　　　　　　　　　　　　　]

Name: [　　　　　　　　　　　　　　　　　　] Daily dose: [　　　　　　　　　　　　　　]

□ I do not prescribe any medicines for cognitive impairment.

1. What are the medicines/dosages that you prescribed and that your patient is currently taking for parkinsonism?

Name: [　　　　　　　　　　　　　　　　　　] Daily dose: [　　　　　　　　　　　　　　]

Name: [　　　　　　　　　　　　　　　　　　] Daily dose: [　　　　　　　　　　　　　　]

Name: [　　　　　　　　　　　　　　　　　　] Daily dose: [　　　　　　　　　　　　　　]

Name: [　　　　　　　　　　　　　　　　　　] Daily dose: [　　　　　　　　　　　　　　]

Name: [　　　　　　　　　　　　　　　　　　] Daily dose: [　　　　　　　　　　　　　　]

□ I do not prescribe any medicines for parkinsonism.

1. What are the medicines/dosages that you prescribed and that your patient is currently taking for BPSD (excluding sleep disorder)?

Name: [　　　　　　　　　　　　　　　　　　] Daily dose: [　　　　　　　　　　　　　　]

Name: [　　　　　　　　　　　　　　　　　　] Daily dose: [　　　　　　　　　　　　　　]

Name: [　　　　　　　　　　　　　　　　　　] Daily dose: [　　　　　　　　　　　　　　]

Name: [　　　　　　　　　　　　　　　　　　] Daily dose: [　　　　　　　　　　　　　　]

Name: [　　　　　　　　　　　　　　　　　　] Daily dose: [　　　　　　　　　　　　　　]

□　I do not prescribe any medicines for BPSD.

1. What are the medicines/dosages that you prescribed and that your patient is currently taking for sleep disorder?

Name: [　　　　　　　　　　　　　　　　　　] Daily dose: [　　　　　　　　　　　　　　]

Name: [　　　　　　　　　　　　　　　　　　] Daily dose: [　　　　　　　　　　　　　　]

Name: [　　　　　　　　　　　　　　　　　　] Daily dose: [　　　　　　　　　　　　　　]

Name: [　　　　　　　　　　　　　　　　　　] Daily dose: [　　　　　　　　　　　　　　]

Name: [　　　　　　　　　　　　　　　　　　] Daily dose: [　　　　　　　　　　　　　　]

□ I do not prescribe any medicines for sleep disorder.

1. What are the medicines/dosages that you prescribed and that your patient is currently taking for autonomic dysfunction?

Name: [　　　　　　　　　　　　　　　　　　] Daily dose: [　　　　　　　　　　　　　　]

Name: [　　　　　　　　　　　　　　　　　　] Daily dose: [　　　　　　　　　　　　　　]

Name: [　　　　　　　　　　　　　　　　　　] Daily dose: [　　　　　　　　　　　　　　]

Name: [　　　　　　　　　　　　　　　　　　] Daily dose: [　　　　　　　　　　　　　　]

Name: [　　　　　　　　　　　　　　　　　　] Daily dose: [　　　　　　　　　　　　　　]

□ I do not prescribe any medicines for autonomic dysfunction.

1. If you selected “Yes” in Q10 about comorbidities and complications, is your patient currently using medicines for comorbidities and complications?

□ Yes. □ No. □ I do not know.

1. If you selected “Yes” in Q31, please state some of those medicines; you may also send a copy of your patient’s medicine diary, if that is easier.

□ I will state the medicines.

□ I will send a copy of the medicine diary.

□ I have already sent a copy of the medicine diary.

1. If you selected “I will state the medicines” in Q32, please provide the names of the medicines prescribed for comorbidities and complications.

Name: [　　　　　　　　　　　　　　　　　　] Name: [　　　　　　　　　　　　　　　　　　]

Name: [　　　　　　　　　　　　　　　　　　] Name: [　　　　　　　　　　　　　　　　　　]

Name: [　　　　　　　　　　　　　　　　　　] Name: [　　　　　　　　　　　　　　　　　　]

Name: [　　　　　　　　　　　　　　　　　　] Name: [　　　　　　　　　　　　　　　　　　]

Name: [　　　　　　　　　　　　　　　　　　] Name: [　　　　　　　　　　　　　　　　　　]

1. Please select all the side effects due to the medicines for improving cognitive impairment previously prescribed by you for your patient.

□ No medicine for cognitive impairment has been prescribed to date.

□ No side effect has occurred to date.

□ Deterioration of cognitive function

□ Appearance or deterioration of parkinsonism

□ Appearance or deterioration of psychiatric symptoms

□ Appearance or deterioration of frequent urination or urinary incontinence

□ Cardiovascular event

□ Diarrhea

□ Nausea

□ Constipation

□ Loss of appetite

□ Dizziness or somnolence

□ Other　[　　　　　　　　　　 　　　]

1. Please select all the side effects due to the medicines for improving parkinsonism previously prescribed by you for your patient.

□ No medicine for parkinsonism has been prescribed to date.

□ No side effect has occurred to date.

□ Appearance or deterioration of hallucinations

□ Appearance or deterioration of delusions

□ Appearance or deterioration of psychiatric symptoms other than hallucinations and delusions

□ Delirium

□ Dyskinesia

□ Sleepiness

□ Insomnia

□ Weight loss

□ Loss of appetite

□ Nausea or emesis

□ Fall

□ Deterioration of cognitive function

□ Other　[ 　　　　　　　　　]

1. Please select all the side effects due to the medicines for improving BPSD (excluding sleep disorders) previously prescribed by you for your patient.

□ No medicine for BPSD has been prescribed to date.

□ No side effect has occurred to date.

□ Appearance or deterioration of parkinsonism

□ Deterioration of cognitive function

□ Delirium

□ Excessive sedation

□ Fall

□ Other　[　　　　　　　　　　　　　]

1. Please select all the side effects due to the medicines for improving sleep disorders previously prescribed by you for your patient.

□ No medicine for sleep disorders has been prescribed to date.

□ No side effect has occurred to date.

□ Difficulty falling asleep

□ Arousal during sleep

□ Excessive daytime sleepiness

□ Day–night reversal

□ Unsteady feet

□ Fall

□ Delirium

□ Deterioration of cognitive function

□ Appearance or deterioration of psychiatric symptoms

□ Appearance or deterioration of parkinsonism

□ Other　[　　 　　　　　　　　　　　]

1. Please select all the side effects due to the medicines for improving autonomic dysfunction previously prescribed by you for your patient.

□ No medicine for autonomic dysfunction has been prescribed to date.

□ No side effect has occurred to date.

□ Diarrhea

□ Constipation

□ Nausea or emesis

□ Sense of abdominal distension

□ Loss of appetite

□ Raised blood pressure

□ Palpitation

□ Dry mouth

□ Urinary disturbance

□ Deterioration of cognitive function

□ Appearance or deterioration of psychiatric symptoms

□ Appearance or deterioration of parkinsonism

□ Other　[ 　　　　　　　　　　　　]

1. Please select all of the symptoms that currently apply to your patient.

Cognitive impairment

□ Memory impairment　□ Disorientation　□ Executive dysfunction 　□ Attention dysfunction

□ Fluctuating cognition　□ Visuospatial dysfunction　□ Other cognitive impairment

Parkinsonism

□ Bradykinesia/Akinesia　□ Rigidity　□ Action tremor　□ Rest tremor　□ Postural instability

□ Gait disturbance（short-stepped gait）□ Freezing of gait　□ Abnormal posture

□ Salivation　□ Fall　□ Dysphagia

Psychiatric symptoms

□ Delusions　□ Visual hallucinations　□ Hallucinations other than visual hallucinations

□ Agitation/Aggression　□ Depression　□ Anxiety　□ Apathy　□ Disinhibition

□ Aberrant motor behavior　□ Negativism □ Delirium　□ Other psychiatric symptom

Eating behavior-related problems

□ Loss of appetite　□ Increase in appetite　□ Weight loss　□ Weight gain

□ Food refusal　□ Eating non-edible things □ Unbalanced diet

Sleep-related disorders

□ Rapid eye movement sleep behavior disorder □ Daytime somnolence

□ Day–night reversal □ Night-time sleep disorder □ Sudden sleep

□ Restless legs syndrome　□ Periodic limb movement disorder

Autonomic dysfunction

□ Orthostatic hypotension　□ Disturbance of sweating 　□ Constipation

□ Night-time dysuria　□ Daytime dysuria □ Syncope　□ Dizziness

Sensory disorders

□ Dysosmia

1. Please select all of the symptoms that currently apply to your patient receiving treatment (including pharmacological and non-pharmacological therapies) from you.

Cognitive impairment

□ Memory impairment　□ Disorientation　□ Executive dysfunction 　□ Attention dysfunction

□ Fluctuating cognition　□ Visuospatial dysfunction　□ Other cognitive impairment

Parkinsonism

□ Bradykinesia/Akinesia　□ Rigidity　□ Action tremor　□ Rest tremor　□ Postural instability

□ Gait disturbance（short-stepped gait）□ Freezing of gait　□ Abnormal posture

□ Salivation　□ Fall　□ Dysphagia

Psychiatric symptoms

□ Delusions　□ Visual hallucinations　□ Hallucinations other than visual hallucinations

□ Agitation/Aggression　□ Depression　□ Anxiety　□ Apathy　□ Disinhibition

□ Aberrant motor behavior □ Negativism　□ Delirium　□ Other psychiatric symptom

Eating behavior-related problems

□ Loss of appetite　□ Increase in appetite　□ Weight loss　□ Weight gain

□ Food refusal　□ Eating non-edible things □ Unbalanced diet

Sleep-related disorders

□ Rapid eye movement sleep behavior disorder □ Daytime somnolence

□ Day–night reversal □ Night-time sleep disorder □ Sudden sleep

□ Restless legs syndrome　□ Periodic limb movement disorder

Autonomic dysfunction

□ Orthostatic hypotension　□ Disturbance of sweating 　□ Constipation

□ Night-time dysuria　□ Daytime dysuria □ Syncope　□ Dizziness

Sensory disorders

□ Dysosmia

1. Please select only one symptom domain you think currently causes your patient the most distress.

*In this survey, psychiatric symptoms do not include eating behavior-related problems or sleep related disorders.

□ Cognitive impairment

□ Parkinsonism

□ Psychiatric symptoms

□ Eating behavior-related problems

□ Sleep-related disorders

□ Autonomic dysfunction

□ Sensory disorders (Dysosmia)

1. If you selected “Cognitive impairment” in Q41, please select only one specific symptom you think　 currently causes your patient the most distress.

Cognitive impairment

□ Memory impairment　□ Disorientation　□ Executive dysfunction □ Attention dysfunction

□ Fluctuating cognition　□ Visuospatial dysfunction　□ Other cognitive impairment

1. If you selected “Parkinsonism” in Q41, please select only one specific symptom you think　 currently causes your patient the most distress.

Parkinsonism

□ Bradykinesia/Akinesia　□ Rigidity　□ Action tremor　□ Rest tremor　□ Postural instability

□ Gait disturbance (short-stepped gait)　□ Freezing of gait　□ Abnormal posture

□ Salivation　□ Fall　□ Dysphagia

1. If you selected “Psychiatric symptoms” in Q41, please select only one specific symptom you think　 currently causes your patient the most distress.

Psychiatric symptoms

□ Delusions　□ Visual hallucinations　□ Hallucinations other than visual hallucinations

□ Agitation/Aggression　□ Depression　□ Anxiety　□ Apathy　□ Disinhibition

□ Aberrant motor behavior □ Negativism　□ Delirium　□ Other psychiatric symptom

1. If you selected “Eating behavior-related problems” in Q41, please select only one specific symptom you think currently causes your patient the most distress.

Eating behavior-related problems

□ Loss of appetite　□ Increase in appetite　□ Weight loss　□ Weight gain　□ Food refusal

□ Eating non-edible things □ Unbalanced diet

1. If you selected “Sleep-related disorders” in Q41, please select only one specific symptom you think currently causes your patient the most distress.

Sleep-related disorders

□ Rapid eye movement sleep behavior disorder　□ Daytime somnolence

□ Day–night reversal　□ Night-time sleep disorder □ Sudden sleep　□ Restless legs syndrome

□ Periodic limb movement disorder

1. If you selected “Autonomic dysfunction” in Q41, please select only one specific symptom you think currently causes your patient the most distress.

Autonomic dysfunction

□ Orthostatic hypotension　□ Disturbance of sweating 　□ Constipation

□ Night-time dysuria □ Daytime dysuria □ Syncope　□ Dizziness

1. Has your patient complained to you about the symptom that causes them the most distress (The symptom you selected in Q42–47 or dysosmia)?

□ Yes. □ No.　□ I do not know.

1. Has your patient’s caregiver complained to you about the symptom that causes your patient the most distress (The symptom you selected in Q42–47 or dysosmia)?

□ Yes.　□ No. □ I do not know.

1. Have you asked your patient about the symptom that causes them the most distress (The symptom you selected in Q42–47 or dysosmia)?

□ Yes.　□ No.　□ I do not know.

1. Have you asked your patient’s caregiver about the symptom that causes your patient the most distress (The symptom you selected in Q42–47 or dysosmia)?

□ Yes.　□ No.　□ I do not know.

1. Please select all of your patient's other symptoms other than the symptom that currently causes them the most distress.

Cognitive impairment

□ Memory impairment　□ Disorientation　□ Executive　dysfunction □ Attention dysfunction

□ Fluctuating cognition　□ Visuospatial dysfunction　□ Other cognitive impairment

Parkinsonism

□ Bradykinesia/Akinesia　□ Rigidity　□ Action tremor　□ Rest tremor　□ Postural instability

□ Gait disturbance (short-stepped gait)　□ Freezing of gait　□ Abnormal posture

□ Salivation　□ Fall　□ Dysphagia

Psychiatric symptoms

□ Delusions　□ Visual hallucinations □ Hallucinations other than visual hallucinations

□ Agitation/Aggression　□ Depression　□ Anxiety　□ Apathy　□ Disinhibition

□ Aberrant motor behavior □ Negativism　□ Delirium　□ Other psychiatric symptom

Eating behavior-related problems

□ Loss of appetite　□ Increase in appetite　□ Weight loss　□ Weight gain　□ Food refusal

□ Eating non-edible things □ Unbalanced diet

Sleep-related disorders

□ Rapid eye movement sleep behavior disorder　□ Daytime somnolence

□ Day–night reversal　□ Night-time sleep disorder □ Sudden sleep　□ Restless legs syndrome

□ Periodic limb movement disorder

Autonomic dysfunction

□ Orthostatic hypotension　□ Disturbance of sweating 　□ Constipation

□ Night-time dysuria　□ Daytime dysuria □ Syncope　□ Dizziness

Sensory disorders

- Dysosmia

Other

- No other troubling symptoms

1. Please select only one symptom domain of your patient you think currently causes your patient’s caregiver the most distress.

*In this survey, psychiatric symptoms do not include eating behavior-related problems or sleep related disorders.

□ Cognitive impairment

□ Parkinsonism

□ Psychiatric symptoms

□ Eating behavior-related problems

□ Sleep-related disorders

□ Autonomic dysfunction

□ Sensory disorders (Dysosmia)

1. If you selected “Cognitive impairment” in Q53, please select only one specific symptom of your patient you think currently causes your patient’s caregiver the most distress.

Cognitive impairment

□ Memory impairment　□ Disorientation　□ Executive dysfunction　□ Attention dysfunction

□ Fluctuating cognition　□ Visuospatial dysfunction　□ Other cognitive impairment

1. If you selected “Parkinsonism” in Q53, please select only one specific symptom of your patient you think currently causes your patient’s caregiver the most distress.

Parkinsonism

□ Bradykinesia/Akinesia　□ Rigidity　□ Action tremor　□ Rest tremor　□ Postural instability

□ Gait disturbance (short-stepped gait)　□ Freezing of gait　□ Abnormal posture

□ Salivation　□ Fall　□ Dysphagia

1. If you selected “Psychiatric symptoms” in Q53, please select only one specific symptom of your patient you think currently causes your patient’s caregiver the most distress.

Psychiatric symptoms

□ Delusions　□ Visual hallucinations　□ Hallucinations other than visual hallucinations

□ Agitation/Aggression　□ Depression　□ Anxiety　□ Apathy　□ Disinhibition

□ Aberrant motor behavior □ Negativism　□ Delirium　□ Other psychiatric symptom

1. If you selected “Eating behavior-related problems” in Q53, please select only one specific symptom of your patient you think currently causes your patient’s caregiver the most distress.

Eating behavior-related problems

□ Loss of appetite　□ Increase in appetite　□ Weight loss　□ Weight gain　□ Food refusal

□ Eating non-edible things □ Unbalanced diet

1. If you selected “Sleep-related disorders” in Q53, please select only one specific symptom of your patient you think currently causes your patient’s caregiver the most distress.

Sleep-related disorders

□ Rapid eye movement sleep behavior disorder　□ Daytime somnolence

□ Day–night reversal　□ Night-time sleep disorder □ Sudden sleep　□ Restless legs syndrome

□ Periodic limb movement disorder

1. If you selected “Autonomic dysfunction” in Q53, please select only one specific symptom of your patient you think currently causes your patient’s caregiver the most distress.

Autonomic dysfunction

□ Orthostatic hypotension　□ Disturbance of sweating □ Constipation

□ Night-time dysuria　□ Daytime dysuria □ Syncope　□ Dizziness

1. Has your patient’s caregiver complained to you about the symptom that causes them the most distress (The symptom you selected in Q54–59 or dysosmia)?

□ Yes.　□ No. □ I do not know.

1. Have you asked your patient’s caregiver about the symptom that causes them the most distress (The symptom you selected in Q54–59 or dysosmia)?

□ Yes.　□ No.　□ I do not know.

1. Please select all of your patient's other specific symptoms you think currently causes your patient’s caregiver the distress other that the symptom that causes them the most distress.

Cognitive impairment

□ Memory impairment　□ Disorientation　□ Executive　dysfunction □ Attention dysfunction

□ Fluctuating cognition　□ Visuospatial dysfunction □ Other cognitive impairment

Parkinsonism

□ Bradykinesia/Akinesia　□ Rigidity　□ Action tremor　□ Rest tremor　□ Postural instability

□ Gait disturbance (short-stepped gait)　□ Freezing of gait　□ Abnormal posture

□ Salivation　□ Fall　□ Dysphagia

Psychiatric symptoms

□ Delusions　□ Visual hallucinations　□ Hallucinations other than visual hallucinations

□ Agitation/Aggression　□ Depression　□ Anxiety　□ Apathy　□ Disinhibition

□ Aberrant motor behavior □ Negativism　□ Delirium □ Other psychiatric symptom

Eating behavior-related problems

□ Loss of appetite　□ Increase in appetite　□ Weight loss　□ Weight gain　□ Food refusal

□ Eating non-edible things □ Unbalanced diet

Sleep-related disorders

□ Rapid eye movement sleep behavior disorder　□ Daytime somnolence

□ Day–night reversal　□ Night-time sleep disorder □ Sudden sleep　□ Restless legs syndrome

□ Periodic limb movement disorder

Autonomic dysfunction

□ Orthostatic hypotension □ Disturbance of sweating □ Constipation

□ Night-time dysuria　□ Daytime dysuria □ Syncope □ Dizziness

Sensory disorders

- Dysosmia

Other

- No other troubling symptoms

1. Are there any symptoms that you suspect your patient is experiencing but are unable to treat owing to a lack of effective therapy?

□ Yes.（→Go to Q64）□ No.（→Go to 65）

1. If you selected “Yes” in Q63, what are those specific symptoms? Please select all applicable symptoms.

Cognitive impairment

□ Memory impairment　□ Disorientation　□ Executive　dysfunction □ Attention dysfunction

□ Fluctuating cognition　□ Visuospatial dysfunction　□ Other cognitive impairment

Parkinsonism

□ Bradykinesia/Akinesia　□ Rigidity　□ Action tremor　□ Rest tremor　□ Postural instability

□ Gait disturbance (short-stepped gait)　□ Freezing of gait　□ Abnormal posture

□ Salivation □ Fall　□ Dysphagia

Psychiatric symptoms

□ Delusions　□ Visual hallucinations　□ Hallucinations other than visual hallucinations

□ Agitation/Aggression　□ Depression　□ Anxiety　□ Apathy　□ Disinhibition

□ Aberrant motor behavior □ Negativism　□ Delirium □ Other psychiatric symptom

Eating behavior-related problems

□ Loss of appetite　□ Increase in appetite　□ Weight loss　□ Weight gain　□ Food refusal

□ Eating non-edible things □ Unbalanced diet

Sleep-related disorders

□ Rapid eye movement sleep behavior disorder　□ Daytime somnolence

□ Day–night reversal　□ Night-time sleep disorder □ Sudden sleep　□ Restless legs syndrome

□ Periodic limb movement disorder

Autonomic dysfunction

□ Orthostatic hypotension　□ Disturbance of sweating □ Constipation

□ Night-time dysuria　□ Daytime dysuria □ Syncope　□ Dizziness

Sensory disorders

□ Dysosmia

1. Are there any symptoms that you suspect your patient is experiencing but are unable to treat because of a potential deterioration of existing symptoms despite the availability of therapies?

□ Yes.（→Go to Q66）□ No.（→GO to Q67）

1. If you selected “Yes” in Q65, what are those specific symptoms? Please select all applicable symptoms.

Cognitive impairment

□ Memory impairment　□ Disorientation　□ Executive　dysfunction □ Attention dysfunction

□ Fluctuating cognition　□ Visuospatial dysfunction　□ Other cognitive impairment

Parkinsonism

□ Bradykinesia/Akinesia　□ Rigidity　□ Action tremor　□ Rest tremor　□ Postural instability

□ Gait disturbance (short-stepped gait)　□ Freezing of gait　□ Abnormal posture

□ Salivation　□ Fall　□ Dysphagia

Psychiatric symptoms

□ Delusions　□ Visual hallucinations　□ Hallucinations other than visual hallucinations

□ Agitation/Aggression　□ Depression　□ Anxiety　□ Apathy　□ Disinhibition

□ Aberrant motor behavior □ Negativism　□ Delirium　□ Other psychiatric symptom

Eating behavior-related problems

□ Loss of appetite　□ Increase in appetite　□ Weight loss　□ Weight gain　□ Food refusal

□ Eating non-edible things □ Unbalanced diet

Sleep-related disorders

□ Rapid eye movement sleep behavior disorder　□ Daytime somnolence

□ Day–night reversal　□ Night-time sleep disorder □ Sudden sleep　□ Restless legs syndrome

□ Periodic limb movement disorder

Autonomic dysfunction

□ Orthostatic hypotension　□ Disturbance of sweating □ Constipation

□ Night-time dysuria　□ Daytime dysuria □ Syncope　□ Dizziness

Sensory disorders

- Dysosmia

1. As your patient continues with treatment, which symptom do you think they would most likely prioritize for receiving treatment? Please select only one applicable symptom domain.

*In this survey, psychiatric symptoms do not include eating behavior-related problems or sleep-related disorders.

□ Cognitive impairment

□ Parkinsonism

□ Psychiatric symptoms

□ Eating behavior-related problems

□ Sleep-related disorders

□ Autonomic dysfunction

□ Dysosmia

1. If you selected “Cognitive impairment” in Q67, as your patient continues with treatment, which symptom do you think they would most likely prioritize for receiving treatment? Please select only one specific symptom.

Cognitive impairment

□ Memory impairment　□ Disorientation　□ Executive dysfunction □ Attention dysfunction

□ Fluctuating cognition　□ Visuospatial dysfunction　□ Other cognitive impairment

1. If you selected “Parkinsonism” in Q67, as your patient continues with treatment, which symptom do you think they would most likely prioritize for receiving treatment? Please select only one specific symptom.

Parkinsonism

□ Bradykinesia/Akinesia　□ Rigidity　□ Action tremor　□ Rest tremor　□ Postural instability

□ Gait disturbance (short-stepped gait)　□ Freezing of gait　□ Abnormal posture

□ Salivation　□Fall　□Dysphagia

1. If you selected “Psychiatric symptoms” in Q67, as your patient continues with treatment, which symptom do you think they would most likely prioritize for receiving treatment? Please select only one specific symptom.

Psychiatric symptoms

□ Delusions　□ Visual hallucinations　□ Hallucinations other than visual hallucinations

□ Agitation/Aggression　□ Depression　□ Anxiety　□ Apathy　□ Disinhibition

□ Aberrant motor behavior □ Negativism　□ Delirium　□ Other psychiatric symptom

1. If you selected “Eating behavior-related problems” in Q67, as your patient continues with treatment, which symptom do you think they would most likely prioritize for receiving treatment? Please select only one specific symptom.

Eating behavior-related problems

□ Loss of appetite　□ Increase in appetite　□ Weight loss　□ Weight gain　□ Food refusal

□ Eating non-edible things □ Unbalanced diet

1. If you selected “Sleep-related disorders” in Q67, as your patient continues with treatment, which symptom do you think they would most likely prioritize for receiving treatment? Please select only one specific symptom.

Sleep-related disorders

□ Rapid eye movement sleep behavior disorder　□ Daytime somnolence

□ Day–night reversal　□ Night-time sleep disorder □ Sudden sleep　□ Restless legs syndrome

□ Periodic limb movement disorder

1. If you selected “Autonomic dysfunction” in Q67, as your patient continues with treatment, which symptom do you think they would most likely prioritize for receiving treatment? Please select only one specific symptom.

Autonomic dysfunction

□ Orthostatic hypotension　□ Disturbance of sweating □ Constipation

□ Night-time dysuria　□ Daytime dysuria □ Syncope　□ Dizziness

1. As your patient continues with treatment, what is the scheduled course of treatment for the symptom that they would most likely prioritize for receiving treatment (selected in Q68–73 or dysosmia)?

□ Pharmacological therapy (Go to Q75)

□ Non-pharmacological therapy (Go to Q77)

□ Unable to treat (Go to Q76)

□ Other (　　　　　　　　　　　　　　　　　　　　　　　　　　) (Go to Q77)

1. If you selected “Pharmacological therapy” in Q74, please state the medicine that would be used in pharmacological therapy.

Name: [ ]

1. If you selected “Unable to treat” in Q74, please state the reason for the inability to treat the symptom.

Reason: [ ]

1. As your patient continues with treatment, which patient’s symptom do you think your patient’s caregiver would most likely prioritize for receiving treatment? Please select only one applicable symptom domain.

*In this survey, psychiatric symptoms do not include eating behavior-related problems or sleep-related disorders.

□ Cognitive impairment

□ Parkinsonism

□ Psychiatric symptoms

□ Eating behavior-related problems

□ Sleep-related disorders

□ Autonomic dysfunction

□ Dysosmia

1. If you selected “Cognitive impairment” in Q77, as your patient continues with treatment, which patient’s symptom do you think your patient’s caregiver would most likely prioritize for receiving treatment? Please select only one specific symptom.

Cognitive impairment

□ Memory impairment　□ Disorientation　□ Executive dysfunction □ Attention dysfunction

□ Fluctuating cognition　□ Visuospatial dysfunction　□ Other cognitive impairment

1. If you selected “Parkinsonism” in Q77, as your patient continues with treatment, which patient’s symptom do you think your patient’s caregiver would most likely prioritize for receiving treatment? Please select only one specific symptom.

Parkinsonism

□ Bradykinesia/Akinesia　□ Rigidity　□ Action tremor　□ Rest tremor　□ Postural instability

□ Gait disturbance (short-stepped gait)　□ Freezing of gait　□ Abnormal posture

□ Salivation　□ Fall　□ Dysphagia

1. If you selected “Psychiatric symptoms” in Q77, as your patient continues with treatment, which patient’s symptom do you think your patient’s caregiver would most likely prioritize for receiving treatment? Please select only one specific symptom.

Psychiatric symptoms

□ Delusions　□ Visual hallucinations　□ Hallucinations other than visual hallucinations

□ Agitation/Aggression　□ Depression　□ Anxiety　□ Apathy　□ Disinhibition

□ Aberrant motor behavior □ Negativism　□ Delirium □ Other psychiatric symptom

1. If you selected “Eating behavior-related problems” in Q77, as your patient continues with treatment, which patient’s symptom do you think your patient’s caregiver would most likely prioritize for receiving treatment? Please select only one specific symptom.

Eating behavior-related problems

□ Loss of appetite　□ Increase in appetite　□ Weight loss　□ Weight gain　□ Food refusal

□ Eating non-edible things □ Unbalanced diet

1. If you selected “Sleep-related disorders” in Q77, as your patient continues with treatment, which patient’s symptom do you think your patient’s caregiver would most likely prioritize for receiving treatment? Please select only one specific symptom.

Sleep-related disorders

□ Rapid eye movement sleep behavior disorder　□ Daytime somnolence

□ Day–night reversal　□ Night-time sleep disorder □ Sudden sleep　□ Restless legs syndrome

□ Periodic limb movement disorder

1. If you selected “Autonomic dysfunction” in Q77, as your patient continues with treatment, which patient’s symptom do you think your patient’s caregiver would most likely prioritize for receiving treatment? Please select only one specific symptom.

Autonomic dysfunction

□ Orthostatic hypotension　□ Disturbance of sweating □ Constipation

□ Night-time dysuria　□ Daytime dysuria □ Syncope　□ Dizziness

1. As your patient continues with treatment, what is the scheduled course of treatment for the symptom that your patient’s caregiver would most likely prioritize for receiving treatment (selected in Q78–83 or dysosmia)?

□　Pharmacological therapy（Go to Q85）

□　Non-pharmacological therapy (Go to Q87)

□　Unable to treat (Go to Q86)

□　Other (　　　　　　　　　　　　　　　　　　　　　　　　　　) (Go to Q87)

1. If you selected “Pharmacological therapy” in Q84, please state the medicine that would be used in pharmacological therapy.

Name: [ ]

1. If you selected “Unable to treat” in Q84, please state the reason for the inability to treat the symptom.

Reason: [ ]

Please confirm your response to Q39.

Did you select any of the following?　“Psychiatric symptoms”, “Eating behavior-related problems”, or “Sleep-related disorders”

□ Yes.（→Go to Q87）□ No.（→Go to Q98）

1. Did you explain to your patient about psychiatric symptoms, eating behavior-related problems, and sleep-related disorders?

□ I explained. □ I explained partially.　□ I did not explain.

1. Did you explain to your patient’s caregiver about psychiatric symptoms, eating behavior-related problems, and sleep-related disorders?

□ I explained.　□ I explained partially.　□ I did not explain.

1. If you selected psychiatric symptoms, eating behavior-related problems, and sleep-related disorders in Q39, which is the symptom classified under these three categories do you think currently causes your patient the most distress? Please select only one applicable symptom.

Psychiatric symptoms

□ Delusions　□ Visual hallucinations　□ Hallucinations other than visual hallucinations

□ Agitation/Aggression　□ Depression　□ Anxiety　□ Apathy　□ Disinhibition

□ Aberrant motor behavior □ Negativism　□ Delirium　□ Other psychiatric symptom

Eating behavior-related problems

□ Loss of appetite　□ Increase in appetite　□ Weight loss　□ Weight gain　□ Food refusal

□ Eating non-edible things □ Unbalanced diet

Sleep-related disorders

□ Rapid eye movement sleep behavior disorder　□ Daytime somnolence

□ Day–night reversal　□ Night-time sleep disorder □ Sudden sleep　□ Restless legs syndrome

□ Periodic limb movement disorder

1. If you selected psychiatric symptoms, eating behavior-related problems, and sleep-related disorders in Q39, are you currently treating (using pharmacological or non- pharmacological therapy) the symptom that causes your patient the most distress (selected in Q89)?

□ Yes.（→Go to Q92）□ No.（→Go to Q91）

1. If you selected “No” in Q90, please state the reason for not treating the symptom that causes your patient the most distress (selected in Q89).

□ Treatment will affect your patient’s physical condition and cause complications.

□ Your patient has other psychiatric symptom, eating behavior-related problem, or sleep-related disorder that requires treatment.（→Add Q95）

□ It is necessary to avoid a deterioration of parkinsonism.

□ The priority is to treat parkinsonism.

□ The symptom has not reached a level where treatment is required.

□ There are no effective medicines.

□ Other　[　　　　　　　　　　　　　　　　　　　　　　　　　　　　　　　　　　　　　　　]

1. If you selected psychiatric symptoms, eating behavior-related problems, and sleep-related disorders in Q39, which is the patient’s symptom classified under these three categories do you think currently causes your patient’s caregiver the most distress? Please select only one applicable symptom.

Psychiatric symptoms

□ Delusions　□ Visual hallucinations　□ Hallucinations other than visual hallucinations

□ Agitation/Aggression　□ Depression　□ Anxiety　□ Apathy　□ Disinhibition

□ Aberrant motor behavior □ Negativism　□ Delirium　□ Other psychiatric symptom

Eating behavior-related problems

□ Loss of appetite　□ Increase in appetite　□ Weight loss　□ Weight gain　□ Food refusal

□ Eating non-edible things □ Unbalanced diet

Sleep-related disorders

□ Rapid eye movement sleep behavior disorder　□ Daytime somnolence

□ Day–night reversal　□ Night-time sleep disorder □ Sudden sleep　□ Restless legs syndrome

□ Periodic limb movement disorder

1. If you selected psychiatric symptoms, eating behavior-related problems, and sleep-related disorders in Q39, are you currently treating (using pharmacological or non-pharmacological therapy) the symptom that causes your patient’s caregiver the most distress (selected in Q92)?

□ Yes.（→Go to Q96）□ No.（→Go to Q94）

1. If you selected “No” in Q93, please state the reason for not treating the symptom that causes your patient’s caregiver the most distress (selected in Q93)?

□ Treatment will affect your patient’s physical condition and cause complications.

□ Your patient has other psychiatric symptom, eating behavior-related problem, or sleep-related disorder that requires treatment.（→ Add Q95）

□ It is necessary to avoid a deterioration of parkinsonism.

□ The priority is to treat parkinsonism.

□ The symptom has not reached a level where treatment is required.

□ There are no effective medicines.

□ Other　[　　　　　　　　　　　　　　　　　　　　　　　　　　　　　　　　　　　　　　　]

1. If you selected “Your patient has other psychiatric symptom, eating behavior-related problem, or sleep-related disorder that requires treatment.” in Q91 or Q94, please select only one psychiatric symptom, eating behavior-related problem, or sleep-related disorder of your patient that should be treated with priority.

Psychiatric symptoms

□ Delusions　□ Visual hallucinations　□ Hallucinations other than visual hallucinations

□ Agitation/Aggression　□ Depression　□ Anxiety　□ Apathy　□ Disinhibition

□ Aberrant motor behavior □ Negativism　□ Delirium　□ Other psychiatric symptom

Eating behavior-related problems

□ Loss of appetite　□ Increase in appetite　□ Weight loss　□ Weight gain　□ Food refusal

□ Eating non-edible things □ Unbalanced diet

Sleep-related disorders

□ Rapid Eye Movement sleep behavior disorder　□ Daytime somnolence

□ Day–night reversal　□ Night-time sleep disorder □ Sudden sleep　□ Restless legs syndrome

□ Periodic limb movement disorder

1. If you selected psychiatric symptoms, eating behavior-related problems, and sleep-related disorders in Q39, as your patient continues with treatment, which of these symptoms do you think they would likely prioritize for receiving treatment? Please select all applicable symptoms.

Psychiatric symptoms

□ Delusions　□ Visual hallucinations　□ Hallucinations other than visual hallucinations

□ Agitation/Aggression　□ Depression　□ Anxiety　□ Apathy　□ Disinhibition

□ Aberrant motor behavior □ Negativism　□ Delirium　□ Other psychiatric symptom

Eating behavior-related problems

□ Loss of appetite　□ Increase in appetite　□ Weight loss　□ Weight gain　□ Food refusal

□ Eating non-edible things □ Unbalanced diet

Sleep-related disorders

□ Rapid Eye Movement sleep behavior disorder　□ Daytime somnolence

□ Day–night reversal　□ Night-time sleep disorder □ Sudden sleep　□ Restless legs syndrome

□ Periodic limb movement disorder

1. If you selected psychiatric symptoms, eating behavior-related problems, and sleep-related disorders in Q39, as your patient continues with treatment, which of these your patient’s symptoms do you think your patient’s caregiver would likely prioritize for receiving treatment? Please select all applicable symptoms.

Psychiatric symptoms

□ Delusions　□ Visual hallucinations　□ Hallucinations other than visual hallucinations

□ Agitation/Aggression　□ Depression　□ Anxiety　□ Apathy　□ Disinhibition

□ Aberrant motor behavior □ Negativism　□ Delirium　□ Other psychiatric symptom

Eating behavior-related problems

□ Loss of appetite　□ Increase in appetite　□ Weight loss　□ Weight gain　□ Food refusal

□ Eating non-edible things □ Unbalanced diet

Sleep-related disorders

□ Rapid Eye Movement sleep behavior disorder　□ Daytime somnolence

□ Day–night reversal　□ Night-time sleep disorder □ Sudden sleep　□ Restless legs syndrome

□ Periodic limb movement disorder

Please confirm your response to Q39.

Did you select any items of Parkinsonism?

□ Yes.（→Go to Q98）□ No.（→Go to Q110）

1. Did you explain to your patient about parkinsonism?

□ I explained.　□ I explained partially.　□ I did not explain.

1. Did you explain to your patient’s caregiver about parkinsonism?

□ I explained.　□ I explained partially.　□ I did not explain.

1. If you prescribed a levodopa formulation for your patient, please select your patient’s response.

□ It was extremely effective.

□ It was moderately effective.

□ No change was observed.

□ It was not very effective.

□ It was not effective at all.

□ I have not prescribed it.

1. If you selected any items of Parkinsonism in Q39, which symptom of Parkinsonism do you think causes your patient the most distress? Please select only one applicable symptom.

□ Bradykinesia/Akinesia　□ Rigidity　□ Action tremor　□ Rest tremor　□ Postural instability

□ Gait disturbance (short-stepped gait)　□ Freezing of gait　□ Abnormal posture

□ Salivation　□ Fall　□ Dysphagia

1. If you selected any items of Parkinsonism in Q39, are you currently treating (using pharmacological or non-pharmacological therapy) the symptom that causes your patient the most distress (selected in Q101)?

□ Yes.（→Go to Q104）□ No.（→Go to Q103）

1. If you selected “No” in Q102, please state the reason for not treating the symptom that causes your patient the most distress (selected in Q101).

□ Treatment will affect your patient’s physical condition or cause complications.

□ Your patient has other symptom of parkinsonism that requires treatment.（→Add Q107）

□ It is necessary to avoid a deterioration of psychiatric symptoms.

□ The priority is to treat psychiatric symptoms.

□ The symptom has not reached a level where treatment is required.

□ There are no effective medicines.

□ Other [　　　　　　　　　　　　　　　　　　　　　　　　　　　　　　　　　　　　　　　]

1. If you selected any items of Parkinsonism in Q39, which patient’s symptom of Parkinsonism do you think currently causes your patient caregiver the most distress? Please select only one applicable symptom.

□ Bradykinesia/Akinesia　□ Rigidity　□ Action tremor　□ Rest tremor　□ Postural instability

□ Gait disturbance (short-stepped gait)　□ Freezing of gait　□ Abnormal posture

□ Salivation　□ Fall　□ Dysphagia

1. If you selected any items of Parkinsonism in Q39, are you currently treating (pharmacological and non-pharmacological therapy) the symptom that causes your patient’s caregiver the most distress (selected in Q104)?

□Yes.（→Go to Q108）□No.（→Go to Q106）

1. If you selected “No” in Q105, please state the reason for not treating the symptom that causes your patient’s caregiver the most distress (selected in Q104)?

□ Treatment will affect your patient’s physical condition or cause complications.

□ Your patient has other symptom of parkinsonism that requires treatment.（→Add Q107）

□ It is necessary to avoid a deterioration of psychiatric symptoms.

□ The priority is to treat psychiatric symptoms.

□ The symptom has not reached a level where treatment is required.

□ There are no effective medicines.

□ Other　[　　　　　　　　　　　　　　　　　　　　　　　　　　　　　　　　　　　　　　　]

1. If you selected “Your patient has other symptom of parkinsonism that requires treatment .” in Q103 or Q106, please select only one parkinsonism of your patient that should be treated with priority.

□ Bradykinesia/Akinesia　□ Rigidity　□ Action tremor　□ Rest tremor　□ Postural instability

□ Gait disturbance (Short-stepped gait)　□ Freezing of gait　□ Abnormal posture

□ Salivation　□ Fall　□ Dysphagia

1. If you selected any items of Parkinsonism in Q39, as your patient continues with treatment, which symptoms of parkinsonism do you think they would likely prioritize for receiving treatment? Please select all applicable symptoms.

□ Bradykinesia/Akinesia　□ Rigidity　□ Action tremor　□ Rest tremor　□ Postural instability

□ Gait disturbance (short-stepped gait)　□ Freezing of gait　□ Abnormal posture

□ Salivation　□ Fall　□ Dysphagia

1. If you selected any items of Parkinsonism in Q39, as your patient continues with treatment, which patient’s symptoms of parkinsonism do you think your patient’s caregiver would likely prioritize for receiving treatment? Please select all applicable symptoms.

□ Bradykinesia/Akinesia　□ Rigidity　□ Action tremor　□ Rest tremor　□ Postural instability

□ Gait disturbance (short-stepped gait)　□ Freezing of gait　□ Abnormal posture

□ Salivation　□ Fall　□ Dysphagia

1. Please select the degree of satisfaction in terms of the treatment effect of the medication currently prescribed for cognitive impairment to your patient.

□ I am extremely satisfied.　□ I am moderately satisfied. □ It is difficult to select either.

□ I am slightly dissatisfied.　□ I am extremely dissatisfied.

□ I do not prescribe any medicines for cognitive impairment.

1. Please select the degree of satisfaction in terms of the treatment effect of the medication currently prescribed for parkinsonism to your patient.

□ I am extremely satisfied.　□ I am moderately satisfied. □ It is difficult to select either.

□ I am slightly dissatisfied.　□ I am extremely dissatisfied.

□ I do not prescribe any medicines for parkinsonism.

1. Please select the degree of satisfaction in terms of the treatment effect of the medication currently prescribed for psychiatric symptoms to your patient (excluding sleep disorders).

□ I am extremely satisfied. □ I am moderately satisfied. □ It is difficult to select either.

□ I am slightly dissatisfied.　□ I am extremely dissatisfied.

□ I do not prescribe any medicines for psychiatric symptoms.

1. Please select the degree of satisfaction in terms of the treatment effect of the medication currently prescribed for sleep-related disorders to your patient.

□ I am extremely satisfied. □ I am moderately satisfied. □ It is difficult to select either.

□ I am slightly dissatisfied.　□ I am extremely dissatisfied.

□ I do not prescribe any medicines for sleep-related disorders.

1. Please select the degree of satisfaction in terms of the treatment effect of the medication currently prescribed for autonomic dysfunction to your patient.

□ I am extremely satisfied. □ I am moderately satisfied. □ It is difficult to select either.

□ I am slightly dissatisfied.　□ I am extremely dissatisfied.

□ I do not prescribe any medicines for autonomic dysfunction.

The questionnaire is complete.

Thank you for your cooperation.
