# Supplementary material for: Treatment needs of dementia with Lewy bodies according to patients, caregivers, and physicians: a cross-sectional, observational, questionnaire-based study in Japan
Source: Alzheimers Res Ther. 2022 Dec 15;14:188. doi: 10.1186/s13195-022-01130-4 (PMC9751509; doi:10.1186/s13195-022-01130-4)
Supplement: Supplementary file 6 — Additional file 6: Supplementary Methods 5. Other indicators assessed for concordance rate. [file 13195_2022_1130_MOESM6_ESM.docx]

**Supplementary Methods 5**

Details of the methods used for univariate analysis

In the univariate analyses between patients and physicians and between caregivers and physicians, the common explanatory variables were as follows:

Patient factors were 1. patient’s age (second part of questionnaire for physician Q1), 2. patient’s sex (second part of questionnaire for physician Q2), 3. duration of dementia with Lewy bodies (DLB) (second part of questionnaire for physician Q8), 4. duration of education (second part of questionnaire for physician Q3), 5. number of persons living with the patient (if living at home) (questionnaire for caregiver Q7), 6. patient’s knowledge of DLB (questionnaire for patient Q1), 7. frequency of hospital or clinic visits (questionnaire for caregiver Q23), 8. facility use (long-term care, outpatient rehabilitation, multifunctional home care) (questionnaire for caregiver Q8, 9, 10, and 11), 9. patient’s understanding from physician’s point of view (second part of questionnaire for physician Q12), 10. patient’s initial symptom domain (the second part of questionnaire for physician Q6), 11. duration from presenting the initial symptom domain (second part of questionnaire for physician Q7), 12. the Japanese version of the Mini-Mental State Examination total score (MMSE-J), 13. Japanese version of the Movement Disorder Society-Unified Parkinson’s Disease Rating Scale (MDS-UPDRS) Part III total score, 14. Neuropsychiatric Inventory (NPI)-10 score, 15. NPI-12 subitem score of “Nighttime Behavior score”, 16. NPI-12 subitem score of “Appetite”, 17. presence of autonomic dysfunction (if autonomic dysfunction is checked in the second part of questionnaire for physician Q39), 18. presence of sensory disorders (if dysosmia is checked in the second part of questionnaire for physician Q39), 19. MDS-UPDRS Part II total score, 20. Cognitive Fluctuation Inventory score (CFI), 21. presence or absence of pharmacotherapy for cognitive impairment (second part of questionnaire for physician Q26), 22. presence or absence of pharmacotherapy for parkinsonism (second part of questionnaire for physician Q27), 23. presence or absence of pharmacotherapy for psychiatric symptoms (second part of questionnaire for physician Q28), 24. presence or absence of pharmacotherapy for sleep-related disorders (second part of questionnaire for physician Q29), 25. presence or absence of pharmacotherapy for autonomic dysfunction (second part of questionnaire for physician Q30), 26. from the physician’s perspective, whether there is someone at the hospital/clinic other than the physician with whom the patient can talk (second part of questionnaire for physician Q14), 27. appropriate frequency of hospital or clinic visits of patient from the physician’s perspective (second part of questionnaire for physician Q15).

Caregiver factors were 1. caregiver’s age (questionnaire for caregiver Q12), 2. caregiver’s sex (questionnaire for caregiver Q13), 3. caregiver’s knowledge of DLB (questionnaire for caregiver Q25), 4. conditions of caregiver’s job (questionnaire for caregiver Q14), 5. presence of assistant caregivers (questionnaire for caregiver Q18), 6. relationship with the patient from patient’s perspective (questionnaire for caregiver Q17), 7. whether caregiver lives with the patient (questionnaire for caregiver Q15), 8. spending time with the patient each day (If caregiver lives with the patient, questionnaire for caregiver Q16), 9. frequency of hospital or clinic visits desired by caregiver (questionnaire for caregiver Q24), 10. caregiver’s understanding from physician’s point of view (second part of questionnaire for physician Q13), 11. shortened Japanese version of the Zarit Caregiver Burden Interview (J-ZBI-8) score, 12. from the caregiver’s perspective, whether the patient’s physician listens to what the caregiver says (questionnaire for caregiver Q26), 13. from the caregiver’s perspective, whether there is someone at the hospital or clinic other than the patient’s physician with whom the caregiver can talk (questionnaire for caregiver Q27).

Physician factors were 1. physician’s age (first part of questionnaire for physician Q1), 2. physician’s sex (male or female), 3. number of DLB patients treated to date (first part of questionnaire for physician Q4), 4. symptom domains prioritized for treatment (if patients exhibits hallucinations, delusions, and parkinsonism due to DLB were at the same level) (first part of questionnaire for physician Q8), 5. clinical department which physician is affiliated with (psychiatric or non-psychiatric), 6. institute (university hospital, non-university hospital, or clinic), 7. whether the Guidelines for Dementia by the Japan Society for Dementia Research were referred to (first part of questionnaire for physician Q6), 8. frequency of off-label prescribing of medications (first part of questionnaire for physician Q7), 9. duration of the patient treatment (second part of questionnaire for physician Q5).

In the univariate analyses between patients and physicians, the following five patient factors and one caregiver factor were used. The patient factors were: 1. from the patient’s perspective, whether the patient’s physician listens to what the patient says (questionnaire for patient Q2), 2. from the patient’s perspective, whether the patient has someone at the hospital or clinic other than the patient’s physician with whom the patient can talk (questionnaire for patient Q3), 3. symptom domain that causes the patient most distress (questionnaire for patient Q13), 4. whether the patient told the physician about which symptom domain causes the patient most distress (questionnaire for patient Q15), 5. whether the patient told the caregiver or family member about which symptom domain causes the patient most distress (questionnaire for patient Q17). The caregiver factor was 1. symptom domain that the caregiver selected as causing the patient the most distress (questionnaire for caregiver Q45).

In the univariate analyses between caregivers and physicians, the following two caregiver factors were used: 1. patient’s symptom domain that causes the caregiver most distress (questionnaire for caregiver Q36) and 2. whether the caregiver informed the physician about which patient’s symptom domain causes the caregiver most distress (questionnaire for caregiver Q38). Continuous variables were classified as above or below the median value.
