# Supplementary material for: Treatment needs of dementia with Lewy bodies according to patients, caregivers, and physicians: a cross-sectional, observational, questionnaire-based study in Japan
Source: Alzheimers Res Ther. 2022 Dec 15;14:188. doi: 10.1186/s13195-022-01130-4 (PMC9751509; doi:10.1186/s13195-022-01130-4)
Supplement: Supplementary file 7 — Additional file 7: Supplementary Table 2. Symptoms that cause the patient or caregiver distress. [file 13195_2022_1130_MOESM7_ESM.docx]

**Supplementary Table 2** **Symptoms that cause the patient or caregiver distress (multiple answers allowed)**

| **Patient (*N* = 263)** | | **Caregiver (*N* = 263)** | |
| --- | --- | --- | --- |
| **Symptom domain** | ***n* (%)** | **Symptom domain** | ***n* (%)** |
| Parkinsonism | 147 (55.9) | Cognitive impairment | 184 (70.0) |
| Cognitive impairment | 142 (54.0) | Psychiatric symptoms | 174 (66.2) |
| Autonomic dysfunction | 134 (51.0) | Parkinsonism | 160 (60.8) |
| Sleep-related disorders | 114 (43.3) | Autonomic dysfunction | 149 (56.7) |
| Psychiatric symptoms | 108 (41.1) | Sleep-related disorders | 137 (52.1) |
| Eating behavior-related problems | 67 (22.5) | Eating behavior-related problems | 91 (34.6) |
| Sensory disorders | 28 (10.6) | Sensory disorders | 29 (11.0) |
| **Symptom** | ***n* (%)** | **Symptom** | ***n* (%)** |
| Memory impairment | 103 (39.2) | Memory impairment | 110 (41.8) |
| Constipation | 86 (32.7) | Bradykinesia/akinesia | 102 (38.8) |
| Bradykinesia/akinesia | 83 (31.6) | Visual hallucinations | 89 (33.8) |
| Postural instability | 63 (24.0) | Constipation | 82 (31.2) |
| Gait disturbance | 61 (23.2) | Nighttime dysuria | 75 (28.5) |
| Nighttime dysuria | 58 (22.1) | Daytime somnolence | 73 (27.8) |
| Visual hallucinations | 52 (19.8) | Postural instability | 71 (27.0) |
| Abnormal posture | 50 (19.0) | Gait disturbance | 70 (26.6) |
| Daytime somnolence | 50 (19.0) | Executive dysfunction | 66 (25.1) |
| Executive dysfunction | 50 (19.0) | Attention dysfunction | 61 (23.2) |
| Attention dysfunction | 48 (18.3) | Fluctuating cognition | 58 (22.1) |
| Nighttime sleep disorder | 48 (18.3) | Disorientation | 55 (20.9) |
| Freezing of gait | 47 (17.9) | Rapid eye movement sleep behavior disorder | 53 (20.2) |
| Depression | 45 (17.1) | Apathy | 51 (19.4) |
| Action tremor | 43 (16.3) | Depression | 50 (19.0) |
| Other cognitive impairment | 40 (15.2) | Abnormal posture | 46 (17.5) |
| Daytime dysuria | 39 (14.8) | Agitation/aggression | 45 (17.1) |
| Disorientation | 38 (14.4) | Daytime dysuria | 45 (17.1) |
| Rapid eye movement sleep behavior disorder | 36 (13.7) | Hallucinations other than visual hallucinations | 43 (16.3) |
| Fluctuating cognition | 36 (13.7) | Other cognitive impairment | 43 (16.3) |
| Visuospatial dysfunction | 30 (11.4) | Visuospatial dysfunction | 43 (16.3) |
| Apathy | 30 (11.4) | Other psychiatric symptom | 40 (15.2) |
| Anxiety | 28 (10.6) | Delusions | 36 (13.7) |
| Dysosmia | 28 (10.6) | Freezing of gait | 35 (13.3) |
| Hallucinations other than visual hallucinations | 27 (10.3) | Loss of appetite | 35 (13.3) |
| Weight loss | 26 (9.9) | Anxiety | 33 (12.5) |
| Loss of appetite | 25 (9.5) | Nighttime sleep disorder | 33 (12.5) |
| Rest tremor | 25 (9.5) | Weight loss | 33 (12.5) |
| Fall | 24 (9.1) | Action tremor | 29 (11.0) |
| Other psychiatric symptom | 24 (9.1) | Dysosmia | 29 (11.0) |
| Salivation | 24 (9.1) | Dysphagia | 28 (10.6) |
| Orthostatic hypotension | 21 (8.0) | Fall | 27 (10.3) |
| Dysphagia | 18 (6.8) | Orthostatic hypotension | 27 (10.3) |
| Rigidity | 18 (6.8) | Aberrant motor behavior | 26 (9.9) |
| Weight gain | 17 (6.5) | Salivation | 25 (9.5) |
| Delusions | 16 (6.1) | Sudden sleep | 25 (9.5) |
| Restless legs syndrome | 16 (6.1) | Negativism | 23 (8.7) |
| Dizziness | 15 (5.7) | Disturbance of sweating | 21 (8.0) |
| Sudden sleep | 15 (5.7) | Increase in appetite | 21 (8.0) |
| Disturbance of sweating | 14 (5.3) | Weight gain | 20 (7.6) |
| Increase in appetite | 14 (5.3) | Delirium | 16 (6.1) |
| Delirium | 12 (4.6) | Day-night reversal | 15 (5.7) |
| Agitation/aggression | 11 (4.2) | Rest tremor | 15 (5.7) |
| Day-night reversal | 10 (3.8) | Rigidity | 15 (16.3) |
| Negativism | 8 (3.0) | Dizziness | 14 (5.3) |
| Aberrant motor behavior | 7 (2.7) | Unbalanced diet | 12 (4.6) |
| Unbalanced diet | 5 (1.9) | Food refusal | 10 (3.8) |
| Food refusal | 4 (1.5) | Restless legs syndrome | 8 (3.0) |
| Disinhibition | 3 (1.1) | Syncope | 8 (3.0) |
| Syncope | 3 (1.1) | Disinhibition | 7 (2.7) |
| Periodic limb movement disorder | 2 (0.8) | Periodic limb movement disorder | 7 (2.7) |
| Eating non-edible things | 1 (0.4) | Eating non-edible things | 2 (0.8) |
| Invalid answer | 43 (16.3) | Invalid answer | 15 (5.7) |
| Do not know | 31 (11.8) | Do not know | 10 (3.8) |
| Unanswered | 12 (4.6) | Unanswered | 5 (1.9) |
