# Supplementary figures and images for: Treatment needs of dementia with Lewy bodies according to patients, caregivers, and physicians: a cross-sectional, observational, questionnaire-based study in Japan
Source: Alzheimers Res Ther. 2022 Dec 15;14:188. doi: 10.1186/s13195-022-01130-4 (PMC9751509; doi:10.1186/s13195-022-01130-4)

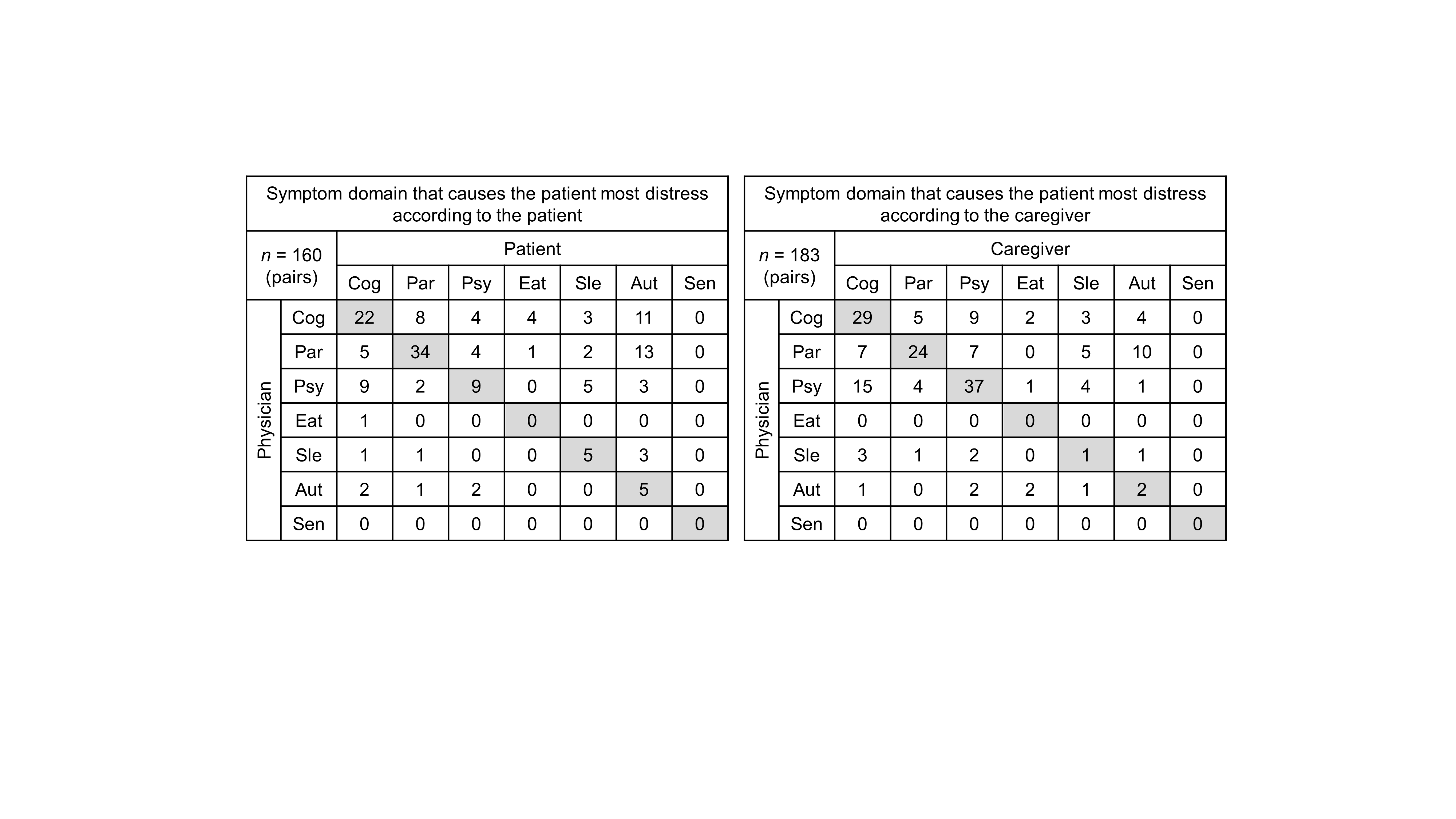

Supplement: Supplementary file 8 — Additional file 8: Supplementary Fig. 1. Number of answers selected by patients and physicians and by caregivers and physicians for “symptom domain that causes the patient most distress according to the patient/caregiver”. The gray cells indicate the symptom domains that are consistent between the two groups. Abbreviations: Aut autonomic dysfunction, Cog cognitive impairment, Eat eating behavior-related problems, Par parkinsonism, Psy psychiatric symptoms, Sen sensory disorders, Sle sleep-related disorders. [file 13195_2022_1130_MOESM8_ESM.tif]
