# Supplementary material for: Treatment needs of dementia with Lewy bodies according to patients, caregivers, and physicians: a cross-sectional, observational, questionnaire-based study in Japan
Source: Alzheimers Res Ther. 2022 Dec 15;14:188. doi: 10.1186/s13195-022-01130-4 (PMC9751509; doi:10.1186/s13195-022-01130-4)
Supplement: Supplementary file 9 — Additional file 9: Supplementary Table 3. Symptom domain causing patients most distress: matching status and regression analysis for patient–physician discordance. [file 13195_2022_1130_MOESM9_ESM.docx]

**Supplementary Table 3** **Symptom domain causing patients most distress: matching status and regression analysis for patient–physician discordance**

| **Between patient and physician** | **Symptom domain that causes the patient most distress** | | **Univariate LR analysis** | | |
| --- | --- | --- | --- | --- | --- |
|  | **Concordance**  **(*n* = 75)** | **Discordance**  **(*n* = 85)** | **OR** | **95% CI** | ***p* value** |
| **Patient-side factors** | | | | | |
| Patient’s sex | | | | | |
| Male | 39 (52.0) | 36 (42.4) | 1.000 | ref |  |
| Female | 36 (48.0) | 49 (57.6) | 1.475 | 0.789–2.754 | 0.223 |
| Duration of DLB (m) | | | | | |
| <24.0 | 32 (42.7) | 48 (57.8) | 1.000 | ref |  |
| ≥24.0 | 43 (57.3) | 35 (42.2) | 0.543 | 0.288–1.021 | 0.058 |
| Duration of education (y) | | | | | |
| <12.0 | 19 (27.5) | 20 (26.0) | 1.000 | ref |  |
| ≥12.0 | 50 (72.5) | 57 (74.0) | 1.083 | 0.520–2.256 | 0.831 |
| Patient’s knowledge of DLB | - |  |  |  |  |
| Knows a lot about it. | 7 (9.3) | 4 (4.7) | 1.000 | ref |  |
| Neither yes nor no, does not know very much | 68 (90.7) | 81 (95.3) | 2.085 | 0.585–7.423 | 0.257 |
| Frequency of hospital or clinic visits | | | | | |
| Once every 2 to 3 weeks, once every month | 26 (36.1) | 38 (45.2) | 1.000 | ref |  |
| Once every 2 months, once every 3 months | 44 (61.1) | 46 (54.8) | 0.715 | 0.374–1.367 | 0.311 |
| Once every 4 months or more | 2 (2.8) | 0 (0.0) | n.c. |  |  |
| Facility use (long-term care, outpatient rehabilitation, multifunctional home care) | | | | | |
| None | 37 (49.3) | 40 (47.1) | 1.000 | ref |  |
| Yes | 38 (50.7) | 45 (52.9) | 1.095 | 0.588–2.039 | 0.774 |
| Patient’s understanding from physician’s point of view | | | | | |
| Excellent, good | 57 (76.0) | 55 (64.7) | 1.000 | ref |  |
| Normal | 9 (12.0) | 19 (22.4) | 2.188 | 0.912–5.250 | 0.080 |
| Poor, no understanding | 9 (12.0) | 11 (12.9) | 1.267 | 0.487–3.294 | 0.628 |
| Duration from presenting the initial symptom domain | | | | | |
| Less than 1 year | 10 (13.5) | 15 (17.6) | 1.000 | ref |  |
| Between 1 and 3 years | 17 (23.0) | 22 (25.9) | 0.863 | 0.311–2.393 | 0.777 |
| Between 3 and 5 years | 15 (20.3) | 23 (27.1) | 1.022 | 0.365–2.867 | 0.967 |
| More than 5 years | 30 (40.5) | 21 (24.7) | 0.467 | 0.176–1.238 | 0.126 |
| Unknown | 2 (2.7) | 4 (4.7) | 1.333 | 0.204–8.708 | 0.764 |
| MMSE-J | | | | | |
| <22.0 | 46 (61.3) | 50 (58.8) | 1.000 | ref |  |
| ≥22.0 | 29 (38.7) | 35 (41.2) | 1.110 | 0.589–2.094 | 0.746 |
| NPI-10 | | | | | |
| <11.0 | 41 (55.4) | 42 (49.4) | 1.000 | ref |  |
| ≥11.0 | 33 (44.6) | 43 (50.6) | 1.272 | 0.681–2.377 | 0.451 |
| NPI subitem “Nighttime behaviors” | | | | | |
| <1.0 | 49 (66.2) | 55 (64.7) | 1.000 | ref |  |
| ≥1.0 | 25 (33.8) | 30 (35.3) | 1.069 | 0.555–2.060 | 0.842 |
| NPI subitem “Appetite” | | | | | |
| <1.0 | 51 (68.9) | 63 (74.1) | 1.000 | ref |  |
| ≥1.0 | 23 (31.1) | 22 (25.9) | 0.774 | 0.388–1.546 | 0.468 |
| Autonomic dysfunction |  |  |  |  |  |
| None | 42 (56.0) | 48 (56.5) | 1.000 | ref |  |
| Yes | 33 (44.0) | 37 (43.5) | 0.981 | 0.525–1.834 | 0.952 |
| Sensory disorders |  |  |  |  |  |
| None | 66 (88.0) | 70 (90.6) | 1.000 | ref |  |
| Yes | 9 (12.0) | 8 (9.4) | 0.762 | 0.278–2.087 | 0.597 |
| CFI |  |  |  |  |  |
| <1.0 | 40 (53.3) | 33 (38.8) | 1.000 | ref |  |
| ≥1.0 | 35 (46.7) | 52 (61.2) | 1.801 | 0.960–3.379 | 0.067 |
| Pharmacotherapy for psychiatric symptoms | | | | | |
| None | 46 (61.3) | 40 (47.1) | 1.000 | ref |  |
| Yes | 29 (38.7) | 45 (52.9) | 1.784 | 0.950–3.352 | 0.072 |
| Pharmacotherapy for sleep-related disorders | | | | | |
| None | 49 (65.3) | 50 (58.8) | 1.000 | ref |  |
| Yes | 26 (34.7) | 35 (41.2) | 1.319 | 0.694–2.508 | 0.398 |
| Pharmacotherapy for autonomic dysfunction | | | | | |
| None | 60 (80.0) | 65 (76.5) | 1.000 | ref |  |
| Yes | 15 (20.0) | 20 (23.5) | 1.231 | 0.578–2.621 | 0.590 |
| Physician listens to what patient say | | | | | |
| Very well, well | 53 (70.7) | 64 (75.3) | 1.000 | ref |  |
| Normal | 15 (20.0) | 18 (21.2) | 0.994 | 0.457–2.159 | 0.987 |
| Not much, not at all, do not know | 7 (9.3) | 3 (3.5) | 0.355 | 0.087–1.440 | 0.147 |
| Someone other than the physician with whom the patient can talk at the hospital/clinic | | | | | |
| Yes | 24 (32.0) | 32 (37.6) | 1.000 | ref |  |
| None, do not know | 51 (68.0) | 53 (62.4) | 0.779 | 0.405–1.499 | 0.455 |
| Whether the patient told the physician about patient’s most inconvenient symptom | | | | | |
| Yes | 51 (71.8) | 49 (59.8) | 1.000 | ref |  |
| No | 12 (16.9) | 25 (30.5) | 2.168 | 0.982–4.788 | 0.055 |
| Unknown | 8 (11.3) | 8 (9.8) | 1.041 | 0.362–2.991 | 0.941 |
| From the physician’s perspective, whether there is someone at the hospital/clinic other than the physician with whom the patient can talk | | | | | |
| Yes | 44 (58.7) | 44 (51.8) | 1.000 | ref |  |
| No | 31 (41.3) | 41 (48.2) | 1.323 | 0.707–2.474 | 0.382 |
| Appropriate frequency of hospital or clinic visits of patient | | | | | |
| Once every 2 to 3 weeks, once every month | 28 (37.3) | 35 (41.2) | 1.000 | ref |  |
| Once every 2 months, once every 3 months | 47 (62.7) | 49 (57.6) | 0.834 | 0.441–1.579 | 0.577 |
| Once every 4 months or more | 0 (0.0) | 1 (1.2) | n.c. |  |  |
| **Caregiver-side factors** | | | | | |
| Caregiver’s age (y) | | | | | |
| <65.0 | 40 (53.3) | 54 (63.5) | 1.000 | ref |  |
| ≥65.0 | 35 (46.7) | 31 (36.5) | 0.656 | 0.348–1.236 | 0.192 |
| Caregiver’s sex | | | | | |
| Male | 15 (20.0) | 23 (27.1) | 1.000 | ref |  |
| Female | 60 (80.0) | 62 (72.9) | 0.674 | 0.321–1.414 | 0.297 |
| Caregiver’s knowledge of DLB | | | | | |
| Knows a lot about it. | 16 (21.9) | 22 (26.2) | 1.000 | ref |  |
| Nether yes or no, does not know very much | 57 (78.1) | 62 (73.8) | 0.791 | 0.378–1.654 | 0.533 |
| Job | | | | | |
| Yes | 31 (41.3) | 45 (53.6) | 1.000 | ref |  |
| No | 44 (58.7) | 39 (46.4) | 0.611 | 0.326–1.145 | 0.124 |
| Assistant caregiver |  |  |  |  |  |
| Yes | 23 (30.7) | 29 (34.1) | 1.000 | ref |  |
| No | 52 (69.3) | 56 (65.9) | 0.854 | 0.439–1.660 | 0.642 |
| Living with the patient | | | | | |
| Yes | 63 (84.0) | 63 (74.1) | 1.000 | ref |  |
| No | 12 (16.0) | 22 (25.9) | 1.833 | 0.836–4.021 | 0.130 |
| Frequency of hospital or clinic visits desired by caregiver | | | | | |
| Once every 2 to 3 weeks, once every month | 26 (35.1) | 36 (43.4) | 1.000 | ref |  |
| Once every 2 months, once every 3 months | 47 (63.5) | 47 (56.6) | 0.722 | 0.378–1.379 | 0.324 |
| Once every 4 months or more | 1 (1.4) | 0 (0.0) | n.c. |  |  |
| Caregiver’s understanding from physician’s point of view | | | | | |
| Excellent, good | 71 (94.7) | 77 (90.6) | 1.000 | ref |  |
| Normal | 4 (5.3) | 5 (5.9) | 1.153 | 0.298–4.463 | 0.837 |
| Poor, no understanding | 0 (0.0) | 3 (3.5) | n.c. |  |  |
| J-ZBI_8 | | | | | |
| <7.0 | 40 (53.3) | 34 (40.0) | 1.000 | ref |  |
| ≥7.0 | 35 (46.7) | 51 (60.0) | 1.714 | 0.915–3.212 | 0.092 |
| Patient’s physician listens to what the caregiver says | | | | | |
| Very well, well | 64 (85.3) | 75 (88.2) | 1.000 | ref |  |
| Normal | 9 (12.0) | 8 (9.4) | 0.759 | 0.277–2.081 | 0.591 |
| Not much, not at all, do not know | 2 (2.7) | 2 (2.4) | 0.853 | 0.117–6.231 | 0.876 |
| Someone other than physician with whom caregiver can talk | | | | | |
| Yes | 31 (41.3) | 36 (42.4) | 1.000 | ref |  |
| None, do not know | 44 (58.7) | 49 (57.6) | 0.959 | 0.511–1.800 | 0.896 |
| **Physician-side factors** | | | | | |
| Physician’s age (y) | | | | | |
| <50.0 | 30 (40.0) | 44 (51.8) | 1.000 | ref |  |
| ≥50.0 | 45 (60.0) | 41 (48.2) | 0.621 | 0.332–1.164 | 0.137 |
| Physician’s sex |  |  |  |  |  |
| Male | 63 (84.0) | 74 (87.1) | 1.000 | ref |  |
| Female | 12 (16.0) | 11 (12.9) | 0.780 | 0.322–1.890 | 0.780 |
| Number of DLB patients treated to date | | | | | |
| Between 10 and 99 | 18 (24.0) | 19 (22.4) | 1.000 | ref |  |
| ≤100 | 57 (76.0) | 66 (77.6) | 1.097 | 0.526–2.289 | 0.805 |
| Symptom domain prioritized for treatment  (If patient exhibits hallucinations, delusions, and parkinsonism of same level impairment) | | | | | |
| Psychiatric symptoms | 56 (74.7) | 62 (72.9) | 1.000 | ref |  |
| Parkinsonism | 19 (25.3) | 23 (27.1) | 1.093 | 0.539–2.217 | 0.805 |
| Clinical departments | | | | | |
| Department of psychiatry | 33 (44.0) | 44 (51.8) | 1.000 | ref |  |
| Other than psychiatry | 42 (56.0) | 41 (48.2) | 0.732 | 0.392–1.366 | 0.327 |
| Institute |  |  |  |  |  |
| University hospital | 38 (50.7) | 38 (44.7) | 1.000 | ref |  |
| Non-university hospital | 14 (18.7) | 16 (18.8) | 1.143 | 0.490–2.665 | 0.757 |
| Clinic | 23 (30.7) | 31 (36.5) | 1.348 | 0.668–2.720 | 0.405 |
| Refer to the Guidelines for Dementia by the Japan Society for Dementia Research | | | | | |
| Yes | 73 (97.3) | 83 (97.6) | 1.000 | ref |  |
| No | 2 (2.7) | 2 (2.4) | 0.880 | 0.121–6.402 | 0.899 |
| Off-label prescribing of medications |  |  |  |  |  |
| Often | 28 (37.3) | 38 (44.7) | 1.000 | ref |  |
| Sometimes | 47 (62.7) | 47 (55.3) | 0.737 | 0.391–1.389 | 0.345 |
| Duration of the patient treatment | | | | | |
| Less than half a year | 9 (12.0) | 13 (15.3) | 1.000 | ref |  |
| Between half a year and less than 1 year | 12 (16.0) | 20 (23.5) | 1.154 | 0.380–3.505 | 0.801 |
| ≥1 year | 54 (72.0) | 52 (61.2) | 0.667 | 0.263–1.692 | 0.393 |

Only no-significant items (excluding significant items) in univariate analysis were shown.

*Abbreviations*: *CI* confidence interval, *CFI* Cognitive Fluctuation Inventory, *DLB* dementia with Lewy bodies, *J-ZBI_8* shortened Japanese version of the Zarit Caregiver Burden Interview, *LR* logistic regression, *MMSE-J* Japanese version of the Mini-Mental State Examination, *n.c.* not calculated, *n.e.* not evaluable, *NPI-10* Japanese version of the Neuropsychiatric Inventory-10, *OR* odds ratio, *ref* reference
