# Supplementary material for: Treatment needs of dementia with Lewy bodies according to patients, caregivers, and physicians: a cross-sectional, observational, questionnaire-based study in Japan
Source: Alzheimers Res Ther. 2022 Dec 15;14:188. doi: 10.1186/s13195-022-01130-4 (PMC9751509; doi:10.1186/s13195-022-01130-4)
Supplement: Supplementary file 10 — Additional file 10: Supplementary Table 4. Symptom domain causing caregivers most distress: matching status and regression analysis for caregiver–physician discordance. [file 13195_2022_1130_MOESM10_ESM.docx]

**Supplementary Table 4** **Symptom domain causing caregivers most distress: matching status and regression analysis for caregiver–physician discordance**

| **Between caregiver and physician** | **Symptom domain that causes the caregiver most distress** | | **Univariate LR analysis** | | | | | | |
| --- | --- | --- | --- | --- | --- | --- | --- | --- | --- |
|  | **Concordance**  **(*n* = 93)** | **Discordance**  **(*n* = 90)** | **OR** | **95% CI** | | | | ***p* value** | |
| **Patient-side factors** | | | | | | | | | |
| Patient’s age (y) | | | | | | | | | |
| <80.0 | 47 (50.5) | 41 (45.6) | 1.000 | | ref | | | |  |
| ≥80.0 | 46 (49.5) | 49 (54.4) | 1.221 | | 0.683–2.183 | | | | 0.500 |
| Patient’s sex | | | | | | | | | |
| Male | 49 (52.7) | 36 (40.0) | 1.000 | | ref | | | |  |
| Female | 44 (47.3) | 54 (60.0) | 1.670 | | 0.930–3.002 | | | | 0.086 |
| Duration of DLB (m) | | | | | | | | | |
| <24.0 | 47 (51.1) | 41 (46.1) | 1.000 | | ref | | | |  |
| ≥24.0 | 45 (48.9) | 48 (53.9) | 1.223 | | 0.682–2.192 | | | | 0.500 |
| Duration of education (y) | | | | | | | | | |
| <12.0 | 21 (25.6) | 27 (31.4) | 1.000 | | ref | | | |  |
| ≥12.0 | 61 (74.4) | 59 (68.6) | 0.752 | | 0.384–1.475 | | | | 0.407 |
| Number of persons living with the patient | | | | | | | | | |
| Alone | 8 (8.6) | 12 (13.3) | 1.000 | | ref | | | |  |
| Two | 40 (43.0) | 36 (40.0) | 0.600 | | 0.220–1.633 | | | | 0.317 |
| Three or more | 45 (48.4) | 42 (46.7) | 0.622 | | 0.232–1.672 | | | | 0.347 |
| Patient’s knowledge of DLB | | | | | | | | | |
| Knows a lot about it | 6 (6.5) | 8 (9.0) | 1.000 | | ref | | | |  |
| Neither yes nor no, does not know very much | 87 (93.5) | 81 (91.0) | 0.698 | | 0.232–2.100 | | | | 0.523 |
| Frequency of hospital or clinic visits | | | | | | | | | |
| Once every 2 to 3 weeks, once every month | 40 (43.5) | 39 (44.3) | 1.000 | | ref | | | |  |
| Once every 2 months, once every 3 months | 51 (55.4) | 48 (54.5) | 0.965 | | 0.534–1.744 | | | | 0.907 |
| Once every 4 months or more | 1 (1.1) | 1 (1.1) | 1.026 | | 0.062–16.979 | | | | 0.986 |
| Facility use (long-term care, outpatient rehabilitation, multifunctional home care) | | | | | | | | | |
| None | 44 (47.3) | 32 (35.6) | 1.000 | | ref | | | |  |
| Yes | 49 (52.7) | 58 (64.4) | 1.628 | | 0.899–2.946 | | | | 0.108 |
| Patient’s understanding from physician’s point of view | | | | | | | | | |
| Excellent, good | 57 (61.3) | 60 (66.7) | 1.000 | | ref | | | |  |
| Normal | 15 (16.1) | 16 (17.8) | 1.013 | | 0.459–2.238 | | | | 0.974 |
| Poor, no understanding | 21 (22.6) | 14 (15.6) | 0.633 | | 0.294–1.364 | | | | 0.243 |
| Patient’s initial symptom domain | | | | | | | | | |
| Cognitive impairment | 32 (34.8) | 32 (36.0) | 1.231 | | 0.510–2.969 | | | | 0.644 |
| Parkinsonism | 16 (17.4) | 13 (14.6) | 1.000 | | ref | | | |  |
| Psychiatric symptoms | 25 (27.2) | 23 (25.8) | 1.132 | | 0.449–2.857 | | | | 0.792 |
| Eating behavior-related problems | 1 (1.1) | 0 (0.0) | n.c. | |  | | | |  |
| Sleep-related disorders | 12 (13.0) | 15 (16.9) | 1.538 | | 0.536–4.416 | | | | 0.423 |
| Autonomic dysfunction | 2 (2.2) | 3 (3.4) | 1.846 | | 0.267–12.758 | | | | 0.534 |
| Sensory disorders | 4 (4.3) | 3 (3.4) | 0.923 | | 0.174–4.885 | | | | 0.925 |
| Duration from presenting the initial symptom domain | | | | | | | | | |
| Less than 1 year | 10 (10.9) | 9 (10.1) | 1.000 | | ref | | | |  |
| Between 1 and 3 years | 25 (27.2) | 21 (23.6) | 0.933 | | 0.320–2.724 | | | | 0.900 |
| Between 3 and 5 years | 19 (20.7) | 29 (32.6) | 1.696 | | 0.582–4.946 | | | | 0.333 |
| More than 5 years | 35 (38.0) | 28 (31.5) | 0.889 | | 0.318–2.486 | | | | 0.822 |
| Unknown | 3 (3.3) | 2 (2.2) | 0.741 | | 0.100–5.490 | | | | 0.769 |
| MMSE-J | | | | | | | | | |
| <18.0 | 44 (47.3) | 51 (56.7) | 1.000 | | ref | | | |  |
| ≥18.0 | 49 (52.7) | 39 (43.3) | 0.687 | | 0.383–1.230 | | | | 0.206 |
| MDS-UPDRS Part III total score | | | | | | | | | |
| ≥22.0 | 44 (47.3) | 51 (56.7) | 1.000 | | ref | | | |  |
| <22.0 | 49 (52.7) | 39 (43.3) | 0.687 | | 0.383–1.230 | | | | 0.206 |
| NPI-10 | | | | | | | | | |
| <11.0 | 44 (48.4) | 48 (53.3) | 1.000 | | ref | | | |  |
| ≥11.0 | 47 (51.6) | 42 (46.7) | 0.819 | | 0.457–1.468 | | | | 0.503 |
| NPI subitem “Nighttime behaviors” | | | | | | | | | |
| <1.0 | 62 (68.1) | 56 (62.2) | 1.000 | | ref | | | |  |
| ≥1.0 | 29 (31.9) | 34 (37.8) | 1.298 | | 0.703–2.397 | | | | 0.404 |
| NPI subitem “Appetite” | | | | | | | | | |
| <1.0 | 61 (67.0) | 66 (73.3) | 1.000 | | ref | | | |  |
| ≥1.0 | 30 (33.0) | 24 (26.7) | 0.739 | | 0.390–1.402 | | | | 0.355 |
| Sensory disorders | | | | | | | | | |
| None | 87 (93.5) | 78 (86.7) | 1.000 | | | ref | | |  |
| Yes | 6 (6.5) | 12 (13.3) | 2.231 | | | 0.799–6.227 | | | 0.126 |
| MDS-UPDRS Part II total score | | | | | | | | | |
| <9.0 | 42 (45.2) | 44 (49.4) | 1.000 | | | ref | | |  |
| ≥9.0 | 51 (54.8) | 45 (50.6) | 0.842 | | | 0.470–1.508 | | | 0.564 |
| CFI | | | | | | | | | |
| <1.0 | 41 (44.1) | 36 (40.0) | 1.000 | | | ref | | |  |
| ≥1.0 | 52 (55.9) | 54 (60.0) | 1.183 | | | 0.657–2.129 | | | 0.576 |
| Pharmacotherapy for cognitive impairment | | | | | | | | | |
| No | 23 (24.7) | 16 (17.8) | 1.000 | | | ref | | |  |
| Yes | 70 (75.3) | 74 (82.2) | 1.520 | | | 0.742–3.112 | | | 0.253 |
| Pharmacotherapy for parkinsonism | | | | | | | | | |
| No | 57 (61.3) | 52 (57.8) | 1.000 | | | ref | | |  |
| Yes | 36 (38.7) | 38 (42.2) | 1.157 | | | 0.641–2.089 | | | 0.628 |
| Pharmacotherapy for psychiatric symptoms | | | | | | | | | |
| No | 47 (50.5) | 49 (54.4) | 1.000 | | | ref | | |  |
| Yes | 46 (49.5) | 41 (45.6) | 0.855 | | | 0.478–1.528 | | | 0.597 |
| Pharmacotherapy for sleep-related disorders | | | | | | | | | |
| No | 53 (57.0) | 56 (62.2) | 1.000 | | | ref | | |  |
| Yes | 40 (43.0) | 34 (37.8) | 0.804 | | | 0.445–1.454 | | | 0.471 |
| Pharmacotherapy for autonomic dysfunction | | | | | | | | | |
| No | 78 (83.9) | 71 (78.9) | 1.000 | | | ref | | |  |
| Yes | 15 (16.1) | 19 (21.1) | 1.392 | | | 0.658–2.944 | | | 0.388 |
| From the physician’s perspective, whether there is someone at the hospital/clinic other than the physician with whom the patient can talk | | | | | | | | | |
| Yes | 50 (53.8) | 53 (58.9) | 1.000 | | | ref | | |  |
| None | 43 (46.2) | 37 (41.1) | 0.812 | | | 0.452–1.457 | | | 0.485 |
| Appropriate frequency of hospital or clinic visits of patient | | | | | | | | | |
| Once every 2 to 3 weeks, once every month | 43 (46.2) | 35 (38.9) | 1.000 | | | ref | | |  |
| Once every 2 months, once every 3 months | 50 (53.8) | 55 (61.1) | 1.351 | | | 0.751–2.433 | | | 0.315 |
| Once every 4 months or more | 0 (0.0) | 0 (0.0) | n.c. | | |  | | |  |
| **Caregiver-side factors** | | | | | | | | | |
| Caregiver’s age (y) | | | | | | | | | |
| <65.0 | 52 (55.9) | 59 (65.6) | 1.000 | | | ref | | |  |
| ≥65.0 | 41 (44.1) | 31 (34.4) | 0.666 | | | 0.367–1.211 | | | 0.183 |
| Caregiver’s sex | | | | | | | | | |
| Male | 27 (29.0) | 18 (20.0) | 1.000 | | | ref | | |  |
| Female | 66 (71.0) | 72 (80.0) | 1.636 | | | 0.826–3.241 | | | 0.158 |
| Caregiver’s knowledge of DLB | | | | | | | | | |
| Knows a lot about it. | 24 (25.8) | 28 (31.5) | 1.000 | | | ref | | |  |
| Nether yes or no, does not know very much | 69 (74.2) | 61 (68.5) | 0.758 | | | 0.398–1.444 | | | 0.399 |
| Job | | | | | | | | | |
| Yes | 41 (44.1) | 48 (53.3) | 1.000 | | | ref | | |  |
| None | 52 (55.9) | 42 (46.7) | 0.690 | | | 0.385–1.235 | | | 0.211 |
| Assistant caregiver |  |  |  | | |  | | |  |
| Yes | 38 (40.9) | 29 (32.2) | 1.000 | | | ref | | |  |
| None | 55 (59.1) | 61 (67.8) | 1.453 | | | 0.793–2.662 | | | 0.226 |
| Relationship with the patient from patient's perspective | | | | | | | | | |
| Spouse | 47 (50.5) | 33 (36.7) | 1.000 | | | ref | | |  |
| Non-spouse | 46 (49.5) | 57 (63.3) | 1.765 | | | 0.978–3.186 | | | 0.059 |
| Living with the patient | | | | | | | | | |
| Yes | 71 (76.3) | 66 (73.3) | 1.000 | | | ref | | |  |
| No | 22 (23.7) | 24 (26.7) | 1.174 | | | 0.601–2.290 | | | 0.639 |
| Time spent with the patient (h/day) | | | | | | | | | |
| <16.0 | 49 (53.3) | 49 (55.7) | 1.000 | | | ref | | |  |
| ≥16.0 | 43 (46.7) | 39 (44.3) | 0.907 | | | 0.504–1.631 | | | 0.744 |
| Frequency of hospital or clinic visits desired by caregiver | | | | | | | | | |
| Once every 2 to 3 weeks, once every month | 36 (39.6) | 39 (43.8) | 1.000 | | | ref | | |  |
| Once every 2 months, once every 3 months | 54 (59.3) | 50 (56.2) | 0.855 | | | 0.472–1.548 | | | 0.605 |
| Once every 4 months or more | 1 (1.1) | 0 (0.0) | n.c. | | |  | | |  |
| Caregiver’s understanding from physician’s point of view | | | | | | | | | |
| Excellent, good | 82 (88.2) | 83 (92.2) | 1.000 | | | | ref | |  |
| Normal | 8 (8.6) | 5 (5.6) | 0.617 | | | | 0.194–1.966 | | 0.415 |
| Poor, no understanding | 3 (3.2) | 2 (2.2) | 0.659 | | | | 0.107–4.045 | | 0.652 |
| J-ZBI_8 | | | | | | | | | |
| <7.0 | 43 (46.2) | 42 (46.7) | 1.000 | | | | ref | |  |
| ≥7.0 | 50 (53.8) | 48 (53.3) | 0.983 | | | | 0.550–1.757 | | 0.953 |
| Patient’s physician listens to what the caregiver says | | | | | | | | | |
| Very well, well | 80 (86.0) | 79 (87.8) | 1.000 | | | | ref | |  |
| Normal | 10 (10.8) | 8 (8.9) | 0.810 | | | | 0.304–2.159 | | 0.674 |
| Not much, not at all, do not know | 3 (3.2) | 3 (3.3) | 1.013 | | | | 0.198–5.170 | | 0.988 |
| Someone other than physician with whom caregiver can talk | | | | | | | | | |
| Yes | 35 (37.6) | 37 (41.6) | 1.000 | | | | ref | |  |
| None, do not know | 58 (62.4) | 52 (58.4) | 0.848 | | | | 0.468–1.537 | | 0.587 |
| Whether caregiver told the physician about caregiver’s most inconvenient symptoms | | | | | | | | | |
| No | 72 (80.9) | 71 (81.6) | 1.000 | | | | ref | |  |
| Yes | 13 (14.6) | 13 (14.9) | 1.014 | | | | 0.440–2.339 | | 0.974 |
| Unknown | 4 (4.5) | 3 (3.4) | 0.761 | | | | 0.164–3.521 | | 0.726 |
| **Physician-side factors** | | | | | | | | | |
| Physician’s age (y) | | | | | | | | | |
| <50.0 | 51 (54.8) | 45 (50.0) | 1.000 | | | | ref | |  |
| ≥50.0 | 42 (45.2) | 45 (50.0) | 1.214 | | | | 0.679–2.171 | | 0.512 |
| Physician’s sex |  |  |  | | | |  | |  |
| Male | 78 (83.9) | 71 (78.9) | 1.000 | | | | ref | |  |
| Female | 15 (16.1) | 19 (21.1) | 1.392 | | | | 0.658–2.944 | | 0.388 |
| Number of DLB patients treated to date | | | | | | | | | |
| Between 10 and 99 | 20 (21.5) | 21 (23.3) | 1.000 | | | | ref | |  |
| ≤100 | 73 (78.5) | 69 (76.7) | 0.900 | | | | 0.449–1.804 | | 0.767 |
| Symptom domain prioritized for treatment  (If patient exhibits hallucinations, delusions, and parkinsonism of same level impairment) | | | | | | | | | |
| Psychiatric symptoms | 66 (71.0) | 68 (75.6) | 1.000 | | | | ref | |  |
| Parkinsonism | 27 (29.0) | 22 (24.4) | 0.900 | | | | 0.410–1.525 | | 0.484 |
| Clinical departments | | | | | | | | | |
| Department of psychiatry | 46 (49.5) | 38 (42.2) | 1.000 | | | | ref | |  |
| Other than psychiatry | 47 (50.5) | 52 (57.8) | 1.339 | | | | 0.747–2.400 | | 0.326 |
| Refer to the Guidelines for Dementia by the Japan Society for Dementia Research | | | | | | | | | |
| No | 92 (98.9) | 88 (97.8) | 1.000 | | | | ref | |  |
| Yes | 1 (1.1) | 2 (2.2) | 2.091 | | | | 0.186–23.471 | | 0.550 |
| Off-label prescribing of medications | | | | | | | | | |
| Often | 38 (40.9) | 39 (43.3) | 1.000 | | | | ref | |  |
| Sometimes | 55 (59.1) | 51 (56.7) | 0.903 | | | | 0.502–1.625 | | 0.735 |
| Duration of the patient’s treatment | | | | | | | | | |
| Less than half a year | 12 (12.9) | 11 (12.2) | 1.000 | | | | ref | |  |
| Between half a year and less than 1 year | 18 (19.4) | 16 (17.8) | 0.970 | | | | 0.336–2.798 | | 0.955 |
| ≥1 year | 63 (67.7) | 63 (70.0) | 1.091 | | | | 0.448–2.655 | | 0.848 |

Only no-significant items (excluding significant items) in univariate analysis were shown.

*Abbreviations*: *CI* confidence interval, *CFI* Cognitive Fluctuation Inventory, *DLB* dementia with Lewy bodies, *J-ZBI_8* shortened Japanese version of the Zarit Caregiver Burden Interview, *LR* logistic regression, *MDS-UPDRS* Movement Disorder Society-Unified Parkinson’s Disease Rating Scale, *MMSE-J* Japanese version of the Mini-Mental State Examination, *n.c.* not calculated, *n.e.* not evaluable, *NPI-10* Japanese version of the Neuropsychiatric Inventory-10, *OR* odds ratio, *ref* reference
