# Supplementary material for: Treatment needs of dementia with Lewy bodies according to patients, caregivers, and physicians: a cross-sectional, observational, questionnaire-based study in Japan
Source: Alzheimers Res Ther. 2022 Dec 15;14:188. doi: 10.1186/s13195-022-01130-4 (PMC9751509; doi:10.1186/s13195-022-01130-4)
Supplement: Supplementary file 11 — Additional file 11: Supplementary information about coinvestigators. List of coinvestigators. [file 13195_2022_1130_MOESM11_ESM.docx]

**Supplementary information about coinvestigators**

Kenji Yoshiyama, Hideki Kanemoto, and Takashi Suehiro (Department of Psychiatry, Osaka University Graduate School of Medicine, Suita, Osaka, Japan); Etsuro Mori (Department of Behavioral Neurology and Neuropsychiatry, Osaka University United Graduate School of Child Development, Suita, Osaka, Japan); Shunichiro Shinagawa and Emi Takasaki (Department of Psychiatry, The Jikei University School of Medicine, Minato-ku, Tokyo, Japan); Hirotake Uchikado (Memory Care Clinic Shonan, Hiratsuka, Kanagawa, Japan); Shinobu Kawakatsu, Kayo Saigo, and Tetsuya Shiga (Department of Neuropsychiatry, Aizu Medical Center, Fukushima Medical University, Aizuwakamatsu, Fukushima, Japan); Naoko Tsunoda and Naohisa Otsuka (Mitsugumachi Clinic, Kita-ku, Kumamoto, Japan); Seigo Nakano (Higashi Zaitaku Clinic, Higashi-ku, Sapporo, Japan); Kazue Shigenobu, Norio Taniguchi, Yoshihiro Masaki, Yuuki Toi, and Kensaku Shima (Department of Psychiatry, Asakayama General Hospital, Sakai, Osaka, Japan); Shinji Matsunaga (Aioiyama Honobono Memory Clinic, Midori-ku, Nagoya, Japan); Aoi Yoshiiwa (Department of General Medicine, Oita University, Faculty of Medicine, Yufu, Oita, Japan); Shinji Higashi and Yoshihiro Uchida (Department of Psychiatry, Ibaraki Medical Center, Tokyo Medical University, Ami-machi, Ibaraki, Japan); Fumiyoshi Morikawa, Jyuichiro Naoe, and Kazuki Tabata (Asahikawa Keisenkai Hospital, Asahikawa, Hokkaido, Japan); Tokiji Hanihara, Shin Inuzuka, and Yoko Nakano (Nagano Prefectural Mental Wellness Center Komagane, Komagane, Nagano, Japan); Masafuchi Ryo (Department of Neurology, Tokai University Tokyo Hospital, Shibuya-ku, Tokyo, Japan); Naoto Kobayashi (Azuma Street Clinic, Fukushima, Japan); Kou Oka (Mishima Oka Clinic, Mishima, Japan); Kenji Wada (Department of Dementia Research, Kawasaki Medical School General Medical Center, Kita-ku, Okayama, Japan); Kazuko Hasegawa, Aya Kawanami, Naohiko Togashi, Masanobu Miyashita, Yuichi Miyagi, and Sho Tokuda (Neurology, National Hospital Organization, Sagamihara National Hospital, Sagamihara, Kanagawa, Japan); Tsuneo Tsubaki (Tsubaki Clinic, Kamigyou-ku, Kyoto, Japan); Yasuhiko Baba, Shohei Nomoto, Seiya Takahashi, and Owan Yoshiyuki (Department of Neurology, Showa University Fujigaoka Hospital, Yokohama, Kanagawa, Japan); Tadasu Matsuoka (Matsuoka Hospital, Oyabe, Toyama, Japan); Ryuji Sakakibara, Fuyuki Tateno, and Tosuke Aiba (Department of Neurology, Internal Medicine, Sakura Medical Center, Toho University, Sakura, Chiba, Japan); Seigo Shiroma (Shiroma Clinic, Urasoe, Okinawa, Japan); Hajime Baba, Rie Inami, Hitoshi Maeshima, Naoto Yoshinari, and Tomoyuki Sugita (Department of Psychiatry, Juntendo University Koshigaya Hospital, Koshigaya, Saitama, Japan); Naoto Kamimura, Hiroaki Kazui, Tetsuo Kashibayashi, Ryoko Fujito, and Masanori Akamatsu (Department of Neuropsychiatry, Kochi Medical School, Kochi University, Nankoku, Kochi, Japan); Satoshi Kawaguchi (Shimabara Cocorono Memory Clinic, Shimabara, Nagasaki, Japan); Ryota Kobayashi, Hiroshi, Hayashi, Daichi Morioka, and Koichi Otani (Department of Psychiatry, Yamagata University School of Medicine, Yamagata, Japan); Toru Baba (Department of Neurology, National Hospital Organization Sendai-Nishitaga Hospital, Sendai, Miyagi, Japan); Niizato Kazuhiro, Oshima Kenichi, Nao Kawakami, and Kentaro Umeda (Department of Psychiatry, Tokyo Metropolitan Matsuzawa Hospital, Setagaya-ku, Tokyo, Japan); Tae Watanabe and Tsuyoshi Kawamura (Oyabe Ohka Hospital, Oyabe, Toyama, Japan); Nobutaka Yamakawa (Iseyamakawa Clinic, Ise, Mie, Japan); Satoshi Masuzugawa (Masuzugawa Neurology Clinic, Suzuka, Mie, Japan); Kazuki Honda, Ryuji Fukuhara, Tomohisa Ishikawa, Yusuke Miyagawa, and Yosuke Hidaka (Department of Neuropsychiatry, Graduate School of Medical Science, Kumamoto University, Cyuo-ku, Kumamoto, Japan).
